# A Meta‐Analysis of Functional Magnetic Resonance Imaging Studies on In‐Group and Out‐Group Categorization

**DOI:** 10.1002/brb3.71314

**Published:** 2026-03-31

**Authors:** Kelly H. L. Sng, Xavier Y. H. Lim, Annabel S. H. Chen, Gianluca Esposito

**Affiliations:** ^1^ Neuroscience, Interdisciplinary Graduate Programme Nanyang Technological University Singapore Singapore; ^2^ Psychology Program, School of Social Sciences Nanyang Technological University Singapore Singapore; ^3^ Centre For Research and Development in Learning (CRADLE) Nanyang Technological University Singapore Singapore; ^4^ Lee Kong Chian School of Medicine Nanyang Technological University Singapore Singapore; ^5^ Office of Educational Research National Institute of Education Singapore Singapore; ^6^ Department of Psychology and Cognitive Science University of Trento Rovereto Italy

**Keywords:** activation likelihood estimation, fMRI, intergroup bias, meta‐analysis

## Abstract

**Introduction:**

Systematic variations in neural activation patterns during processing of group categorization is a facet often overlooked in extant meta‐analyses that typically treated all group memberships and task types as homogenous entities. Recognizing the necessity to differentiate between various group memberships and task types due to them eliciting distinct neural activity, our study addressed this gap by conducting a more fine‐grained exploration.

**Methods:**

A comprehensive meta‐analytic approach using the activation likelihood estimation (ALE) following PRISMA guidelines was employed. Inclusion criteria were task‐fMRI studies, whole‐brain analyses in a standard stereotaxic space, involved healthy adults, and published in English. Meta‐analyses were performed using GingerALE.

**Results:**

Overall in‐group > out‐group (IG>OG) processing included 66 studies (108 contrasts, 518 foci), and ALE analysis revealed 22 significant clusters, with the largest (3328 mm^3^) located in the left insula, inferior frontal gyrus, and uncus. Overall OG > IG processing included 35 studies (61 contrasts, 371 foci), and ALE analysis revealed 25 significant clusters, with the largest (1728 mm^3^) located in the right presupplementary motor area. Group membership classification found 38 ethnicity studies on in‐group bias (IG>OG; 59 contrasts, 279 foci), and ALE analysis revealed 27 significant clusters, with the largest (1616 mm^3^) located in the right superior occipital gyrus, right middle occipital gyrus, right middle temporal gyrus, and right fusiform gyrus. fMRI task types found 30 studies in empathy processing on in‐group bias (IG>OG; 50 contrasts, 260 foci), and ALE analysis revealed 23 significant clusters, with the largest (2480 mm^3^) located in the right postcentral gyrus, and right posterior cingulate gyrus.

**Conclusion:**

Our unrestricted search approach with ALE methodology extends beyond previous categorically constrained meta‐analyses by capturing group dynamics not limited to predefined categories. The distinct activation patterns for ethnicity‐based categorization and empathy processing tasks indicate multiple neural pathways rather than universal mechanisms for group processing. These context‐dependent patterns suggest interventions must target specific group‐task combinations rather than assume uniform neural processes.

## Graphical abstract

Activation likelihood estimation meta‐analysis of 168 functional magnetic resonance imaging experiments reveals distinct neural substrates for in‐group (left insula, inferior frontal gyrus) versus out‐group (right presupplementary motor area) processing. Different group types and tasks engage unique neural patterns, challenging universal mechanism models of intergroup bias.

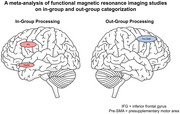

## 1 Introduction

The predominant neural evidence concerning group categorization has primarily centered around distinctions based on ethnic groups. Nevertheless, emerging findings indicate that group memberships of a nature distinct from ethnicity may prompt disparate forms of neural processing. Furthermore, extant meta‐analyses have yet to comprehensively explore variations in neural responses associated with different types of group categorization across various neuroimaging tasks. Consequently, an integrated meta‐analysis capable of pinpointing consistent patterns of neural activation specific to distinct group memberships and task types becomes imperative.

Several features mark distinctions between different group memberships. For instance, different groups are represented differently in terms of their closeness to the self (Smith et al. 1999; Uleman et al. 2002), their sizes, and the fluidity between the in‐group and the out‐group (Greenaway et al. 2015). In fact, groups that are more fluid may involve relatively weaker and less stabilized belief systems, therefore they may foster greater adaptability and receptivity to changes in group dynamics and affiliations. This fluidity can engender a more permeable boundary between the in‐group and the out‐group.

Moreover, certain group memberships are made obvious explicitly (e.g., ethnicity), which can be identified by mere visual inspection of body appearance, while other groups are much less obvious (e.g., nationality, affiliation) which often require higher‐level cognitive processing and understanding of others. Such distinctions between group memberships should intuitively lead to inferences that neural processing differs for different types of group memberships. For instance, neural activity has been found stronger toward minimal than ethnic groups as individuals observed members of their own minimal group or other group, and members of their own race or other race, receive either painful or non‐painful touch (Contreras‐Huerta et al. 2013). We acknowledge that much of literature on group memberships has focused on ethnicity, perhaps because it can be tested more easily, than less obvious and fluid groups such as nationality. Subsequently, meta‐analyses addressing group categorization have primarily concentrated on ethnic groups, neglecting the exploration of distinctions related to other forms of group memberships.

A case in point included meta‐analyses, notably the work of Molenberghs and Louis (2018), which have outlined neural substrates implicated in in‐group bias. In‐group bias refers to the differences in mental processing and responses triggered by individuals associated with one's in‐group compared to those associated with out‐groups or relevant cues. Molenberghs and Louis's (2018) analysis proposed that in‐group bias permeates various stages of information processing, ranging from initial responses, such as heightened amygdala activity, to more complex cognitive processes involving regions like the inferior frontal cortex. Key brain regions associated with in‐group bias, as suggested, included the amygdala, often linked with emotional responses and initial processing of social stimuli. In addition, higher‐level cognitive processing regions such as the inferior frontal cortex play a role in regulating or modulating these biases arising from social categorizations. While their analysis indirectly suggested that regions like the anterior cingulate cortex and lateral prefrontal cortex might be involved in processing cues related to out‐group members, little has been elaborated on it. Therefore, extensive delineation of neural substrates exclusively associated with out‐group discrimination would require further comprehensive investigations or dedicated meta‐analyses.

A recent comprehensive meta‐analysis conducted by Saarinen et al. (2021) aimed to elucidate the neural substrates associated with intergroup bias across various social group categorizations. Employing seed‐based *d* mapping (SDM), Saarinen et al. systematically synthesized data from an extensive number of functional imaging studies to investigate the neural correlates linked to diverse social group memberships, encompassing distinctions among ethnic, national, political, and trivial (minimal) groups. While affirming previously identified brain regions implicated in intergroup bias, such as the medial prefrontal cortex and insula, this analysis also revealed significant activations in several brain regions not previously associated with intergroup bias, including the cerebellum and supramarginal gyrus. Moreover, Saarinen et al. differentiated neural activity based on distinct types of functional MRI tasks. These tasks encompassed a range of cognitive processes including face processing, empathy‐related processing, moral processing, social judgment, memory, learning, and higher‐level cognitive processing. Furthermore, while trivial group membership exhibited a limited neural bias primarily localized in the left cingulate cortex, “real” groups (such as ethnic, national, or political categories) elicited activations across broader brain regions, including the cingulate cortex, superior temporal gyrus, superior parietal gyrus, and cerebellum. During face processing, heightened amygdala reactivity toward out‐groups was observed, reflecting a primary threat response and an enhanced alarm system in response to out‐group stimuli. Similarly, empathy‐related processing revealed differential activations in brain regions associated with empathic concern and mirror neuron activation between in‐group and out‐group perceptions. In essence, Saarinen et al.’s meta‐analysis underscored the multifaceted nature of intergroup bias, portraying differential neural activations across diverse group memberships and cognitive processes.

### 1.1 Objectives of the Current Study

Molenberghs and Louis's review, although comprehensive in its analysis, might have been constrained by its focus on specific groups and task types. While their work provided valuable insights into the neural correlates of in‐group bias, it might not have encompassed other group categorizations and task variations that have emerged in functional neuroimaging research, including ethnic groups and empathy task types that the present meta‐analysis aimed to cover. On the other hand, Saarinen et al.’s meta‐analysis, while laudable in its approach, primarily focused on specific groups (ethnic, national, political, and trivial) and particular types of tasks (face, empathy, and cognitive processing) within fMRI studies. Their study might have limited its scope by concentrating on these specific categories, potentially leaving out other group dynamics and task variations. The present meta‐analysis employed an unrestricted search strategy to capture group dynamics beyond predetermined categories and incorporated studies through 2025 to provide a comprehensive examination of the current literature.

The present study aimed to meta‐analyze fMRI studies investigating group categorization, and thereby summarize the neural substrates for the bias toward the in‐ over the out‐group (IG>OG) and against out‐ over the in‐group (OG>IG) across different tasks, depending on the modality involved, with the activation likelihood estimation (ALE). The ALE has two aims: (1) summarize the neural substrates for overall IG>OG and OG>IG processing, and (2) provide additional information on ethnicity as group membership, and empathy processing as task. Finally, we discussed the findings considering current in‐group and out‐group bias neural substrates.

## 2 Method

The method and analysis plan for the study have been preregistered on The Open Science Framework (OSF; https://osf.io/8x4wt).

### 2.1 Literature Selection

**FIGURE 1:**
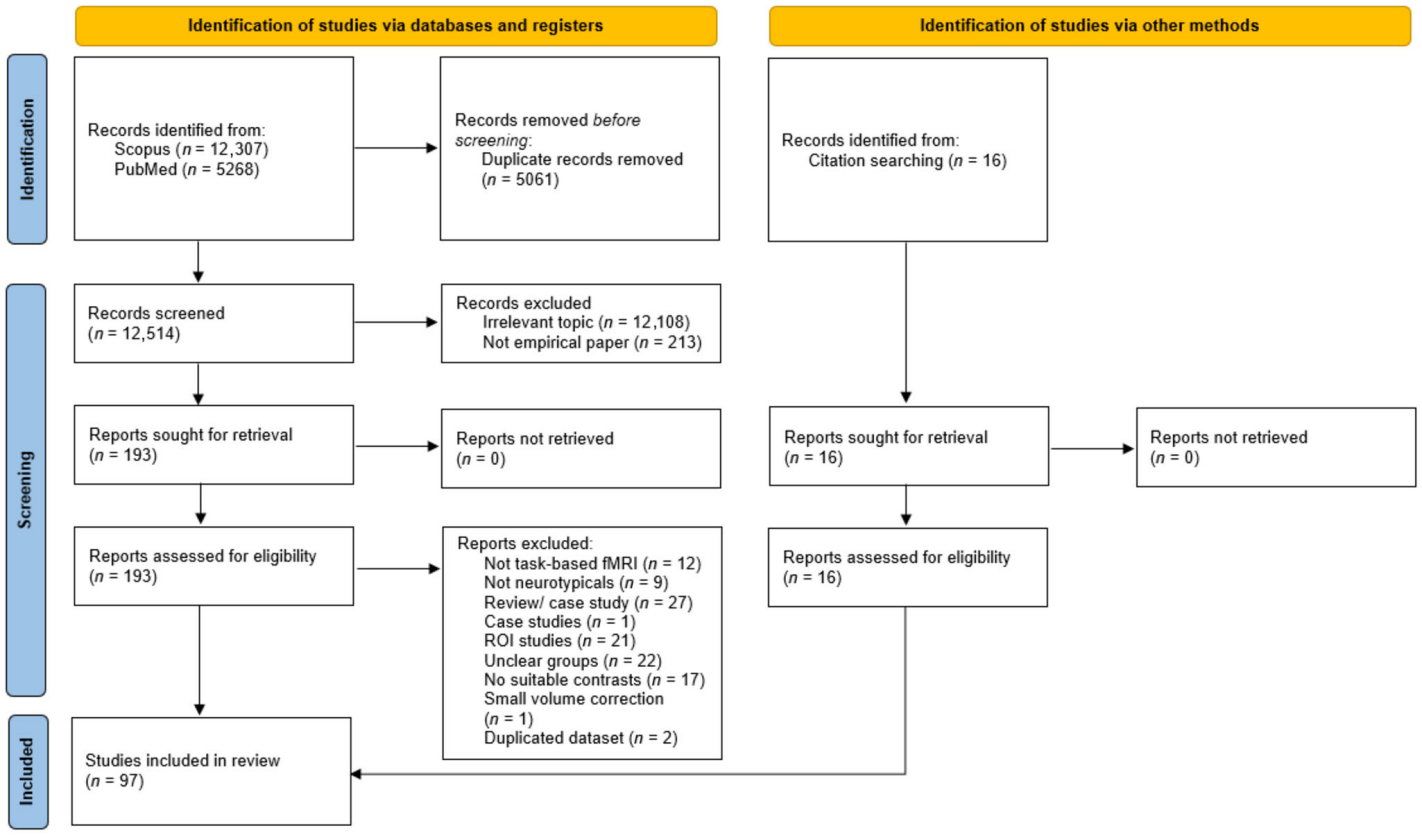
PRISMA flow diagram showing study identification, screening, and inclusion. The initial search was performed on July 11, 2023 and identified 14,673 records; the updated search (for records published between July 11, 2023 and September 1, 2025) was performed on September 1, 2025 and identified 2902 records. Numbers shown are cumulative after deduplication. fMRI = functional magnetic resonance imaging; ROI = region‐of‐interest.

The article selection process is illustrated in Figure 1. We performed a systematic literature search on PubMed and Scopus using the following search terms: (“ingroup” OR “outgroup” OR “group member*” OR “bias” OR “ingroup favoritism” OR “prejudice” OR “discrimination”) AND (“fMRI” OR “functional MRI” OR “neuroimaging” OR “BOLD”) to identify peer‐reviewed fMRI studies. Moreover, articles were also identified from previous meta‐analyses on group categorization and by searching through the reference lists and citation indices of studies obtained. The initial literature search ended on July 11, 2023, and a second round of search was conducted on September 1, 2025 on articles published between July 11, 2023 and September 1, 2025. Out of a cumulative 17,284 articles identified after deduplication between searches, articles were assessed according to the following inclusion criteria, previously established as best‐practice recommendations for neuroimaging meta‐analyses (Müller et al. 2018):

1. fMRI task‐based studies,
2. Results on whole‐brain analyses,
3. Studies reporting neural activations in a standard stereotaxic template (i.e., Talairach or MNI),
4. Studies on healthy adult populations, and
5. Studies published in English.

The following studies were excluded: (1) neuroimaging studies using non‐fMRI modalities (e.g., MEG, EEG, TMS), brain connectivity, or resting‐state studies, (2) studies based on region‐of‐interest, small‐volume correction, and masked analyses, (3) review/case studies, and (4) studies examining group versus self.

After further removal of duplicates (4686 articles), all identified articles (12,598 articles) were screened based on the title and abstract and defined as eligible/ineligible for the meta‐analysis. After reviewing the titles and abstracts, the identified full‐text articles (210 articles) were screened more precisely on the basis of the inclusion/exclusion criteria. Included articles were then categorized into (1) in‐group or out‐group categories according to the neural contrasts examined, (2) group membership delineated by participants’ in‐group and the types of groups exposed to, and 3) fMRI task categories applied.

### 2.2 Study Categorization

Evaluation of study quality occurred concomitantly with data extraction, utilizing a pre‐established standardized data extraction form that incorporated essential elements for study replication, aligning with the prevailing recommendations for fMRI studies (Nichols et al. 2017; Yeung et al. 2023). We assessed the quality of included studies on five domains (Supporting Information S1, Table S1.7): (1) sample size (< 15 = 0 unless block design due to higher statistical power which would then be given 1 point, 15–25 = 1, > 25 = 2 points), (2) scanner field strength (1.5T = 0, 3T = 1, >3T = 2 points), (3) analysis software version (non‐validated versions = 0, current standard = 1 point), (4) smoothing kernel appropriateness (> 3× voxel size, or not reported = 0, 2–3× voxel size = 1 point), and (5) statistical correction methods (uncorrected or not reported = 0, cluster extent threshold = 1, FWE/FDR or other forms of correction = 2 points). The median quality score was 6 (range: 3–8), with notable heterogeneity in sample sizes and statistical thresholds across studies, ranging from conservative (voxel‐wise FWE) to lenient (uncorrected with small cluster extents). In addition to whether the results reflected in‐group or out‐group categories, the type of group membership, and fMRI task employed, we collected information on participant demographics and study country. All included studies were required to meet stringent inclusion criteria aligned with best‐practice recommendations for neuroimaging meta‐analyses (Müller et al. 2018). Quality assessment and the extraction of outcome measures from studies were carried out independently by two researchers (K.H.L.S. and X.Y.H.L.). Any discrepancies were resolved through discussion.

We first verified whether the study distinctly identified in‐group and out‐group categories and if the reported contrasts specifically reflected in‐group > out‐group (IG>OG) (i.e., in‐group bias) or out‐group > in‐group (OG>IG). Subsequently, we assigned studies to relevant categories based on the participants' group membership and the group to which they were experimentally exposed, elucidating the focus of group membership under investigation. In cases involving multiple group memberships (e.g., Morrison et al. 2012), studies were classified under the category “Others.” Finally, considering the task paradigm and the specific contrast being analyzed, we categorized the studies based on the type of fMRI task. For instance, while a study could report the examination of empathy processing (e.g., watching faces of group members be poked by a needle (Cao et al. 2015), if the contrast under study was passive viewing of in‐group versus out‐group faces, it was categorized under “Face Processing”.

Table 1 provides a summary of the selected studies and contrasts included in the present meta‐analysis. Among the selected articles, some included more than one type of group membership, fMRI task, and/or conditions, which allowed implementation of more than one contrast (or study) in an article. Thus, we categorized the studies according to the targeted components of the contrasts from which the reported activation peaks were obtained. All contrasts have a clear indication of in‐group versus out‐group, and its direction of effect. Note that 108 contrasts from 66 studies with a total of 518 foci were included in the in‐group category. Sixty‐one contrasts from 35 studies with a total 371 foci were included in the out‐group category.

**TABLE 1:** Summary of included studies.

(a) In‐group > out‐group					
Study	Group membership	Type of fMRI task	*N*	Contrast	Number of foci
Azevedo et al. (2013)	Ethnicity	Implicit processing	27	Main effect of in‐group (i.e., in‐group stimuli > out‐group stimuli) [not differentiated between pain and touch stimuli]	2
Azevedo et al. (2013)	Ethnicity	Empathy processing	14	Observing in‐group members in pain > out‐group members in pain	1
Baumgartner et al. (2012)	Affiliation	Decision‐making	16	Observing punishment of in‐group > punishment of out‐group (in‐group effects for punishment network)	3
Baumgartner et al. (2012)	Affiliation	Empathy processing	16	Mentalizing decisions of in‐group > out‐group (in‐group effects for mentalization network)	14
Berlingeri et al. (2016)	Ethnicity	Empathy processing	25	In‐group differential empathic activation for race effect during stimulus phase (painful > neutral)	3
Bestelmeyer et al. (2015)	Nationality	Implicit processing	40	In‐group > out‐group accent	4
Brown et al. (2017)	Ethnicity	Implicit processing	19	Memory encoding‐related activity for same‐ (in‐group) > other‐race (out‐group)	1
Bruneau and Saxe (2010)	Nationality	Decision‐making	16	Among Arab participants: Pro‐Arab (in‐group) > pro‐Israel (out‐group) statements	6
Bruneau and Saxe (2010)	Nationality	Decision‐making	16	Among Israeli participants: Pro‐Israel (in‐group) > pro‐Arab (out‐group) statements	5
Cao et al. (2015)	Ethnicity	Empathy processing	30	Observing Chinese (in‐group) in pain > observing Caucasian (out‐group) in pain (interaction effects comparing neural empathic activation to Chinese > Caucasian faces)	1
Carollo et al. (2023)	Ethnicity	Face processing	43	(1) Viewing Chinese (in‐group) faces > Arabic + Indian + Caucasian (out‐groups) faces (2) Viewing Chinese (in‐group) faces > Indian (out‐group) faces (3) Viewing Chinese (in‐group) faces > Arabic (out‐group) faces	13
Chen et al. (2015)	Ethnicity	Empathy processing	22	Main effect of Group: Viewing in‐group emotional faces > viewing out‐group emotional faces	6
Cheon et al. (2011)	Ethnicity	Empathy processing	27	(1) In‐group bias in empathy (2) In‐group bias in empathy for Caucasian > Korean (3) In‐group bias in empathy for Korean > Caucasian	16
Chiao et al. (2008)	Ethnicity	Empathy processing	20	Own‐culture fear > other‐culture Fear	8
Contreras et al. (2013)	Ethnicity	Face processing	17	Main effect of race: Categorizing White faces > Black faces	5
Contreras‐Huerta et al. (2013)	Ethnicity	Empathy processing	20	Stimuli × Race interaction (in‐group bias): Observed painful > non‐painful touch in actors of the same race (Caucasian, in‐group) > actors of the other race (Chinese, out‐group)	1
Cui et al. (2023)	Affiliation	Decision‐making	28	(1) In‐group bias under scarcity (allocating scarce resource to in‐group > out‐group members) (2) Group membership × resource information interaction ([scarcity‐in‐group > scarcity‐outgroup] > [abundance‐in‐group > abundance‐outgroup]) [allocating scarce resource to in‐group members > out‐group members and abundant resources]	12
Cunningham et al. (2004)	Ethnicity	Face processing	13	Greater response to White > Black faces (525‐ms condition)	2
E. A. Losin et al. (2012)	Gender	Empathy processing	19	Imitate gesture own gender (in‐group) > other gender (out‐group)	5
E. A. Losin et al. (2012)	Ethnicity	Empathy processing	20	Imitate gesture of EA (in‐group) > CH (out‐group)	11
E. A. Losin et al. (2012)	Ethnicity	Face processing	20	(1) Viewing EA portrait (in‐group) > CH portrait (out‐group) (2) Viewing EA portrait (in‐group) > AA portrait (out‐group)	27
E. A. Losin et al. (2012)	Ethnicity	Implicit processing	20	Observe gesture EA (in‐group) > CH (out‐group)	5
Earls et al. (2013)	Ethnicity	Empathy processing	20	Actor race × condition interaction: observing and imitating own‐race (Caucasian; in‐group) > other‐race (African‐American; out‐group)	5
Ebner et al. (2013)	Age	Face processing	62	Age of face × participant age interaction across facial expressions: (greater activity to own‐age > other‐age faces for both young and older adults)	11
Falk et al. (2012)	Affiliation	Empathy processing	23	Taking perspectives of one's own candidate (in‐group) > opponent (out‐group)	3
Farmer et al. (2020)	Ethnicity	Face processing	25	Viewing White faces (in‐group) > viewing Black faces (out‐group)	1
Feng et al. (2011)	Ethnicity	Face processing	30	Categorizing Chinese (in‐group) faces > categorizing Caucasian (out‐group) faces	4
Firat et al. (2017)	Ethnicity	Implicit processing	13	Experiment 2: Viewing middle‐class depictions of Whites (in‐group) > Blacks (out‐group)	1
Fourie et al. (2017)	Ethnicity	Empathy processing	38	(1) In‐group biases in activation for perceived physical pain, in‐group > out‐group (participant race × pain × victim race) (2) In‐group biases in activation for perceived social pain, in‐group > out‐group (participant race × distress × victim race)	16
Freeman et al. (2010)	Ethnicity	Decision‐making	16	Judgment × race effect: Selectivity for individuated judgments for Whites (in‐group) > Blacks (out‐group)	1
Gilbert et al. (2012)	Ethnicity	Decision‐making	16	(1) Judgment of White (in‐group) > Black (out‐group) faces (collapsed over trait and friendship judgments) (2) Friendship (evaluative) judgments of White (in‐group) > Black (out‐group) faces (3) Stereotype‐related trait judgment of White (in‐group) > Black (out‐group) faces	11
Handley et al. (2023)	Ethnicity	Empathy processing	58	Responding to White (in‐group) Reading the Mind in the Eyes (RME) test > responding Black (out‐group) Reading the Mind in the Eyes (RME) test	3
Hein et al. (2010)	Affiliation	Empathy processing	16	(High > low in‐group pain) > (High > low out‐group pain)	8
Hein et al. (2016)	Nationality	Empathy processing	18	Before intervention (control group): Responses to in‐group > out‐group pain	8
Hein et al. (2016)	Nationality	Empathy processing	20	Before intervention (experimental group): Responses to in‐group > out‐group pain	8
Jiang et al. (2018)	Nationality	Decision‐making	25	(1) Main effect of accent: In‐group > out‐group (regional + foreign) (2) In‐group (confidence > neutral) > out‐group/regional (confident > neutral)	5
Kim and Johnson (2015)	Affiliation	Empathy processing	24	Viewing in‐group preference > viewing out‐group preferences	7
Krosch and Amodio (2019)	Ethnicity	Decision‐making	30	Main effect of viewing: White (in‐group) > Black (out‐group) faces	1
Lau and Cikara (2017)	Affiliation	Decision‐making	22	Repetition enhancement: In‐group (democrats/eagles) > out‐group (republicans/rattlers)	4
Lau and Cikara (2017)	Minimal	Decision‐making	22	Repetition enhancement: In‐group (democrats/eagles) > out‐group (republicans/rattlers)	4
Lee et al. (2008)	Ethnicity	Face processing	13	Own‐race > other‐race neutral face	3
Lee et al. (2008)	Ethnicity	Empathy processing	13	Own‐race > other‐race emotional face (happy and sad)	6
Li et al. (2015)	Ethnicity	Empathy processing	20	Mortality salience group: Viewing in‐group in pain > out‐group	2
Li et al. (2015)	Ethnicity	Empathy processing	20	Negative affect group: Viewing in‐group in pain > out‐group	1
Li et al. (2016)	Ethnicity	Face processing	44	Viewing White (in‐group) face > viewing Black (out‐group) face	3
Li et al. (2016)	Ethnicity	Implicit processing	44	Viewing White (in‐group) face with positive trait > Black (out‐group) face with positive trait	5
Li et al. (2020)	Minimal	Empathy processing	31	Group‐based guilt: Observe in‐group in pain ​> ​out‐group	6
Lieberman et al. (2005)	Ethnicity	Face processing	20	Viewing in‐group face > out‐group face	4
Lin et al. (2018)	Ethnicity	Decision‐making	45	Aligning ratings during the social influence task to in‐group > out‐group members	17
Luo et al. (2015)	Ethnicity	Empathy processing	30	G/G genotype individuals: Viewing Asian (in‐group) > Caucasian (out‐group) faces in pain	1
Marsh et al. (2016)	Minimal	Empathy processing	24	(1) Imitative compatibility toward IG > OG (incompatible > compatible) (2) Spatial compatibility toward IG > OG (incompatible > compatible) (3) General compatibility (spatial and imitative compatibility were consistent [both compatible, or both incompatible]) toward IG > OG (incompatible > compatible)	30
Mathur et al. (2010)	Ethnicity	Empathy processing	14	African‐American: Observing in‐group in pain > observing out‐group in pain	12
Mathur et al. (2010)	Ethnicity	Empathy processing	14	Caucasian‐American: Observing in‐group in pain > observing out‐group in pain	18
Mathur et al. (2010)	Ethnicity	Empathy processing	28	Observing in‐group in pain and no pain > observing out‐group in pain and no pain	2
Mathur et al. (2012)	Ethnicity	Empathy processing	10	Main effect of racial group: Viewing African‐American (in‐group) in pain > Caucasian‐American (out‐group) in pain	11
Mathur et al. (2012)	Ethnicity	Empathy processing	10	Main effect of racial group: Viewing Caucasian‐American (in‐group) in pain > African‐American (out‐group) in pain	13
Mattan et al. (2018)	Ethnicity	Face processing	60	Main effect of race: Viewing White (in‐group) face > viewing Black (out‐group) face	1
Mauchaund et al. (2023)	Nationality	Implicit processing	24	In‐group > out‐group (irrespective of prosody)	14
Mei et al. (2025)	Ethnicity	Empathy processing	80	In‐group > Out‐group for pain (> non‐pain) stimulations	11
Mitchell et al. (2006)	Affiliation	Decision‐making	15	Judgment of similar others (in‐group, liberal/conservative [dependent on participant] target) > judgment of dissimilar others (out‐group, liberal/ conservative [dependent on participant] target)	6
Molapour et al. (2015)	Ethnicity	Implicit processing	20	(1) Acquisition: Overall activity (White > Black) (2) Extinction: Overall activity (White > Black) (3) Acquisition: Linear change over time: White shock > Black shock	9
Molenberghs and Morrison (2014)	Minimal	Decision‐making	20	Categorizing “My Team” > categorizing “Other Team” condition	1
Molenberghs et al. (2014)	Minimal	Decision‐making	48	Rewarding in‐group > out‐group	3
Morrison et al. (2012)	Others	Implicit processing	20	Viewing words related to in‐group > out‐group	3
Newman‐Norlund et al. (2009)	Affiliation	Implicit processing	22	(1) Observation of in‐group > out‐group members (2) Observation of errors of in‐group > out‐group members	7
Nugiel and Beer (2020)	Affiliation	Decision‐making	50	In‐group > out‐group evaluation	2
Rauchbauer et al. (2015)	Ethnicity	Empathy processing	41	In‐group (incongruent > congruent) masked inclusively with in‐group (incongruent > congruent) > out‐group (incongruent > congruent)	1
Richins et al. (2019)	Affiliation	Implicit processing	69	Main effect target group: (Exeter > Cardiff) + (Sussex > Cardiff) (not differentiated between pain and no‐pain conditions)	27
Ronquillo et al. (2007)	Ethnicity	Face processing	11	Interaction ([viewing White {in‐group} face in dark tone > light tone] > [viewing Black {out‐group} face in dark tone > light tone])	5
Rubien‐Thomas et al. (2021)	Ethnicity	Face processing	106	Stimulus race by participant race interaction: Black > White participants in Black > White faces	4
Ruckmann et al. (2015)	Minimal	Implicit processing	30	Main effect of condition (in‐group > out‐group) [not differentiated between pain and no‐pain conditions]	4
Ruckmann et al. (2015)	Minimal	Empathy processing	30	Interaction between pain × condition (observing in‐group in pain > out‐group in pain)	7
Scheepers et al. (2013)	Nationality	Face processing	41	Viewing in‐group face > viewing out‐group face	4
Sheng et al. (2014)	Ethnicity	Empathy processing	21	Race judgments: Asian (in‐group) face (pain > neutral) > Caucasian (out‐group) face (pain > neutral)	1
Telzer et al. (2015)	Ethnicity	Decision‐making	26	Donating to in‐group members > donating to out‐group members	1
Van Bavel et al. (2008)	Minimal	Face processing	17	Main effect of team (greater activity in response to novel in‐group > novel out‐group faces)	6
Volz et al. (2009)	Minimal	Decision‐making	20	Assigning money in in‐group (mono‐chrome matrices in the color of the in‐group) > out‐group (monochrome matrices in the color of the out‐group) trials	3
Wang et al. (2015)	Ethnicity	Empathy processing	30	Priming × race interaction analysis of the contrast of painful > non‐painful stimuli (in‐group > out‐group)	3
Watson and de Gelder (2017)	Nationality	Empathy processing	21	(1) Both Tasks: White (in‐group) angry bodies > Black (out‐group) angry bodies (2) Emotion categorization task: White (in‐group) bodies > Black (out‐group) bodies (3) Shape categorization task: White (in‐group) angry bodies > Black (out‐group) angry bodies	11
Wu et al. (2018)	Affiliation	Decision‐making	54	Playing binary trust game with person of the same political identity > different political identity (SAME‐DM2 > DIFF‐DM2)	2

(b) Out‐group > In‐group					
Study	Group membership	Type of fMRI task	*N*	Contrast	Number of foci
Baumgartner et al. (2012)	Affiliation	Decision‐making	16	Observing punishment of out‐group > punishment of in‐group (out‐group effects for punishment network)	9
Baumgartner et al. (2012)	Affiliation	Empathy processing	16	Mentalizing decisions of out‐group > in‐group (out‐group effects for mentalization network)	8
Berlingeri et al. (2016)	Ethnicity	Empathy processing	25	Out‐group differential empathic activation for race during response phase (painful > neutral)	7
Bruneau and Saxe (2010)	Nationality	Decision‐making	16	Among Arab participants: Pro‐Israel (out‐group) > pro‐Arab (in‐group) statements	7
Bruneau and Saxe (2010)	Nationality	Decision‐making	16	Among Israeli participants: Pro‐Arab (out‐group) > pro‐Israel (in‐group) statements	7
Bruneau et al. (2012)	Nationality	Empathy processing	24	Emotional pain of conflict out‐group > in‐group member (Arab > Israeli)	1
Cheon et al. (2011)	Ethnicity	Empathy processing	27	Out‐group bias in empathy	2
Contreras et al. (2013)	Ethnicity	Face processing	17	Main effect of race: Categorizing Black faces > White faces	2
Cunningham et al. (2004)	Ethnicity	Face processing	13	(1) Greater response to Black > White faces (30‐ms condition) (2) Greater response to Black > White faces (525‐ms condition)	13
E. A. Losin et al. (2012)	Ethnicity	Empathy processing	20	Imitate gesture AA (out‐group) > EA (in‐group)	17
Falk et al. (2012)	Affiliation	Empathy processing	23	Taking perspective of opponent's candidate (out‐group) > one's own candidate (in‐group)	5
Fox et al. (2013)	Affiliation	Empathy processing	16	Processing hateful people (out‐group; neo‐Nazis) in pain > processing likeable people (in‐group) in pain	20
Izuma et al. (2019)	Nationality	Implicit processing	70	Viewing images related to South Korea (out‐group) > Japan (in‐group)	2
Jiang et al. (2018)	Nationality	Decision‐making	25	(1) Main effect of accent: Out‐group (regional + foreign) > in‐group (2) Out‐group/regional (confident > neutral) > in‐group (confident > neutral) (3) Out‐group/regional (confident > doubtful) > in‐group (confident > doubtful) (4) Out‐group/foreign (confident > neutral) > in‐group (confident > neutral) (5) Out‐group/foreign (confident > doubtful) > in‐group (confident > doubtful) (6) Out‐group/foreign (doubtful > neutral) > in‐group (doubtful > neutral)	9
Kaplan et al. (2007)	Affiliation	Face processing	20	Viewing opposing candidate's (out‐group) face > own candidate's (in‐group) face	18
Krendl et al. (2009)	Affiliation	Face processing	65	Viewing stigmatized members' face (out‐group) > control face (in‐group)	8
Krosch and Amodio (2019)	Ethnicity	Decision‐making	30	Main effect of viewing: Black (out‐group) > White (in‐group) faces	3
Lau and Cikara (2017)	Affiliation	Decision‐making	22	Repetition suppression: Out‐group (republicans/rattlers) > in‐group (democrats/eagles)	6
Lau and Cikara (2017)	Minimal	Decision‐making	22	Repetition suppression: Out‐group (republicans/rattlers) > in‐group (democrats/eagles)	6
Lee et al. (2008)	Ethnicity	Face processing	13	Other‐race > own‐race neutral face	8
Lee et al. (2008)	Ethnicity	Empathy processing	13	Other‐race > own‐race emotional face (happy and sad)	5
Lieberman et al. (2005)	Ethnicity	Face processing	20	Viewing out‐group face > in‐group face	2
Liu et al. (2015)	Ethnicity	Empathy processing	26	(1) Main effect of group: Out‐group > in‐group (2) (viewing disgusted out‐group member > viewing neutral out‐group) > (viewing disgusted in‐group > viewing neutral in‐group)	19
Luo et al. (2015)	Ethnicity	Empathy processing	30	A/A genotype individuals: Viewing Caucasian (out‐group) > Asian (in‐group) faces in pain	1
Mathur et al. (2010)	Ethnicity	Empathy processing	28	Observing out‐group in pain and no pain > observing in‐group in pain and no pain	7
Mattan et al. (2018)	Ethnicity	Face processing	60	(1) Main effect of race: Viewing Black (out‐group) face > viewing White (in‐group) face (2) Status × race interaction	3
Mauchaund et al. (2023)	Nationality	Implicit processing	24	Out‐group > in‐group (irrespective of prosody)	21
Mauchaund et al. (2023)	Nationality	Empathy processing	24	Out‐group (complaining > neutral) > in‐group (complaining > neutral)	3
Mitchell et al. (2006)	Affiliation	Decision‐making	15	Judgment of dissimilar others (out‐group, liberal/conservative [dependent on participant] target) > judgment of similar others (in‐group, liberal/conservative [dependent on participant] target)	1
Molapour et al. (2015)	Ethnicity	Implicit processing	20	(1) Acquisition: Overall activity (Black > White) (2) Extinction: Overall activity (Black > White) (3) Acquisition: Linear change over time: Black shock > White shock (4) Extinction: Linear change over time: Black shock > White shock	49
Moradi et al. (2017)	Affiliation	Implicit processing	20	Mismatch trials: Out‐group > in‐group	6
Nugiel and Beer (2020)	Affiliation	Decision‐making	50	Out‐group > in‐group evaluation	3
Rauchbauer et al. (2015)	Ethnicity	Empathy processing	41	Out‐group (incongruent > congruent) masked inclusively with out‐group (incongruent > congruent) > in‐group (incongruent > congruent)	7
Richeson et al. (2003)	Ethnicity	Face processing	15	Experiment 1: Viewing Black face > viewing White face	10
Ronquillo et al. (2007)	Ethnicity	Face processing	11	Viewing Black (out‐group) face > viewing White (in‐group) face	8
Rule et al. (2010)	Ethnicity	Face processing	28	Across all participants: Target × participant interaction: Observing out‐group > in‐group faces	2
Van Bavel et al. (2008)	Ethnicity	Face processing	17	Main effect of race: Observing Black (out‐group) > White (in‐group) faces	5
van Gils et al. (2020)	Nationality	Decision‐making	17	Sacrifice out‐group > in‐group	1
Watson and de Gelder (2017)	Nationality	Empathy processing	21	(1) Both tasks: Black (out‐group) bodies > viewing White (in‐group) bodies (2) Both tasks: Black (out‐group) happy bodies > White (in‐group) happy bodies (3) Emotion categorization task: Black (out‐group) bodies > White (in‐group) bodies (4) Emotion categorization task: Black (out‐group) happy bodies > White (in‐group) happy bodies (5) Shape categorization task: Black (out‐group) bodies > White (in‐group) bodies (6) Shape categorization task: Black (out‐group) happy bodies > White (in‐group) happy bodies	65

*Note*: Included studies of (a) in‐group > out‐group had minimum *N* = 10, maximum *N* = 106, median *N* = 22; and of (b) out‐group > in‐group had minimum *N* = 11, maximum *N* = 70, median *N* = 20.5.

Fifty nine IG>OG contrasts from 38 studies with a total 279 foci, and 28 OG>IG contrasts from 19 studies with a total 171 foci were included in the ethnicity group. Ethnicity refers to comparison between races and ethnic groups, for instance, making comparisons between African‐American versus Caucasian‐American (Mathur et al. 2012). Tasks in this group included: performing a Reading the Mind in the Eyes (RME) task, where participants viewed cropped images of in‐group/out‐group members’ eyes and selected which emotions those eyes conveyed (e.g., Handley et al. 2023) and impression formation tasks (e.g., Mattan et al. 2018). Although underpowered due to a low number of studies, we labeled the remaining studies into distinct group categories. Fifteen IG>OG contrasts from 8 studies with a total 64 foci, and 1 OG>IG contrasts from 1 study with a total 6 foci were included in the minimal group. Studies with minimal groups involved random assignment of participants to arbitrary categories (e.g., team red vs. team blue, Marsh et al. 2016). Tasks in this group included: performing moral evaluation tasks where participants allocated rewards and punishments to in‐group and out‐group members (e.g., Molenberghs et al. 2014) and passive viewing of hand actions performed by in‐group/out‐group members (e.g., Molenberghs et al. 2013). Eighteen IG>OG contrasts from 11 studies with a total 95 foci, and 14 OG>IG contrasts from 9 studies with a total 84 foci were included in the affiliation group. Studies included established interdependent groups (e.g., own platoon vs. other platoon, Baumgartner et al. (2012) or political groups (e.g., conservatives vs. liberal, Mitchell et al. 2006) which participants identify with. Twelve IG>OG contrasts from 7 studies with a total 65 foci, and 19 OG>IG contrasts from 7 studies with a total 116 foci were included in the nationality group, which examined distinct nationalities (e.g., Chinese vs. Korean, Feng et al. (2017). Finally, one study was included in the gender group, one study in the age group, and one study labeled as others, as it involved more than one type of group memberships. An exploratory meta‐analysis of the OG>IG comparison for ethnicity group, as well as a meta‐analysis of the nationality group are reported in the Supporting Information S2. The remaining groups were not reviewed due to a lack of studies.

Fifty IG>OG contrasts from 30 studies with a total 260 foci, and 24 OG>IG contrasts from 14 studies with a total 167 foci were included in the empathy processing task, which involved engagement of theory of mind processes (including viewing pain induction, (Fox et al. 2013), and imitation and mentalization of group members (Decety and Jackson 2004; Singer and Lamm 2009). Although underpowered, we labeled the remaining studies into distinct task types. Nineteen IG>OG contrasts from 15 studies with a total 93 foci, and 15 OG>IG contrasts from 12 studies with a total 80 foci were included in the face processing task, which involved viewing visual depictions of group members’ facial features. Twenty‐two IG>OG contrasts from 16 studies with a total 93 foci, and 15 OG>IG contrasts from 12 studies with a total 80 foci were included in the decision‐making task, which involved processing of scenarios assessing preferences and trade‐offs (Glimcher et al. 2009), including the allocation of rewards and punishments (e.g., deciding how to punish in‐group or out‐group member by reducing their monetary payoffs, (Feng et al. 2017). Finally, 17 IG>OG contrasts from 12 studies with a total 82 foci, and 7 OG>IG contrasts from 4 studies with a total 78 foci were included in the implicit processing task, which involved passive viewing and listening of stimuli. An exploratory meta‐analysis of the OG>IG comparison for empathy processing task, as well as a meta‐analysis of the face processing task are reported in the Supporting Information S2. The remaining task types were not reviewed due to a lack of studies.

### 2.3 Analyses

The meta‐analyses were conducted utilizing the revised algorithm of the ALE approach (Eickhoff et al. 2012; Eickhoff et al. 2009; Laird et al. 2005; Turkeltaub et al. 2002), implemented in GingerALE (available at https://brainmap.org/ALE). This method estimates the likelihood of activation convergence at specific coordinates based on foci extracted from selected studies, revealing consistent neural substrates rather than functional connectivity networks. Foci were modeled as centers of three‐dimensional Gaussian spatial probability distributions, accounting for spatial uncertainty. The revised algorithm, as detailed in Eickhoff et al. (2009), estimates spatial uncertainty using empirical data on between‐subject and between‐template variances, influencing the width of the modeled probability distributions. Notably, to accommodate between‐subject variances, the width of probability distributions (i.e., full‐width at half‐maximum, FWHM) is determined by the number of subjects in each experiment, resulting in smaller FWHMs for experiments with larger samples. The probability distributions of all foci in an experiment are combined into a modeled activation (MA) map, summarizing all foci. A single meta‐analysis combines the MA maps, and ALE scores are computed voxel‐by‐voxel, representing the likelihood of activation convergence at specific positions among the experiments. To discern true convergence from random convergence, ALE scores are compared with an analytically computed null‐distribution reflecting random spatial associations between experiments (Eickhoff et al. 2012). This comparison allows for random‐effect inference, a key modification from the previous ALE approach, focusing on above‐chance convergence between experiments (random‐effects) rather than convergence between foci within a single experiment (fixed‐effects).

In the current study, ALE analyses were conducted using the foci reported in the selected contrasts. Coordinates provided in the Talairach space were converted to MNI space, employing transformation algorithms integrated into GingerALE. Individual meta‐analyses were performed for overall 1) IG>OG, 2) OG>IG, 3) ethnicity group (IG>OG), 4) empathy processing task (IG>OG). All other group memberships and fMRI task‐types did not have enough studies for adequate power to conduct the ALE analyses. Statistical significance was assessed using *p* < 0.005 (uncorrected) with minimum cluster extent of 200 mm^3^. This approach was chosen to balance Type I and Type II errors given the exploratory nature of our subgroup analyses, whilst acknowledging the increased false positive risk. In addition, cluster‐level FWE correction was performed using a cluster‐forming threshold of *p* < 0.001 (uncorrected), cluster‐level threshold of *p* < 0.05 (FWE‐corrected), with 10,000 permutations (Eickhoff et al. 2012). Results are reported in Supporting Information S2, Table S2.5. The thresholded ALE maps were overlaid onto the colin27_T1_seg_MNI.nii brain template in the software MANGO (https://mangoviewer.com/).

## 3 Results

Ninety‐six fMRI studies were included in the meta‐analysis, of which 66 assessed overall IG>OG processing, and 35 assessed overall OG>IG processing. In addition, 38 explored ethnicity group (IG>OG), and 38 explored empathy processing task (IG>OG).

### 3.1 IG>OG Processing

**FIGURE 2:**
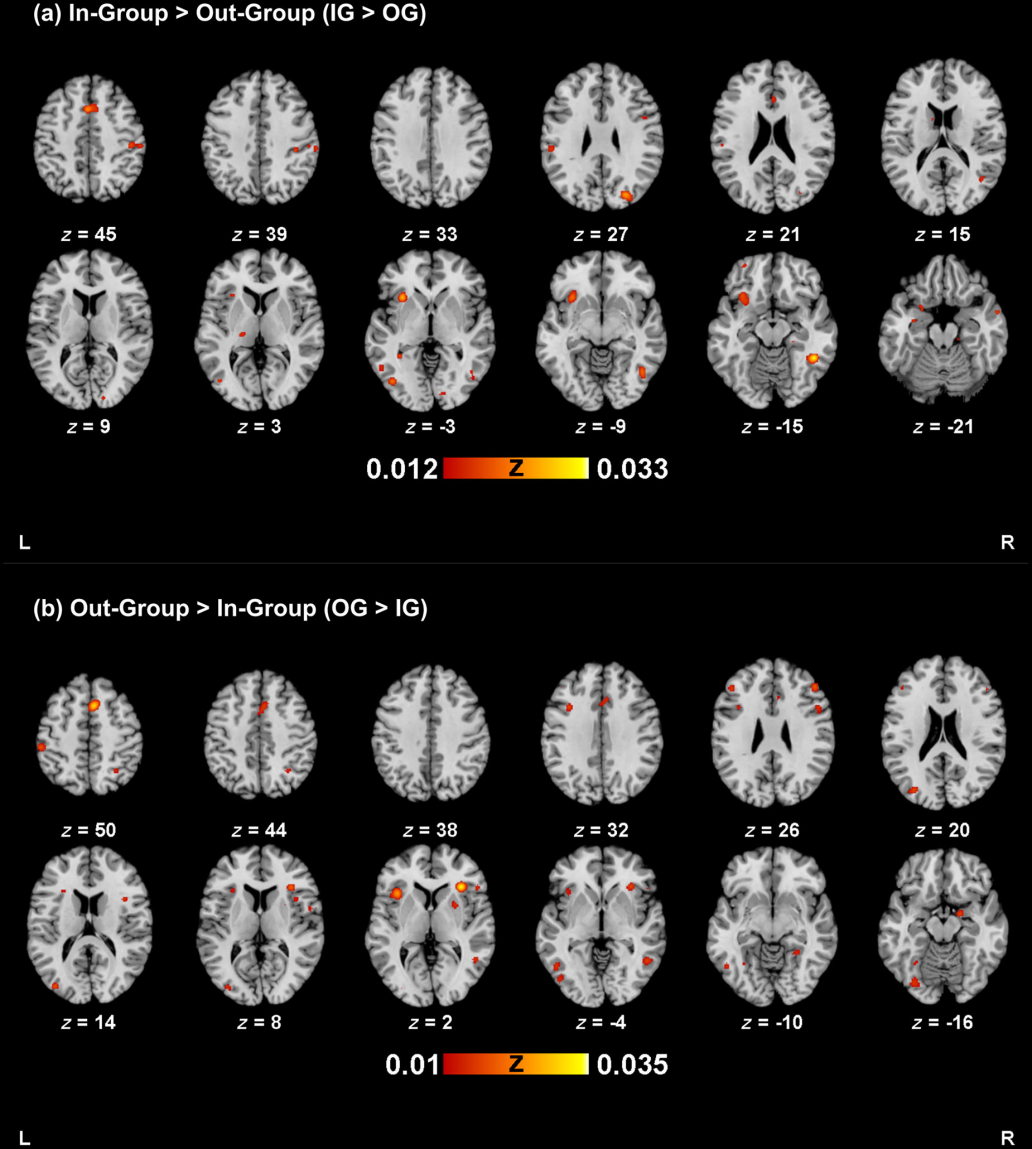
Activation map for (a) in‐group > out‐group processing, and (b) out‐group > in‐group processing. Activation maps were thresholded at *p *< 0.005 (uncorrected), with a minimum cluster size of 200 mm^3^. Images are in neurological convention.

Included studies had a median sample size of 20 (range: 10–106). Across all studies (*n* = 66), there were 108 experiments (2042 subjects, 518 foci, 12 out‐of‐mask foci) for bias toward in‐group (IG>OG) analysis. ALE analysis for IG>OG processing revealed 22 significant clusters (Table 2; Figure 2a), with the largest (3328 mm^3^) located in the left insula, inferior frontal gyrus (IFG), and uncus. Other clusters included the right fusiform gyrus, right inferior parietal lobule, left medial frontal gyrus, and right cuneus.

**TABLE 2:** ALE results of (a) in‐group > out‐group processing, and (b) out‐group > in‐group processing.

(a) In‐group > out‐group processing						
			MNI coordinates			
Cluster	Volume (mm^3^)	Max ALE (×10^−2^)	*x*	*y*	*z*	Label
1	3328	2.73	−32	18	−4	Left insula
		1.94	−32	20	−14	Left inferior frontal gyrus
		1.73	−24	8	−20	Left uncus
2	2472	3.28	46	−48	−16	Right fusiform gyrus
		2.17	46	−64	−8	Right fusiform gyrus
		1.59	38	−72	0	Right inferior occipital gyrus
3	1680	1.91	46	−26	46	Right inferior parietal lobule
		1.87	40	−30	38	Right inferior parietal lobule
		1.89	58	−26	44	Right inferior parietal lobule
4	1616	2.38	−2	14	44	Left medial frontal gyrus
		1.43	10	4	48	Right cingulate gyrus
5	1432	2.55	26	−80	28	Right cuneus
		1.50	32	−76	18	Right cuneus
6	776	2.34	−42	−74	−2	Left inferior occipital gyrus
7	680	1.74	−54	−30	28	Left inferior parietal lobule
		1.65	−56	−26	24	Left inferior parietal lobule
8	528	1.77	16	−28	−18	Right cerebellum
		1.65	22	−30	−18	Right cerebellum
9	512	1.89	−20	−24	0	Left thalamus
10	368	1.69	42	−62	16	Right middle temporal gyrus
11	368	1.60	50	6	24	Right inferior frontal gyrus
12	352	2.16	60	2	−18	Right middle temporal gyrus
13	320	1.60	−54	−58	−4	Left inferior temporal gyrus
14	320	1.75	−6	58	30	Left medial frontal gyrus
15	288	1.79	−30	54	−14	Left middle frontal gyrus
16	280	1.86	−32	−6	−24	Left parahippocampal gyrus/amygdala
17	256	1.85	−34	−46	−4	Left parahippocampal gyrus
18	256	1.77	16	−92	8	Right lingual gyrus
19	248	1.75	−8	6	12	Left caudate
20	248	1.67	2	26	20	Left anterior cingulate
21	232	1.90	−10	66	−6	Left medial frontal gyrus
22	200	1.53	12	−88	−2	Right lingual gyrus

(b) Out‐group > in‐group processing						
			MNI coordinates			
Cluster	Volume (mm^3^)	Max ALE (×10^−2^)	Label	*x*	*Y*	*z*
1	1728	3.11	6	14	50	Right presupplementary motor area
2	1697	3.48	34	26	4	Right insula
3	1640	2.47	−36	18	2	Left insula
		1.35	−28	22	12	Left insula
		1.08	−38	26	−8	Left inferior frontal gyrus
4	1264	1.85	−36	−84	12	Left middle occipital gyrus
		1.71	−32	−80	20	Left middle occipital gyrus
		1.50	−32	−86	6	Left middle occipital gyrus
5	1152	1.98	−52	−32	52	Left inferior parietal lobule
		1.26	−48	−42	56	Left inferior parietal lobule
6	816	1.67	−34	−80	−18	Left inferior occipital gyrus
7	712	1.88	50	−54	0	Right inferior temporal gyrus
8	672	1.86	48	34	26	Right middle frontal gyrus
9	632	1.65	18	−4	−16	Right parahippocampal gyrus
10	608	1.71	−50	−62	−6	Left middle occipital gyrus
11	600	1.48	6	20	30	Right cingulate gyrus
12	544	1.62	−44	34	24	Left middle frontal gyrus
13	528	1.89	−36	12	30	Left inferior frontal gyrus
14	408	1.57	30	−58	48	Right superior parietal lobule
15	392	1.65	28	6	4	Right putamen
16	392	1.47	50	12	26	Right inferior frontal gyrus
17	376	1.76	40	12	12	Right insula
18	352	1.60	−46	−76	−6	Left inferior occipital gyrus
19	304	1.45	−30	−58	−14	Left cerebellum
20	288	1.61	28	−46	−12	Right parahippocampal gyrus
21	272	1.45	52	24	2	Right inferior frontal gyrus
22	240	1.46	−16	−50	−48	Left cerebellum
23	216	1.51	18	−64	62	Right superior parietal lobule
24	208	1.33	56	0	6	right precentral gyrus
25	200	1.54	−12	−28	−44	Left pons

*Note*: All clusters reported survived a voxel‐level uncorrected threshold of *p *< 0.005, with a minimum cluster size of 200 mm^3^. Coordinates are reported in the Montreal Neurological Institute (MNI) convention.

Abbreviation: ALE: activation likelihood estimate.

### 3.2 OG>IG Processing

Included studies had a median sample size of 20.5 (range: 11–70). Across all studies (*n* = 35), there were 61 experiments (912 subjects, 371 foci, 12 out‐of‐mask foci) for bias against out‐group (OG>IG) analysis. OG>IG processing revealed 25 significant clusters (Table 2; Figure 2b), with the largest (1728 mm^3^) located in the right presupplementary motor area. Other clusters included the bilateral insula, left middle occipital gyrus, left inferior parietal lobule, left inferior occipital gyrus, and right inferior temporal gyrus.

### 3.3 Ethnicity Group (IG>OG)

**FIGURE 3:**
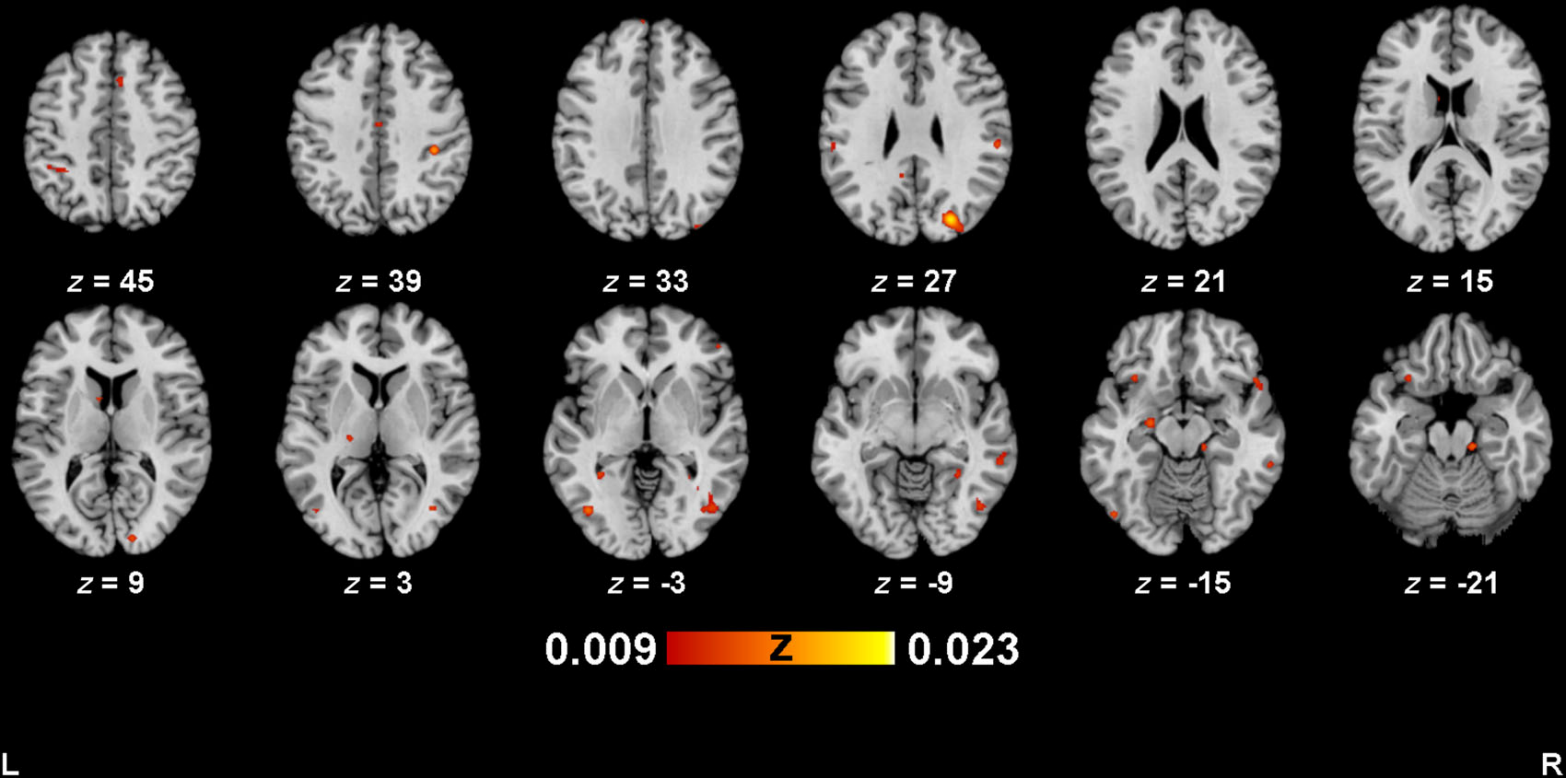
Activation map for ethnicity group, IG>OG. Activation maps were thresholded at *p *< 0.005 (uncorrected), with a minimum cluster size of 200 mm^3^. Images are in neurological convention.

Included studies had a median sample size of 20 (range: 10–106). Across all studies (*n *= 38), there were 59 experiments (1191 subjects, 279 foci, 8 out‐of‐mask foci) for bias toward ethnicity in‐group (IG>OG) analysis. Analysis for ethnicity (IG>OG) revealed 27 significant clusters (Table 3; Figure 3), with the largest (1616 mm^3^) located in the right superior occipital gyrus. Other clusters included the right middle occipital gyrus, right middle temporal gyrus, right fusiform gyrus, right cerebellum, and left IFG.

**TABLE 3:** ALE results of in‐group bias in ethnicity group.

Cluster	Volume (mm^3^)	Max ALE (×10^−2^)	MNI coordinates			Label
			*x*	*y*	*z*	
1	1616	2.32	26	−80	28	Right superior occipital gyrus
		1.42	34	−84	30	Right superior occipital gyrus
2	1560	1.59	38	−72	0	Right middle occipital gyrus
		1.32	46	−70	−2	Right middle temporal gyrus
		1.28	46	−70	−10	Right fusiform gyrus
		1.14	44	−62	−4	Right middle temporal gyrus
		1.04	52	−68	−12	Right fusiform gyrus
3	649	1.52	62	−40	−12	Right middle temporal gyrus
4	536	1.66	16	−28	−18	Right cerebellum
5	528	1.45	−34	20	−16	Left inferior frontal gyrus
		1.35	−30	20	−20	Left inferior frontal gyrus
6	528	1.68	−40	−72	−2	Left inferior occipital gyrus
7	520	1.42	30	−48	−6	Right parahippocampal gyrus
		1.01	36	−56	−4	Right parahippocampal gyrus
8	440	1.45	−22	−12	−14	Left amygdala
9	440	1.41	−4	28	54	Left superior frontal gyrus
10	416	1.39	52	18	−16	Right inferior frontal gyrus
		1.15	56	12	−14	Right superior temporal gyrus
11	408	1.51	−20	−22	0	Left thalamus/ventral posterior lateral nucleus
12	408	1.18	−32	−46	44	Left inferior parietal lobule
		1.17	−44	−42	44	Left inferior parietal lobule
13	384	1.80	38	−32	38	Right inferior parietal lobule
14	352	1.46	58	−28	26	Right inferior parietal lobule
15	328	1.55	−48	−76	−14	Left fusiform gyrus
16	320	1.59	36	−68	−28	Right cerebellum
17	320	1.21	−58	−26	24	Left postcentral gyrus
		1.10	−58	−38	30	Left inferior parietal lobule
18	304	1.21	−12	−86	−30	Left cerebellum
19	304	1.28	−2	60	32	Left medial frontal gyrus
20	288	1.37	−32	−48	−4	Left parahippocampal gyrus
21	280	1.59	−8	6	12	Left caudate
22	280	1.30	−6	−48	30	Left posterior cingulate
23	272	1.60	50	42	−6	Right inferior frontal gyrus
24	272	1.46	14	−92	8	Right lingual gyrus
25	240	1.26	16	−60	24	Right precuneus
26	208	1.30	0	−12	40	Left cingulate gyrus
27	208	1.23	6	18	46	Right medial frontal gyrus

*Note*: All clusters reported survived a voxel‐level uncorrected threshold of *p *< 0.005, with a minimum cluster size of 200 mm^3^. Coordinates are reported in the Montreal Neurological Institute (MNI) convention.

Abbreviation: ALE: activation likelihood estimate.

### 3.4 Empathy Processing Task (IG>OG)

**FIGURE 4:**
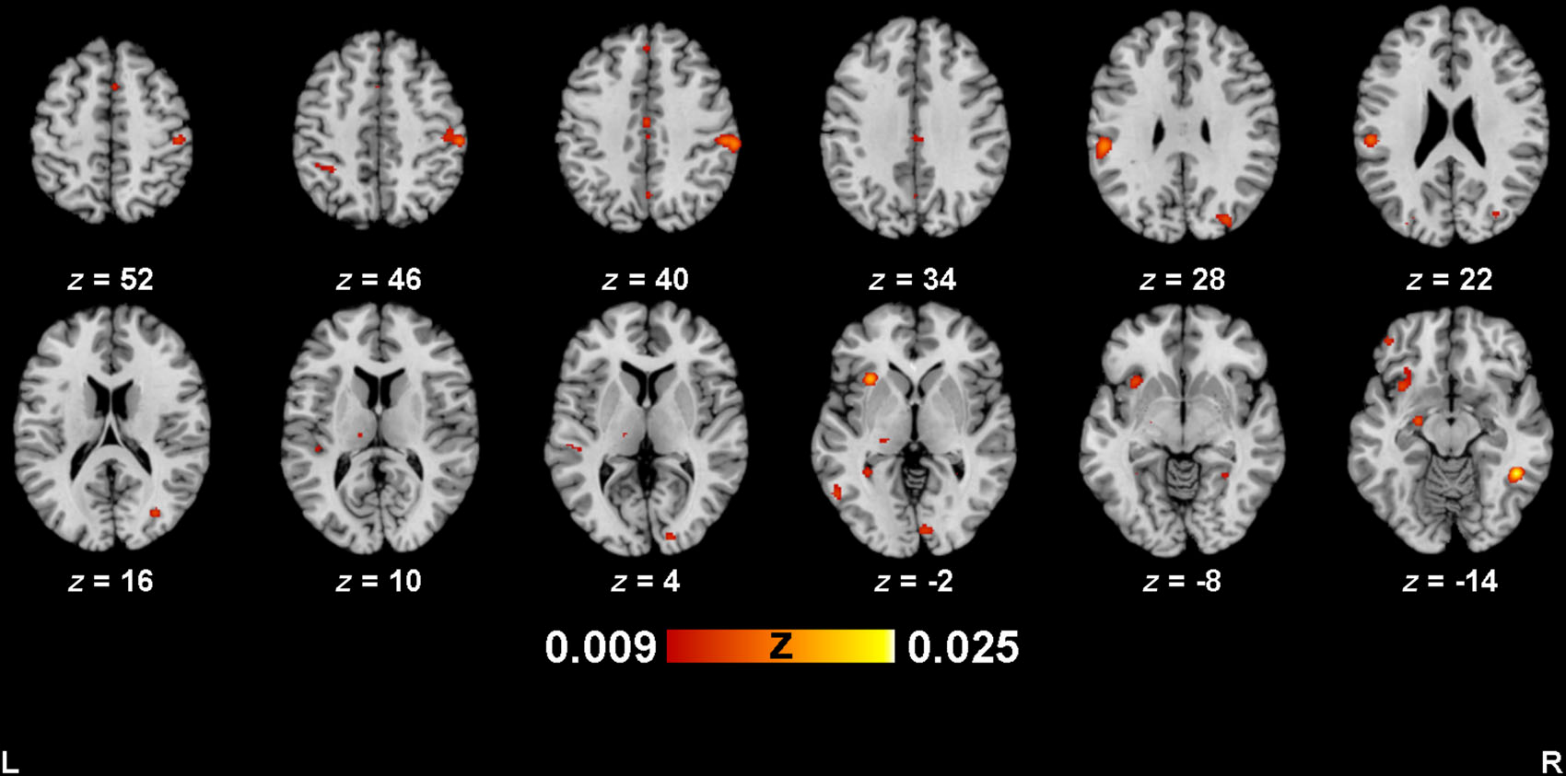
Activation map for empathy processing task, IG>OG. Activation maps were thresholded at *p *< 0.005 (uncorrected), with a minimum cluster size of 200 mm^3^. Images are in neurological convention.

Included studies had a median sample size of 20.5 (range: 10–58). Across all studies (*n* = 30), there were 50 experiments (880 subjects, 260 foci, 3 out‐of‐mask foci) for bias during empathy processing tasks toward in‐group (IG>OG) analysis. Analysis for IG>OG revealed 23 significant clusters (Table 4; Figure 4), with the largest (2480 mm^3^) located in the right postcentral gyrus, and right posterior cingulate gyrus. Other clusters included the left inferior parietal lobule, left IFG, right dorsal lateral occipital complex, right fusiform gyrus, and left amygdala.

**TABLE 4:** ALE results of in‐group bias in Empathy Processing.

Cluster	Volume (mm^3^)	Max ALE (x10^−2^)	MNI Coordinates			Label
			*x*	*y*	*z*	
1	2480	1.79	58	−26	44	Right postcentral gyrus
		1.32	48	−26	52	Right posterior cingulate gyrus
2	1656	1.74	−54	−30	28	Left inferior parietal lobule
		1.65	−56	−26	24	Left Inferior parietal lobule
3	1648	2.13	−32	18	−4	Left inferior frontal gyrus
		1.50	−34	14	−12	Left insula
		1.06	−30	24	−14	Left inferior frontal gyrus
4	968	1.47	32	−76	18	Right dorsal lateral occipital complex
		1.37	32	−82	28	Right middle occipital gyrus
5	960	2.50	46	−48	−14	Right fusiform gyrus
6	624	1.86	−32	−6	−24	Left amygdala
7	512	1.19	−32	−46	44	Left inferior parietal lobule
		1.17	−42	−42	44	Left inferior parietal lobule
8	480	1.45	−22	−12	−14	Left amygdala
9	456	1.27	8	−88	−2	Right lingual gyrus
10	440	1.24	−22	−26	0	Left thalamus
		1.11	−14	−20	8	Left thalamus
11	400	1.32	−54	−62	−2	Left middle occipital gyrus
12	400	1.19	0	−24	36	Left cingulate gyrus
		1.01	6	−24	32	Right cingulate gyrus
13	360	1.04	−48	−30	6	Left superior temporal gyrus
		1.04	−44	−30	8	Left superior temporal gyrus
14	344	1.37	−32	−48	−4	Left parahippocampal gyrus
15	304	1.75	22	−4	66	Right superior frontal gyrus
16	288	1.32	16	−92	6	Right lingual gyrus
17	256	1.33	−42	44	−16	Left middle frontal gyrus
18	256	1.30	0	−12	40	Left cingulate gyrus
19	240	1.27	30	−48	−6	Right parahippocampal gyrus
20	240	1.17	2	12	52	Left superior frontal gyrus
		0.94	−2	12	44	Left medial frontal gyrus
21	216	1.08	−26	−80	20	Left middle occipital gyrus
22	216	1.17	0	38	42	Left superior frontal gyrus
23	208	1.27	2	−64	38	Left precuneus

*Note*: All clusters reported survived a voxel‐level uncorrected threshold of *p* < 0.005, with a minimum cluster size of 200 mm^3^. Coordinates are reported in the Montreal Neurological Institute (MNI) convention.

Abbreviation: ALE: activation likelihood estimate.

## 4 Discussion

The present meta‐analysis consolidated findings from extant fMRI studies investigating group categorization, elucidating the neural substrates showing convergent activation during in‐group and out‐group processing. In addition, it differentiated patterns based on varying group memberships and fMRI task types, specifically on the ethnicity group and empathy processing task respectively. Our results showing the overall activation patterns were consistent with findings from previous meta‐analyses for neural substrates of in‐group and out‐group processing, and ethnicity group membership and empathy processing fMRI task employed (Molenberghs and Louis 2018; Saarinen et al. 2021; Saarinen et al. 2023).

### 4.1 Neural Substrates of In‐Group Processing

Overall IG>OG processing regardless of group membership and task category revealed convergent activation in left insula and IFG, both of which are activated when the self is involved (Northoff and Bermpohl 2004). This finding is in line with the idea that the in‐group is closely related to the self (Smith and Henry 2016). Our meta‐analysis showed more recruitment of self‐related areas in in‐group bias as expected.

The left insula showed consistent activation across studies examining in‐group processing. Previous literature has reported left insula activation during empathy and emotion recognition tasks (e.g., Amodio 2014), and our finding of convergent activation in this region during in‐group processing (Bagnis et al. 2020) may reflect this process. Of relevance, 29 out of 64 number of studies exploring IG>OG assessed judgments to empathy processing task. Given that left insula activation occurs during various social bonding paradigms (Northoff et al. 2011), a process essential for navigating social relationships, its convergent activation in our meta‐analysis may reflect emotional engagement when processing in‐group stimuli.

Moreover, IFG, particularly its left hemisphere, showed convergent activation during in‐group processing across studies. Research across various studies has identified the IFG as a region that exhibits increased activation during the processing of in‐group stimuli (Scheepers et al. 2013), as it may be engaged in the recognition and categorization of individuals belonging to the same social group (Uddin et al. 2005). Similar to the left insula, the IFG's involvement in in‐group processing also extends to emotional and affective components of social interactions (Taylor et al. 2009). This emotional dimension may involve the generation of positive affect, attachment, or a sense of belonging when individuals process stimuli related to their own social group. Moreover, the IFG's role in in‐group processing may have implications for perspective‐taking and theory of mind (Amodio and Frith 2006; Siegal and Varley 2002), a crucial aspect of navigating social relationships within the in‐group context. Our finding of convergent IFG activation during in‐group processing may reflect these cognitive operations.

Interestingly, the right fusiform gyrus showed significant convergence, potentially reflecting that 15 of 66 studies employed face processing tasks. The fusiform gyrus plays a crucial role in the neural mechanisms associated with the recognition and processing of faces belonging to one's in‐group (Van Bavel et al. 2011), particular for faces of familiar in‐group members (Gauthier et al. 1999; Golby et al. 2001). Convergent activation in this region during IG>OG contrasts may reflect enhanced visual processing of in‐group faces, though this region also activates during expert object recognition (Kanwisher et al. 1997; Rhodes et al. 2004).

**FIGURE 5:**
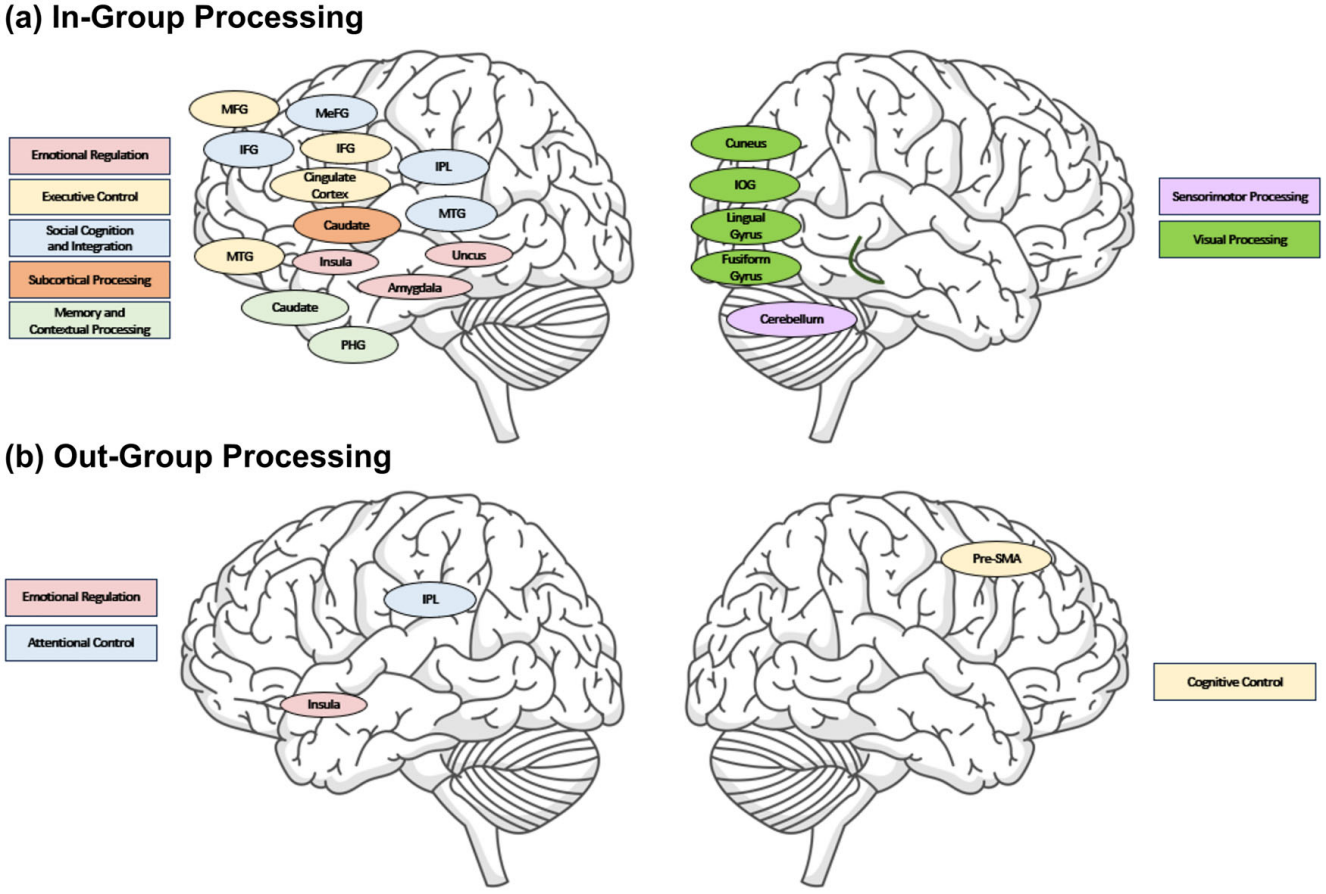
Illustration of observed (a) neural substrates for in‐group processing and (b) neural substrates for out‐group processing. ACC = anterior cingulate cortex; IFG = inferior frontal gyrus; IOG = inferior occipital gyrus; IPL = inferior parietal lobule; MeFG = medial frontal gyrus; MFG = medial frontal gyrus; MTG = middle temporal gyrus; PCG = precentral gyrus; PHG = parahippocampal gyrus; Pre‐SMA = presupplementary motor area. Outline of the brain was accessed for free on Freepik (https://www.freepik.com/free‐psd/brain‐outline‐illustration_65105163.htm).

Taken together, these regions collectively constitute the neural substrates showing convergent activation during in‐group processing (Figure 5a). The observed convergence spans regions implicated in (1) emotional processing (left amygdala, bilateral insula, left uncus); (2) visual processing (bilateral fusiform gyrus, bilateral lingual gyrus, bilateral inferior occipital gyrus, right cuneus); (3) executive control (bilateral IFG, bilateral medial frontal gyrus, left middle frontal gyrus, bilateral cingulate cortex); (4) social cognition and integration (bilateral inferior parietal lobule, bilateral middle temporal gyrus, left inferior temporal gyrus); (5) memory and contextual processing (bilateral parahippocampal gyrus, left caudate); (6) sensorimotor processing (right cerebellum); and 7) subcortical processing (left thalamus, left caudate). The integration of these regions forms a set of neural substrates that operate synergistically during in‐group processing, collectively contributing to the multifaceted nature of in‐group bias.

### 4.2 Neural Substrates of Out‐Group Processing

Overall OG>IG processing revealed convergent activation in the right presupplementary motor area, a region showing increased activation during cognitive control tasks (Hertrich 2016). This suggests that in‐group processing may be more automatic whereas more cognitive resources are required for out‐group processing, as expected (Zhu et al. 2023).

For instance, when encountering out‐group members, individuals may need to inhibit or override automatic stereotypes and biases that can influence perception. Overcoming these stereotypes demands additional cognitive effort, and may necessitate heightened attentional control to process information from less familiar or socially distant sources. This is also in line with the convergent activation in inferior parietal lobule, which shows increased activity during attention‐demanding tasks (Bagnis et al. 2020). Furthermore, integrating information about out‐group members may require greater cognitive flexibility, which demands additional processing resources to adapt to novel information. Finally, interactions with out‐group members may evoke emotions related to intergroup dynamics, such as anxiety, uncertainty, or apprehension (Stephan 2014). The finding of the activity of the bilateral insula is significant, given that the insula is strongly associated with the processing of emotional stimuli, including the regulation of emotional responses (Taylor et al. 2009).

In summary, the neural substrates showing convergent activation during out‐group processing span regions implicated in cognitive control, attention, and emotional regulation Figure 5b). While this pattern may reflect increased cognitive demands during out‐group processing, alternative interpretations including task difficulty or stimulus novelty cannot be excluded. The observed convergence aligns with behavioral evidence suggesting greater cognitive effort during out‐group interactions (Stephan 2014).

### 4.3 Ethnicity In‐Group Bias

Occipital regions showed convergent activation for ethnicity group processing, specifically in‐group bias (IG>OG), consistent with a review (Bagnis et al. 2020) reporting recruitment of these regions for racial traits perception. As occipital regions are linked with facial representation and encoding of invariant visual traits like ethnicity, the observed convergence could imply greater focus on individual distinctions within the in‐group.

Right middle temporal gyrus convergence may reflect its engagement in social categorization processes (Katsumi and Dolcos 2018), which involve classification of individuals into social groups, though this region is also associated with social identity processing and affiliation (Scheepers et al. 2013), and therefore may play a role in the neural processes that underlie a sense of belonging and affiliation with individuals of the same ethnic background. Convergence in right fusiform gyrus may potentially reflect that 12 out of 38 number of studies examining ethnicity in‐group bias employed face processing task. Previous studies report the fusiform gyrus, particularly in the right hemisphere, has been implicated in the activation of implicit biases and stereotypes (Brosch et al. 2013). In the case of ethnic in‐group bias, the right fusiform gyrus may be involved in the rapid and automatic activation of stereotypes associated with one's own ethnic group. Therefore, convergence in the right fusiform gyrus might be associated with in‐group favoritism (Van Bavel et al. 2008), contributing to more positive evaluations and perceptions of individuals belonging to one's own ethnic group.

### 4.4 In‐Group Bias in Empathy Processing Task

Postcentral gyrus and posterior cingulate showed convergent activation during empathy processing tasks with IG>OG contrasts. These areas have been associated with the sensory and affective facets of empathy, respectively (Singer et al. 2004; Völlm et al. 2006). This stronger empathic response appears evident for the in‐group than the out‐group as expected. This stronger empathic response is rooted in phenomena such as the in‐group bias, which extends to empathy, with people showing a greater emotional response and understanding toward the experiences of in‐group members. Furthermore, individuals typically share more familiarity and similarities with members of their in‐group, thereby fostering a sense of connection and understanding, aiding in empathy with experiences and emotions of the in‐group.

Additional convergent activation in left IFG, which activates during perspective‐taking tasks (Molenberghs et al. 2016), and left amygdala, which shows increased activity during emotional processing (Clark et al. 2018), suggests multiple neural substrates are consistently engaged during empathy tasks involving group distinctions.

### 4.5 Limitations and Future Directions

While this meta‐analysis highlights consistent regions of activity for the IG>OG, and OG>IG, as well as distinctions made for in‐group bias in the ethnicity group and Empathy Processing task, the current study is not without limitations.

While a large number of studies were included, the generalizability of the findings was limited by several factors, including the participant demographics in the individual studies. The predominant recruitment of participants from the United States (*n* = 38) limited the extensiveness of our observations, precluding comprehensive insights into cultural variations that may engender distinct social cognitive processes (Vogeley and Roepstorff 2009). In addition, an overwhelming focus on in‐group bias (IG>OG) in the majority of the included studies, as opposed to a comparatively scant exploration of responses to OG>IG, introduced an asymmetry in the number of studies and foci available for each type of comparison, hindering direct comparisons between these contrasting conditions. Notably, ethnicity group (*n* = 49) emerged as the most frequently studied group membership, potentially reflecting its visual saliency compared to less conspicuous categories such as nationality. In addition, empathy processing (*n* = 38) took precedence among distinct task types, likely owing to its intrinsic association with intergroup biases. However, the preponderance of studies focusing on ethnicity and empathy processing introduced the potential for biased outcomes, emphasizing the need for a more balanced exploration across diverse group memberships and task types in future investigations.

Furthermore, in the present meta‐analysis, the consideration of minority versus majority status within individual studies was not explicitly incorporated. This decision was made considering the contextual variability across studies, where each investigation might define minority and majority groups based on the specific sociodemographic composition of the population under study. The importance of considering the minority versus majority status has been highlighted in a recent meta‐analysis by Saarinen et al. (2023), which indicated an absence of neural intergroup bias in minority members. However, the overarching focus of the present meta‐analysis was on the general patterns of in‐group and out‐group processing, examining the overall neural activity associated with processing one group relative to another. This approach was chosen to capture the broader dynamics of intergroup processing. Despite so, it is acknowledged that when delving into analyses involving specific minority and majority distinctions, particularly across diverse tasks, the influence of minority versus majority status may become more pronounced. Future meta‐analyses may consider these distinctions to offer a more granular understanding of the neural mechanisms underlying intergroup processing.

Moreover, whilst we implemented a quality scoring system for included studies, this scale has not been formally validated for neuroimaging meta‐analyses. In addition, we employed an uncorrected statistical threshold (*p* < 0.005), which may increase false positive risk. FWE‐corrected analyses (Supporting Information S2) confirmed that core regions remained significant, though the number of significant clusters was reduced. The heterogeneity in analysis software and statistical thresholds across included studies represents an inherent limitation of coordinate‐based meta‐analyses, as the ALE algorithm treats all reported foci as equally reliable regardless of their original statistical threshold (Eickhoff et al. 2012). Future meta‐analyses may benefit from weighted approaches that account for study‐specific factors such as sample size and statistical stringency.

In addition, we acknowledge the reverse inference problem (Poldrack 2006; Poldrack and Yarkoni 2016) inherent in inferring cognitive processes from activation data. Our findings describe neural substrates consistently activated during specific task contrasts rather than definitive neural mechanisms of cognitive processes. The convergence of activation does not establish that these regions are specific to, necessary for, or sufficient for group categorization processes. Interpretations of cognitive functions from regional activation remain probabilistic, as identified regions likely support multiple cognitive operations beyond those examined here. Furthermore, coordinate‐based meta‐analyses may not be able to differentiate the implication of group‐specific processing, task‐specific demands, or their interaction, solely from observed convergence patterns. Whether the distinct neural substrates identified for ethnicity‐based categorization and empathy processing represent fundamentally different neural mechanisms or variations in cognitive demands remains a question that requires experimental manipulation beyond meta‐analytic approaches.

Finally, the ALE methodology identifies convergent activation through coordinate‐based meta‐analysis rather than establishing functional or effective connectivity between regions. This approach therefore cannot determine whether the identified regions interact directly or represent parallel processing streams, nor can it establish temporal dynamics or causal relationships (Eickhoff et al. 2009). Future investigations employing connectivity‐based approaches could complement the findings by examining the functional network among the identified neural substrates during group categorization.

## 5 Conclusion

The present meta‐analysis advances beyond previous work by combining an unrestricted search approach and systematic differentiation of group types and tasks with an ALE framework. Previous meta‐analyses employed predetermined category searches that constrained their scope to specific group types. Our broader approach, which did not limit searches to predefined categories, captures a more comprehensive range of group dynamics. We demonstrate that different group memberships and task contexts engage distinct neural substrates rather than a universal system. The emergence of unique activation patterns for in‐group ethnicity‐based categorization and empathy processing tasks suggests the need to expand existing models of intergroup bias. These models have typically assumed more universal mechanisms, yet our findings indicate that the brain processes group categorization through multiple pathways dependent on specific group characteristics and task demands. Interventions targeting intergroup bias require consideration of these context‐specific neural pathways rather than assuming uniform mechanisms across all forms of group categorization.

## Author Contributions

**Kelly H.L. Sng**: conceptualization, methodology, formal analysis, investigation, data curation, writing – original draft, visualization. **Xavier Y.H. Lim**: investigation, data curation, writing – review and editing, visualization. **Annabel S.H. Chen**: writing – review and editing, supervision, funding acquisition. **Gianluca Esposito**: conceptualization, writing – review and editing, supervision.

## Funding

This work is supported by Temasek Laboratories, Singapore.

## Ethics Statement

The authors have nothing to report.

## Conflicts of Interest

The authors declare no conflicts of interest.

## Supporting information




**Supplementary Materials**: brb371314‐sup‐0001‐SuppMat.docx

**Supplementary Materials**: brb371314‐sup‐0002‐SuppMat.docx

## Data Availability

All data were extracted from previously published data.
