# Supplementary material for: A Meta‐Analysis of Functional Magnetic Resonance Imaging Studies on In‐Group and Out‐Group Categorization
Source: Brain Behav. 2026 Mar 31;16(4):e71314. doi: 10.1002/brb3.71314 (PMC13112008; doi:10.1002/brb3.71314)
Supplement: Supplementary file 1 — Supplementary Materials: brb371314‐sup‐0001‐SuppMat.docx [file BRB3-16-e71314-s001.docx]

# Table S1.1

*Articles included and excluded based on systematic literature search via databases*

| **Author(s)** | **Year** | **Journal** | **Included/ Excluded** | **Reason for Exclusion** |
| --- | --- | --- | --- | --- |
| Adams, R. B. et al. | 2010 | Journal of Cognitive Neuroscience | Included |  |
| Azevedo, R. T. et al. | 2013 | Human Brain Mapping | Included |  |
| Baumgartner, T. et al. | 2012 | Human Brain Mapping | Included |  |
| Berlingeri, M. et al. | 2016 | Neuroscience | Included |  |
| Bestelmeyer, P. E. et al. | 2015 | Cerebral Cortex | Included |  |
| Brown, T. I. et al. | 2017 | PLoS ONE | Included |  |
| Bruneau, E. G. & Saxe, R. | 2010 | NeuroImage | Included |  |
| Bruneau, E. G. et al. | 2012 | Philosophical Transactions of the Royal Society B: Biological Sciences | Included |  |
| Cao, Y. et al. | 2015 | Cortex | Included |  |
| Carollo, A. et al. | 2023 | Sensors | Included |  |
| Cassidy, B. S. & Krendl, A. C. | 2016 | NeuroImage | Included |  |
| Cassidy, B. S. et al. | 2021 | Aging, Neuropsychology, and Cognition | Included |  |
| Chen, P. A. et al. | 2015 | Social Psychological and Personality Science | Included |  |
| Cheon, B. K. et al. | 2011 | NeuroImage | Included |  |
| Contreras-Huerta, L. S. et al. | 2013 | PLoS ONE | Included |  |
| Cui, F. et al. | 2023 | British Journal of Psychology | Included |  |
| Domínguez D, J. F. et al. | 2018 | Social Neuroscience | Included |  |
| Ebner, N. C. et al. | 2013 | NeuroImage | Included |  |
| Fang, Z. et al. | 2024 | Behavioural Brain Research | Included |  |
| Farmer, H. et al. | 2020 | Scientific Reports | Included |  |
| Feng, C. et al. | 2017 | Human Brain Mapping | Included |  |
| Feng, L. et al. | 2011 | Neuropsychologia | Included |  |
| Firat, R. B. et al. | 2017 | Social Cognitive and Affective Neuroscience | Included |  |
| Fourie, M. M. et al. | 2017 | Social Cognitive and Affective Neuroscience | Included |  |
| Fox, G. R. et al. | 2013 | Frontiers in Psychology | Included |  |
| Freeman, J. B. et al. | 2010 | Human Brain Mapping | Included |  |
| Gilbert, S. J. et al. | 2012 | Neuropsychologia | Included |  |
| Handley, G. et al. | 2023 | NeuroImage | Included |  |
| Hein, G. et al. | 2016 | Proceedings of the National Academy of Sciences of the United States of America | Included |  |
| Hein, G. et al. | 2010 | Neuron | Included |  |
| Izuma, K. et al. | 2019 | NeuroImage | Included |  |
| Jiang, X. et al. | 2018 | NeuroImage | Included |  |
| Junger, J. et al. | 2013 | NeuroImage | Included |  |
| Kang, P. et al. | 2021 | Journal of Neuroscience | Included |  |
| Katsumi, Y. & Dolcos, S. | 2018 | Frontiers in Human Neuroscience | Included |  |
| Kim, K. & Johnson, M. K. | 2015 | Social Neuroscience | Included |  |
| Krendl, A. C. et al. | 2009 | Psychology and Aging | Included |  |
| Krosch, A. R. & Amodio, D. M. | 2019 | Journal of Personality and Social Psychology | Included |  |
| Lasko, E. N. et al. | 2022 | Social Neuroscience | Included |  |
| Lau, T. & Cikara, M. | 2017 | Scientific Reports | Included |  |
| Lee, K. U. et al. | 2008 | NeuroReport | Included |  |
| Li, X. et al. | 2015 | NeuroImage | Included |  |
| Li, Z. et al. | 2020 | NeuroImage | Included |  |
| Lin, L. C. et al. | 2018 | Proceedings of the National Academy of Sciences of the United States of America | Included |  |
| Littlefield, M. M. et al. | 2015 | Frontiers in Human Neuroscience | Included |  |
| Losin, E. A. R. et al. | 2012 | NeuroImage | Included |  |
| Losin, E. A., et al. | 2012 | Social Cognitive and Affective Neuroscience | Included |  |
| Luo, S. et al. | 2015 | NeuroImage | Included |  |
| Marsh, L. E. et al. | 2016 | NeuroImage | Included |  |
| Mathur, V. A. et al. | 2012 | Human Brain Mapping | Included |  |
| Mathur, V. A. et al. | 2010 | NeuroImage | Included |  |
| Mattan, B. D. et al. | 2018 | Social Cognitive and Affective Neuroscience | Included |  |
| Mauchaund, M. et al. | 2023 | Social Cognitive Affective Neuroscience | Included |  |
| McCutcheon, R. et al. | 2018 | Psychological Medicine | Included |  |
| Mei, S. et al. | 2025 | Science Advances | Included |  |
| Molenberghs, P. & Morrison, S. | 2014 | Social Cognitive and Affective Neuroscience | Included |  |
| Molenberghs, P. et al. | 2017 | Journal of Management | Included |  |
| Molenberghs, P. et al. | 2016 | Cerebral Cortex | Included |  |
| Molenberghs, P. et al. | 2013 | Human Brain Mapping | Included |  |
| Molenberghs, P., Bosworth, R. et al. | 2014 | Human Brain Mapping | Included |  |
| Moradi, Z. et al. | 2017 | Behavioural Brain Research | Included |  |
| Morrison, S. et al. | 2012 | Neuropsychologia | Included |  |
| Nugiel, T. & Beer, J. S. | 2020 | Journal of Cognitive Neuroscience | Included |  |
| Raghunath, B. L. et al. | 2022 | Scientific Reports | Included |  |
| Rauchbauer, B. et al. | 2015 | Cortex | Included |  |
| Richeson, J. A. et al. | 2003 | Nature Neuroscience | Included |  |
| Richins, M. T. et al. | 2019 | Social Neuroscience | Included |  |
| Ronquillo, J. et al. | 2007 | Social Cognitive and Affective Neuroscience | Included |  |
| Rubien-Thomas, E. et al. | 2021 | Cognitive, Affective and Behavioral Neuroscience | Included |  |
| Ruckmann, J. et al. | 2015 | Psychiatry Research - Neuroimaging | Included |  |
| Rule, N. O. et al. | 2010 | Social Cognitive and Affective Neuroscience | Included |  |
| Scheepers, D. et al. | 2013 | Frontiers in Human Neuroscience | Included |  |
| Sheng, F. et al. | 2014 | NeuroImage | Included |  |
| Steines, M. et al. | 2020 | Cortex | Included |  |
| Telzer, E. H. et al. | 2015 | NeuroImage | Included |  |
| van Gils, S. et al. | 2020 | Journal of Neuroscience, Psychology, and Economics | Included |  |
| Volz, K. G. et al. | 2009 | Social Neuroscience | Included |  |
| Wang, C. et al. | 2015 | Social Cognitive and Affective Neuroscience | Included |  |
| Wu, C. T. et al. | 2018 | Frontiers in Human Neuroscience | Included |  |
| Yan, Z. et al. | 2019 | Social Cognitive and Affective Neuroscience | Included |  |
| Zacharopoulos, G. et al. | 2023 | Social Neuroscience | Included |  |
| Kluge, A. & Levy, J. | 2025 | Neuropsychologia | Excluded | Not task-based fMRI |
| Kluge, A. et al. | 2024 | Frontiers in Social Psychology | Excluded | Not task-based fMRI |
| Kotikalapudi, R. et al. | 2023 | Scientific Reports | Excluded | Not task-based fMRI |
| Silvestri, V. et al. | 2022 | PLoS ONE | Excluded | Not task-based fMRI |
| Yang, J. et al. | 2020 | Nature Neuroscience | Excluded | Not task-based fMRI |
| Huang, S. & Han, S. | 2014 | Social Neuroscience | Excluded | Not task-based fMRI |
| Cunningham, W. A. et al. | 2012 | Frontiers in Human Neuroscience | Excluded | Not task-based fMRI |
| Stanley, D. A. et al. | 2011 | Proceedings of the National Academy of Sciences of the United States of America | Excluded | Not task-based fMRI |
| Kasparek, S. W. et al. | 2023 | Research on Child and Adolescent Psychopathology | Excluded | Not task-based fMRI |
| Manfredi, M. et al. | 2023 | BMC Psychology | Excluded | Not task-based fMRI |
| Neoh, M. J. Y. et al. | 2023 | Brain Sciences | Excluded | Not task-based fMRI |
| Zebarjadi, N. et al. | 2023 | Social Cognitive and Affective Neuroscience | Excluded | Not task-based fMRI |
| Brandner, P. et al. | 2021 | Developmental Cognitive Neuroscience | Excluded | Not neurotypicals |
| Telzer, E. H. et al. | 2020 | Dev Psychopathol | Excluded | Not neurotypicals |
| Fourie, M. M. et al. | 2019 | Scientific Reports | Excluded | Not neurotypicals |
| Do, K. T. & Telzer, E. H. | 2019 | Developmental Cognitive Neuroscience | Excluded | Not neurotypicals |
| Sankar, A. et al. | 2018 | Neuroscience Letters | Excluded | Not neurotypicals |
| Guassi Moreira, J. F. et al. | 2017 | Social Cognitive and Affective Neuroscience | Excluded | Not neurotypicals |
| Bolling, D. Z. et al. | 2016 | Social Neuroscience | Excluded | Not neurotypicals |
| Blackford, J. U. et al. | 2015 | Schizophrenia Research | Excluded | Not neurotypicals |
| Telzer, E. H. et al. | 2013 | Journal of Cognitive Neuroscience | Excluded | Not neurotypicals |
| Welte, M. et al. | 2025 | Psychological Review | Excluded | Review study |
| Zhu, J. & Zhou, Y. | 2025 | Chinese Science Bulletin | Excluded | Review study |
| Forbes, C. E. | 2024 | Cortex | Excluded | Review study |
| Peng, K. et al. | 2024 | Journal of Integrative Neuroscience | Excluded | Review study |
| Saarinen, A. et al. | 2023 | Frontiers in Human Neuroscience | Excluded | Review study |
| Uddin, L. Q. & De Los Reyes, A. | 2022 | Biological Psychiatry: Cognitive Neuroscience and Neuroimaging | Excluded | Review study |
| Grasser, L. R. & Jovanovic, T. | 2022 | Biological Psychiatry: Cognitive Neuroscience and Neuroimaging | Excluded | Review study |
| Ficco, L. et al. | 2022 | British Journal of Psychology | Excluded | Review study |
| Saarinen, A. et al. | 2021 | Neuroscience and Biobehavioral Reviews | Excluded | Review study |
| Merritt, C. C. et al. | 2021 | Social Cognitive and Affective Neuroscience | Excluded | Review study |
| Lantos, D. & Molenberghs, P. | 2021 | Neuroscience and Biobehavioral Reviews | Excluded | Review study |
| Amodio, D. M. & Cikara, M. | 2021 | Annual Review of Psychology | Excluded | Review study |
| Bagnis, A. et al. | 2020 | NeuroImage | Excluded | Review study |
| Molenberghs, P. & Louis, W. R. | 2018 | Frontiers in Psychology | Excluded | Review study |
| Han, S. | 2018 | Trends in Cognitive Sciences | Excluded | Review study |
| Scheepers, D. & Derks, B. | 2016 | Current Opinion in Psychology | Excluded | Review study |
| Halevy, N. et al. | 2015 | Current Opinion in Psychology | Excluded | Review study |
| Cikara, M. | 2015 | Current Opinion in Behavioral Sciences | Excluded | Review study |
| Chekroud, A. M. & | 2014 | Frontiers in Human Neuroscience | Excluded | Review study |
| Shkurko, A. V. | 2013 | Social Cognitive and Affective Neuroscience | Excluded | Review study |
| Molenberghs, P. | 2013 | Neuroscience and Biobehavioral Reviews | Excluded | Review study |
| Powers, K. E. & Heatherton, T. F. | 2012 | Frontiers in Integrative Neuroscience | Excluded | Review study |
| Ito, T. A. & Bartholow, B. D. | 2009 | Trends in Cognitive Sciences | Excluded | Review study |
| Derks, B. et al. | 2008 | Group Processes and Intergroup Relations | Excluded | Review study |
| Phelps, E. A. & Thomas, L. A. | 2003 | Political Psychology | Excluded | Review study |
| Ficco, L. et al. | 2023 | British Journal of Psychology | Excluded | Review study |
| Liu, J. et al. | 2014 | Cerebral Cortex | Excluded | Case study |
| Rubien-Thomas, E. et al. | 2023 | Cognitive, Affective and Behavioral Neuroscience | Excluded | No whole-brain coordinates |
| Zhou, Y. et al. | 2022 | Journal of Neuroscience | Excluded | No whole-brain coordinates |
| Liu, Y. et al. | 2022 | Cerebral Cortex | Excluded | No whole-brain coordinates |
| Hanley, C. J. et al. | 2022 | Behavioural Brain Research | Excluded | No whole-brain coordinates |
| Cassidy, B. S. et al. | 2021 | Aging, Neuropsychology, and Cognition | Excluded | No whole-brain coordinates |
| Reggev, N. et al. | 2020 | eNeuro | Excluded | No whole-brain coordinates |
| Harada, T. et al. | 2020 | Neuropsychologia | Excluded | No whole-brain coordinates |
| Han, X. et al. | 2020 | eLife | Excluded | No whole-brain coordinates |
| Hughes, B. L. et al. | 2019 | Proceedings of the National Academy of Sciences of the United States of America | Excluded | No whole-brain coordinates |
| Hein, G. et al. | 2018 | Proceedings of the Royal Society B: Biological Sciences | Excluded | No whole-brain coordinates |
| Hackel, L. M. et al. | 2017 | Social Cognitive and Affective Neuroscience | Excluded | No whole-brain coordinates |
| Haas, I. J. et al. | 2017 | Social Justice Research | Excluded | No whole-brain coordinates |
| Greven, I. M. & Ramsey, R. | 2017 | Neuropsychologia | Excluded | No whole-brain coordinates |
| Powers, K. E. et al. | 2016 | Journal of Cognitive Neuroscience | Excluded | No whole-brain coordinates |
| Stallen, M. et al. | 2013 | Frontiers in Human Neuroscience | Excluded | No whole-brain coordinates |
| Ratner, K. G. et al. | 2013 | Social Cognitive and Affective Neuroscience | Excluded | No whole-brain coordinates |
| Brosch, T. et al. | 2013 | Psychological Science | Excluded | No whole-brain coordinates |
| van Bavel, J. J. et al. | 2011 | Journal of Cognitive Neuroscience | Excluded | No whole-brain coordinates |
| Krill, A. & Platek, S. M. | 2009 | Frontiers in Evolutionary Neuroscience | Excluded | No whole-brain coordinates |
| Derntl, B. et al. | 2009 | BMC Neuroscience | Excluded | No whole-brain coordinates |
| Hart, A. J. et al. | 2000 | NeuroReport | Excluded | No whole-brain coordinates |
| Duan, Q. et al. | 2024 | Neuropharmacology | Excluded | Unclear/ unspecified in-group/ out-group |
| Gonzalez, F. J. & Haas, I. J. | 2024 | Journal of Experimental Political Science | Excluded | Unclear/ unspecified in-group/ out-group |
| Jacoby, N. et al. | 2024 | Cerebral Cortex | Excluded | Unclear/ unspecified in-group/ out-group |
| Lois, G. et al. | 2024 | Social Cognitive and Affective Neuroscience | Excluded | Unclear/ unspecified in-group/ out-group |
| Hanley, C. J. et al. | 2023 | Neurobiological of Aging | Excluded | Unclear/ unspecified in-group/ out-group |
| Welborn, B. L. et al. | 2023 | Social Cognitive and Affective Neuroscience | Excluded | Unclear/ unspecified in-group/ out-group |
| Fornari, L. et al. | 2023 | Nature Communications | Excluded | Unclear/ unspecified in-group/ out-group |
| Vijayakumar, S. et al. | 2021 | Social Cognitive and Affective Neuroscience | Excluded | Unclear/ unspecified in-group/ out-group |
| Krosch, A. R. et al. | 2021 | Philosophical Transactions of the Royal Society B: Biological Sciences | Excluded | Unclear/ unspecified in-group/ out-group |
| Shin, W. G. et al. | 2020 | Frontiers in Behavioral Neuroscience | Excluded | Unclear/ unspecified in-group/ out-group |
| Park, B. & Young, L. | 2020 | Journal of Experimental Social Psychology | Excluded | Unclear/ unspecified in-group/ out-group |
| Lelieveld, G. J. et al. | 2020 | Social Cognitive and Affective Neuroscience | Excluded | Unclear/ unspecified in-group/ out-group |
| Lau, T. et al. | 2020 | Elife | Excluded | Unclear/ unspecified in-group/ out-group |
| Lantos, D. et al. | 2020 | Social Neuroscience | Excluded | Unclear/ unspecified in-group/ out-group |
| Kesner, L. et al. | 2020 | Frontiers in Behavioral Neuroscience | Excluded | Unclear/ unspecified in-group/ out-group |
| Dricu, M. et al. | 2020 | Scientific Reports | Excluded | Unclear/ unspecified in-group/ out-group |
| Hughes, C. et al. | 2019 | Frontiers in Human Neuroscience | Excluded | Unclear/ unspecified in-group/ out-group |
| Andrews, T. J. et al. | 2019 | Cerebral Cortex | Excluded | Unclear/ unspecified in-group/ out-group |
| Carpenter, A. C. & Krendl, A. C. | 2018 | Social Neuroscience | Excluded | Unclear/ unspecified in-group/ out-group |
| Spiers, H. J. et al. | 2017 | Journal of Cognitive Neuroscience | Excluded | Unclear/ unspecified in-group/ out-group |
| Krendl, A. C. & Kensinger, E. A. | 2016 | PLoS ONE | Excluded | Unclear/ unspecified in-group/ out-group |
| Dunsmoor, J. E. et al. | 2016 | Social Cognitive and Affective Neuroscience | Excluded | Unclear/ unspecified in-group/ out-group |
| Senholzi, K. B. et al. | 2015 | Social Neuroscience | Excluded | Unclear/ unspecified in-group/ out-group |
| Fourie, M. M. et al. | 2014 | Social Neuroscience | Excluded | Unclear/ unspecified in-group/ out-group |
| Stanley, D. A. et al. | 2012 | Philosophical Transactions of the Royal Society B: Biological Sciences | Excluded | Unclear/ unspecified in-group/ out-group |
| Masten, C. L. et al. | 2011 | Journal of Cognitive Neuroscience | Excluded | Unclear/ unspecified in-group/ out-group |
| Cikara, M. et al. | 2010 | Social Cognitive and Affective Neuroscience | Excluded | Unclear/ unspecified in-group/ out-group |
| Krendl, A. C. et al. | 2006 | Social Neuroscience | Excluded | Unclear/ unspecified in-group/ out-group |
| Stolier, R. M. and Freeman, J. B. | 2017 | Journal of Neuroscience | Excluded | No suitable contrasts |
| Reimers, L. et al. | 2017 | NeuroImage | Excluded | No suitable contrasts |
| Gamond, L. et al. | 2017 | European Journal of Neuroscience | Excluded | No suitable contrasts |
| Terbeck, S. et al. | 2015 | Psychopharmacology | Excluded | No suitable contrasts |
| Powers, K. E. & Heatherton, T. F. | 2013 | PLoS ONE | Excluded | No suitable contrasts |
| Cheon, B. K. et al. | 2013 | Neuropsychologia | Excluded | No suitable contrasts |
| Sobhani, M. et al. | 2012 | PLoS ONE | Excluded | No suitable contrasts |
| Korn, H. A. et al. | 2012 | Social Neuroscience | Excluded | No suitable contrasts |
| Rilling, J. K. et al. | 2008 | NeuroImage | Excluded | No suitable contrasts |
| Beer, J. S. et al. | 2008 | NeuroImage | Excluded | No suitable contrasts |
| Knutson, K. M. et al. | 2007 | Human Brain Mapping | Excluded | No suitable contrasts |
| Knutson, K. M. et al. | 2006 | Social neuroscience | Excluded | No suitable contrasts |
| Apps, M. A. J. et al. | 2018 | Brain and Behavior | Excluded | Small volume correction |
| Mattan, B. D. et al. | 2018 | eNeuro | Excluded | Duplicated dataset (Mattan et al., 2018; doi:10.1093/scan/nsx128) |
| Losin, E. A. R. et al. | 2014 | Human Brain Mapping | Excluded | Duplicated dataset (Losin et al., 2012; doi:10.1016/j.neuroimage.2011.10.074) |

# Table S1.2

*Articles included based on systematic literature search via citation searching*

| **Author(s)** | **Year** | **Journal** | **Included/ Excluded** | **Primary Source** |
| --- | --- | --- | --- | --- |
| Chiao, J. Y. et al. | 2008 | Journal of Cognitive Neuroscience | Included | Meta-analysis by Saarinen et al. (2021) |
| Contreras, J. M. et al. | 2013 | PLoS One | Included | Meta-analysis by Saarinen et al. (2021) |
| Cunningham, W. A. et al. | 2004 | Psychological Science | Included | Meta-analysis by Saarinen et al. (2021) |
| Lieberman, M. D. et al. | 2005 | Nature Neuroscience | Included | Meta-analysis by Saarinen et al. (2021) |
| Liu, Y. et al. | 2015 | Human Brain Mapping | Included | Meta-analysis by Saarinen et al. (2021) |
| Molapour, T. et al. | 2015 | NeuroImage | Included | Meta-analysis by Saarinen et al. (2021) |
| Van Bavel, J. J. et al. | 2008 | Psychological Science | Included | Meta-analysis by Saarinen et al. (2021) |
| Xu, X. et al. | 2009 | Journal of Neuroscience | Included | Meta-analysis by Saarinen et al. (2021) |
| Zuo, S. et al. | 2013 | Culture and Brain | Included | Meta-analysis by Saarinen et al. (2021) |
| Falk, E. B. et al. | 2012 | Philosophical Transactions of the Royal Society B: Biological Sciences | Included | Meta-analysis by Saarinen et al. (2021) |
| Kaplan, J. T. et al. | 2007 | Neuropsychologia | Included | Meta-analysis by Saarinen et al. (2021) |
| Mitchell, G. P. et al. | 2006 | Neuron | Included | Meta-analysis by Saarinen et al. (2021) |
| Newman-Norlund, R. D. et al. | 2008 | Social Cognitive and Affective Neuroscience | Included | Meta-analysis by Saarinen et al. (2021) |
| Watson, R. et al. | 2017 | Scientific Reports | Included | Meta-analysis by Saarinen et al. (2023) |
| Earls et al. et al. | 2013 | NeuroReport | Included | Meta-analysis by Merritt et al. (2021) |
| Li et al. et al. | 2016 | NeuroImage | Included | Meta-analysis by Merritt et al. (2021) |

# Table S1.3

*Descriptive Information of Task and Comparison Types*

| **Name** | **Description** | **In-group** | | | **Out-group** | | |
| --- | --- | --- | --- | --- | --- | --- | --- |
|  |  | **No. of Contrast** | | **No. of Foci** | **No. of Contrast** | | **No. of Foci** |
| **Task Types** | | | | | | | |
| Empathy Processing | Engaging theory of mind processes, including viewing pain-related stimuli, and imitation and mentalization of in-group/ out-group members | 50 | 260 | | 24 | 167 | |
| Face Processing | Viewing visual depictions of in-group/out-group members' faces | 19 | 93 | | 15 | 80 | |
| Decision-Making | Processing scenarios assessing preferences and trade-offs, including the allocation of punishment points and rewards | 22 | 83 | | 15 | 46 | |
| Implicit Processing | Passive viewing or listening to stimuli | 17 | 82 | | 7 | 78 | |
| **Comparison Types** | | | | | | | |
| Ethnicity | Race and ethnic group (e.g., African-American vs. Caucasian-American) | 59 | 279 | | 28 | 171 | |
| Minimal | Random experimental assignment and induction (e.g., Team Red vs. Blue) | 15 | 64 | | 1 | 6 | |
| Nationality | Regional nationality (e.g., Chinese vs. Korean) | 12 | 65 | | 19 | 116 | |
| Affiliation | Established interdependent group (e.g., Own Platoon vs. Other Platoon) or political affiliations (e.g., Conservatives vs. Liberal) | 18 | 95 | | 14 | 84 | |
| Gender | Participants' biological sex (e.g., Male/ Female) | 1 | 5 | | 0 | 0 | |
| Age | Participants' chronological age (e.g., Young vs. Old) | 1 | 11 | | 0 | 0 | |
| Others | Groups involving more than one of the above types | 1 | 3 | | 0 | 0 | |

# Table S1.4

*Descriptive information of included studies, differentiated by type of group membership*

| **Author(s)** | **Year** | **Sample Size (*n*)** | **N/ Females** | **Mean age/ SD (years)** | **Type of group membership** | **Participants' in-group** | **Short description of fMRI task** |
| --- | --- | --- | --- | --- | --- | --- | --- |
| **Ethnicity** | | | | | | | |
| Mei, S. et al. | 2025 | 80 | 40/22 (Control) | 21.08/2.50 (Control) | Asian vs. White | Asian | Participants viewed video clips of white or Asian model receiving a painful stimulation (i.e., syringe penetration) to the left or right cheek that induced a pain expression or receiving a nonpainful stimulation (i.e., a cotton bud touch) and showing a neutral expression |
|  |  |  | 40/22 (Test) | 20.63/2.05 (Test) |  |  |  |
| Azevedo, R. T. et al. | 2013 | 27 | 14/7 (White Caucasian)  13/9 (Black Africans) | 23.57/4.01 (White Caucasian)  24.26/4.35 (Black Africans | White vs. Black | White-Caucasian/ Black-African | Participants observed right male hands of racial in-groups/ out-groups being either deeply penetrated by a hypodermic needle (pain condition) or touched by a Q‐tip (touch condition) |
| Berlingeri, M. et al. | 2016 | 25 | 25/13 | 25.33/4.81 | Caucasian vs. African | Caucasian | Participants viewed painful (touched by painful object) and non-painful (touched by non-painful object) experiences of in-group/ out-group members |
| Brown, T. I. et al. | 2017 | 19 | 19/0 | 23.26/4.69 | African American/ European American | African American/ European American | Participants intentionally encoded faces of in-group/ out-group members by generating imaginative stories involving in-group/ out-group members pictured in the stimuli in a manner that would facilitate their later retrieval of the faces the following day |
| Cao, Y. et al. | 2015 | 30 | 30/18 | 23.17/1.80 | Chinese vs. Caucasian | Chinese | Participants viewed video clips of Chinese or Caucasian actors who were either in-group or out-group members receiving either painful touch with a syringe needle or non-painful touch with a cotton-tip |
| Carollo, A. et al. | 2023 | 43 | 43/22 | 23.36/1.41 | Chinese vs. Indian/ Caucasian/ Arabic | Chinese | Participants passively viewed faces of typical in-group faces (Chinese), typical out-group faces (Indian), or non-typical out-group faces (Caucasian/ Arabic) |
| Cassidy, B. S. et al. | 2021 | 75 | 75/47 | 46.62/4.45 | White vs. Black | White | Participants viewed images of in-group and out-group faces |
| Chen, P. A. et al. | 2015 | 22 | 22/22 | not provided | Chinese vs. Caucasian | Chinese | Participants passively viewed facial expressions of in-group and out-group faces in varied emotions (fearful, happy, or surprised) |
| Cheon, B. K. et al. | 2011 | 27 | 13/5 (Koreans)  14/7 (Caucasians) | 23.08/4.35 (Koreans)  25.14/4.82 (Caucasians) | Korean vs Caucasians | Korean, Caucasian | Participants observed clips of in-group/ out-group members in emotional pain |
| Chiao, J. Y. et al. | 2008 | 20 | 10/10 (Japanese)  10/5 (Caucasians) | not provided | Japanese vs. Caucasian | Japanese/ Caucasian | Participants observed in-group/ out-group faces with different emotions (fear, angry, happy, or neutral) |
| Contreras-Huerta, L. S. et al. | 2013 | 20 | 20/12 | 22.5 | Caucasian vs. Chinese | Caucasian | Participants viewed video clips of in-group/ out-group members experiencing painful and non-painful touch |
| Contreras, J. M. et al. | 2013 | 17 | 17/9 | 22.18 | White vs. Black | White | Participants categorized in-group/ out-group faces by race (Black, White) |
| Cunningham, W. A. et al. | 2004 | 13 | 13/4 | 27 | White vs. Black | White | Participants pressed one of two buttons with their right hand to indicate whether faces of in-group/ out-group members appeared to the left or right of a fixation cross |
| Domínguez, D, J. F. et al. | 2018 | 48 | 48/35 | 25.3/8.92 | Muslim vs. Non-Muslim (Caucasian) | Non-Muslim (Caucasian) | Participants decided whether or not to shoot an in-group/ out-group member depending on what object (e.g., gun) they were holding |
| Earls, H. A. et al. | 2013 | 20 | 20/0 | not provided | Caucasian vs. African-American | Caucasian | Participants observed and imitated actions presented by in-group/ out-group members |
| Farmer, H. et al. | 2020 | 25 | 25/17 | 25.16/4.56 | White vs. Black | White | Participants viewed faces of in-group/ out-group members and categorised them on the basis of age (over or under 25 years old) or their favourite vegetables (as taught in the learning task) |
| Feng, L. et al. | 2011 | 30 | 30/11 | 23 | Chinese vs. Caucasian | Chinese | Participants viewed faces of in-group/ out-group members and categorised them based on race or the degree of similarity with a previously presented face |
| Firat, R. B. et al. | 2017 | 13 | 13/6 | 47.2/7.6 | White vs. Black | Whites | Participants viewed pictures of in-group/ out-group members in different socio-economic positions [lower class (e.g., homeless people); middle class (e.g., people barbecuing); upper class (e.g., people wearing upscale clothing and jewelry)] with one of eight primary emotions expressed (e.g., happy, pride, sad) |
| Fourie, M. M. et al. | 2017 | 38 | 19/11 (Black)  19/10 (White) | 40.11/4.12 (Black)  41.47/5.80 (White) | White vs. Black | Black/ White | Participants viewed faces of in-group/ out-group members expressing either physical pain, social pain (due to loss of loved one, physical, or sexual violence) or a neutral expression (no pain) |
| Freeman, J. B. et al. | 2010 | 16 | 16/8 | not provided | White vs. Black | White | Participants read statements (superficial/ individuated) about the personality of in-group/ out-group members and judged by a button press whether those statements were true or false |
| Gilbert, S. J. et al. | 2012 | 16 | 16/16 | not provided | White vs. Black | White | Participants viewed a pair of in-group/ out-group faces and judged them according to which in-group/ out-group member is more likely to enjoy athletic activities (trait judgement) and which in-group/ out-group member is more likely to befriend (friendship judgement) |
| Handley, G. et al. | 2023 | 58 | 58/28 | 24.72/6.87 | White vs. Black | White | Participants performed a Reading the Mind in the Eyes (RME) test - viewed a cropped images depicting a pair of in-group/ out-group member's eyes and selected which secondary emotion (out of four) possible options those eyes convey |
| Katsumi, Y. et al. | 2018 | 20 | 20/10 | not provided | Caucasian vs. East Asian/ South Asian/ African-American | Caucasian | Participants observed in-group/ out-group members displaying dynamic non-verbal behaviors that either encourage (approach: open postures, smiley faces) or discourage (avoidance: closed postures, frowny faces) further social interaction |
| Krosch, A. R. et al. | 2019 | 30 | 30/16 | 19.63/1.40 | White vs. Black | White | Participants viewed in-group/ out-group faces and allocated monetary resources to them in scarce (assigned $10 to allocate, but was informed they had up to $100 to allocate) and neutral (up to $10 to allocate) trials |
| Lee, K. U. et al. | 2008 | 13 | 13/0 | 24.8/3.6 | Korean vs. Caucasian | Korean | Participants performed a sex discrimination task to elucidate incidental processing of different race emotional faces - press a button when male in-group (White)/ out-group (Black) members were shown on the screen |
| Li, T. et al. | 2016 | 44 | 44/24 | 24.4/4.32 | White vs. Black | White | Participants performed an impression formation task in which they formed impressions of out-group (Black) and in-group (White) members' faces paired with either positive or negative words |
| Li, X. et al. (Experiment 2; Mortality Salience Group) | 2015 | 20 | 20/9 | 23.15/2.35 | East Asian vs. Caucasian | East Asian | Participants viewed facial expression (pain vs. neutral) of in-group/ out-group members (after having primed with mortality salience/ negative affect) |
| Li, X. et al. (Experiment 2; Negative Affect Group) | 2015 | 20 | 20/12 | 21.95/2.09 | East Asian vs. Caucasian | East Asian |  |
| Lieberman, M. D. et al. | 2005 | 20 | 11/11 (Caucasian-American  9/9 (African-American) | 23.7 (Caucasian-American)  24.9 (African-American) | African-American vs Caucasian-American | African-American/ Caucasian-American | Participants performed a perceptual encoding task (view and select a face that matched in-group/ out-group target's in terms of race) and verbal encoding task (select the race label that described the race of the in-group/ out-group target's face) |
| Lin, L. C. et al. | 2018 | 45 | 45/24 | 19.42/0.63 | American vs. Chinese | American/ Chinese | Participants performed a social influence task across three phases in which they (1) rated a set of image, (2) viewed how in-group and out-group members rated a subset of the images they had rated, and then (3) rated the images again themselves |
| Liu, Y. et al. | 2015 | 26 | 26/13 | 21.2/1.8 | Chinese vs. African | Chinese | Participants passively viewed faces of in-group/ out-group members in disgust presented on the screen without further cognitive processes |
| Losin, E. A. R. et al. | 2012 | 20 | 20/10 | 22.92/2.09 | European American vs. African American/ Han Chinese | European American | Participants passively observed and imitated in-group/ out-group members perform different hand signs |
| Luo, S. et al. (A/A Genotype Group) | 2015 | 30 | 30/14 (A/A) | 20.20/1.45 (A/A) | Asian vs. Caucasian | Asian | Participants viewed in-group/ out-group members receive painful (needle penetration) or non-painful (Q-tip touch) stimuli applied to the left or right cheeks while showing neutral expressions |
| Luo, S. et al. (G/G Genotype Group) | 2015 | 30 | 30/14 (G/G) | 20.33/1.65 (G/G) | Asian vs. Caucasian | Asian |  |
| Mathur, V. A. et al. (African-American Group) | 2012 | 10 | 10/6 | 23.1 | African-American vs. Caucasian-American | African-American | Participants viewed in-group/ out-group members in emotionally painful (e.g., in the midst of a natural disaster) or neutral (e.g., attending an outdoor picnic) situation and indicated the extent to which they felt empathy for in-group/ out-group members |
| Mathur, V. A. et al. (Caucasian-American Group) | 2012 | 10 | 10/9 | 23.3 | African-American vs. Caucasian-American | Caucasian-American |  |
| Mathur, V. A. et al. (African-American Group) | 2010 | 14 | 14/10 | not provided | African-American vs. Caucasian-American | African-American | Participants observed in-group/ out-group members in a painful (e.g., in the midst of a natural disaster) or neutral (e.g., attending an outdoor picnic) situation |
| Mathur, V. A. et al. (Caucasian-American Group) | 2010 | 14 | 14/13 | not provided | African-American vs. Caucasian-American | Caucasian-American |  |
| Mathur, V. A. et al. (Full Sample) | 2010 | 28 | 14/10 (African-American)  14/13 (Caucasian-American) | not provided | African-American vs. Caucasian-American | African-American/ Caucasian-American |  |
| Mattan, B. D. et al. | 2018 | 60 | 60/0 | 23.8/4.59 | White vs. Black | White | Participants performed an impression-formation task - viewed faces of in-group/ out-group members with varying SES |
| McCutcheon, R. et al. (Black Group) | 2018 | 17 | 17/10 (Blacks) | 24.5 (Blacks) | White vs. Black | Black | Participants viewed faces of in-group/ out-group members |
| McCutcheon, R. et al. (White Group) | 2018 | 19 | 19/10 (Whites) | 24.0 (Whites) | White vs. Black | White |  |
| Molapour, T. et al. | 2015 | 20 | 20/10 | 22.39/3.82 | White vs. Black | White | Participants were classically conditioned with electric shocks across three phases: habituation (view images of in-group/ out-group), acquisition (receive shocks when viewing in-group/ out-group), and extinction (view images of in-group/ out-group without shocks) |
| Rauchbauer, B. et al. | 2015 | 41 | 41/18 | 22.63/2.86 | European-Caucasian vs. African-American | European-Caucasian | Participants performed a social-affective mimicry task: imitated in-group/ out-group members' hand movement in congruent (movement of the same finger as the one required to lift by the participant) and incongruent (movement of the other finger as the one required to lift by the participant) trials |
| Richeson, J. A. et al. | 2003 | 15 | 15/8 | 20.5 | White vs. Black | White | Participants viewed images of in-group/ out-group members faces |
| Ronquillo, J. et al. | 2007 | 11 | 11/0 | not provided | White vs. Black | White | Participants categorised dark/light-toned faces of in-group/ out-group members to older or younger than 24 |
| Rubien-Thomas, E. et al. | 2021 | 106 | 106/57 | 26.08 | White vs. Black | White/ Black | Participants performed go/no-go task - press a button as quickly as possible for in-groups ("Only press to male faces") and withhold a response for out-groups |
| Rule, N. O. et al. | 2010 | 28 | 14/14 (Americans)  14/14 (Japanese) | not provided | American vs. Japanese | American/ Japanese | Participants viewed faces of in-group/ out-group members and indicated via button-press whether they would or would not vote for each person |
| Sheng, F. et al. | 2014 | 21 | 21/11 | 22.0/1.8 | Asian vs. Caucasian | Asian | Participants simultaneously performed a race judgment task (identify race of in-group/ out-group members while ignoring facial expressions) and pain judgment task (identify facial expression of in-group/ out-group members (pain vs. neutral)) while ignoring its race |
| Telzer, E. H. et al. | 2015 | 26 | 13/7 (American)  13/6 (Chinese) | 19.02 (American)  19.38 (Chinese) | American vs. Chinese | American/ Chinese | Participants performed a prosocial task (modified dictator game) in which they were presented with financial offers and were asked to keep the money for themselves or donate a sum of money to in-group/ out-group members |
| Van Bavel, J. J. et al. | 2008 | 17 | 17/10 | not provided | White vs. Black | White | Participants viewed and categorized faces of in-group/ out-group members according to team membership or race |
| Wang, C. et al. | 2015 | 30 | 30/14 | 22.6/2.4 | Asian vs. Caucasian | Asian | Participants viewed video clips of in-group/ out-group models receiving painful/ non-painful stimuli |
| Xu, X. et al. (Caucasian Group) | 2009 | 16 | 16/8 | 23/3.7 | Caucasian vs. Chinese | Caucasian | Participants viewed in-group/ out-group member receiving painful (needle penetration) or non-painful (Q-tip touch) stimulation and judged whether or not the model was feeling pain |
| Xu, X. et al. (Chinese Group) | 2009 | 17 | 17/9 | 23/2 | Caucasian vs. Chinese | Chinese |  |
| Yan, Z. et al. | 2019 | 44 | 20/9 (Chinese)  24/12 (German) | 26.02/2.82 (Chinese)  25.38/5.44 (German) | Chinese vs. German | Chinese/ German | Participants viewed and categorized pictures of in-group/ out-group faces according to the corresponding group previously taught prior to fMRI scanning |
| Zuo, S. et al. | 2013 | 20 | 20/12 | 23.3/3.39 | Chinese vs. Caucasian | Chinese | Participants viewed in-group/ out-group member receiving painful (needle penetration) or non-painful (Q-tip touch) stimulation and judged whether or not the model was feeling pain |
| **Affiliation** | | | | | | | |
| Fang, Z. et al. | 2024 | 26 | 25/9 | 21.62 | League of Legends vs. Honor of Kings Players | League of Legends Players | Participants carried out a dot estimation task with another partner (in-group/ out-group) and received feedback about their performance in the task in relation to in-group/ out-group member |
| Zacharopoulos, G. et al. | 2023 | 20 | 20/10 | 22.6 | Cardiff University vs. Swansea University | Cardiff University | Participants compared the beauty of two women either from their ingroup or from an outgroup |
| Baumgartner, T. et al. | 2012 | 16 | 16/0 | 24.5/2.2 | Own Platoon vs. Other Platoon | Own Platoon | Participants adopted the role of a third-party in a Prisoners' Dilemma Game confronted with decisions of in-group/ out-group members and assigned punishment points to them |
| Cui, F. et al. | 2023 | 28 | 28/14 | 21.3/2.1 | Shenzhen University vs. Different University (not specified) | Shenzhen University | Participants first observe the confederate (in-group or out-group member) and how much Monetary Units they were assigned that trial, which were either scarce (not enough for two players to escape physical threat) or abundant (enough for two players to escape physical threat), and then decides how much Monetary Units to share to reduce a physical threat (unpleasant noise). |
| Falk, E. B. et al. | 2012 | 23 | 23/10 | 24/5.65 | Obama supporters vs. McCain supports | Obama/ McCain supporters | Participants responded to a range of issues relevant to the 2008 election from the perspectives of each of in-group/ out-group members |
| Fox, G. R. et al. | 2013 | 16 | 16/0 | 22/3.5 | Jewish vs. Neo-Nazi | Jewish | Participants viewed clips of in-group/ out-group members receiving a painful injection to the palm of the hand |
| Hein, G. et al. | 2010 | 16 | 16/0 | 29.8 | Same football team vs. Rival soccer team | Same football team | Participants observed in-group/ out-group members receive painful stimulation of varied intensity (low, medium, high) |
| Kang, P. et al. | 2021 | 29 | 29/19 | 22.51/0.54 | Left-wing vs. Right-wing political attitudes | Left-wing | Participants performed an observational learning task - learn about the reward probablity of two fractal images from in-group/ out-group demonstrators by viewing their actions only (action-based learning) or their actions and the outcomes associated with them (outcome-based learning) |
| Kaplan, J. T. et al. | 2007 | 20 | 20/10 | 35.7/6.34 (Democrats)  35.6/9.29 (Republicans) | Democrats vs. Republicans | Democrat/ Republican | Participants viewed images of their supporting (in-group)/ opposing (out-group) presidential candidates (George Bush/ John Kerry) |
| Kim, K. et al. | 2015 | 24 | 24/14 | 21.67/3.23 | Liberal vs. Conservative | Liberal | Participants performed an Ownership Imagination task in which they imagined owning objects that were associated with in-group or out-group members |
| Krendl, A. C. et al. | 2009 | 65 | 42/19 (Older Adults)  23/12 (Young Adults) | 73.14 (Older Adults)  19.53 (Young Adults) | Stigmatised Individuals (e.g., individuals with amputations) vs. Typical | Typical | Participants viewed images of stigmatised (out-group, e.g., persons with amputations/ deformities) and non-stigmatised (in-group, control, e.g., persons with no visible stigma) members |
| Lau, T. et al. | 2017 | 22 | 22/12 | 25.25/2.77 | Political Party Affiliation: Democrats vs. Republicans | Democrats | Participants performed repetition suppression and enhancement task - read paired statements (e.g., "Sam is a Democrat" and "Y is an Eagle") about two targets and asked to indicate if they belong to one of three conditions: ‘identical in-group’ (Democrat-Democrat or Eagles-Eagles), ‘different in-group’ (Eagles-Democrat or Democrat-Eagles), and ‘out-group/in-group trials’ (Republican-Democrat or Rattler-Eagles) |
| Mitchell, G. P. et al. | 2006 | 15 | 15/6 | 24.4 | Liberal vs. Conservative | Liberal/ Conservative | Participants judged how likely in-group/ out-group members were to agree with several opinion questions relating to personal (e.g., to look forward to going home for Thanksgiving?) and societal issues (e.g., to believe that cultural diversity should be an important national issue?) |
| Molenberghs, P. et al. | 2016 | 48 | 48/24 | 22.2/5.3 | UQ students vs. QUT students | UQ | Participants passively watched a video of in-group/ out-group member (perpetrator) intentionally harming another in-group/ out-group member (victim) |
| Molenberghs, P. et al. | 2017 | 40 | 40/20 | 20.6 | Political Affiliation: Liberals vs. Labor | Liberals/ Labors | Participants were presented with inspirational collective-oriented (e.g., "We will work together and shape a future of indiscriminate prosperity") and non-inspirational personal-oriented statements by in-group/ out-group political members |
| Moradi, Z. et al. | 2017 | 20 | 20/1 | 31/7.50 | Favourite football team vs. Rival team | Favourite football team | Participants performed associative learning task - learn the association between arbitrary geometric shapes and the badges of in-group/ out-group teams in match (both shape/ badge belong to the same in-group/ out-group) and unmatched (shape/ badge deviates from the same group) trials |
| Newman-Norlund, R. D. et al. | 2008 | 22 | 22/1 | 24 | Netherlands vs. Germany | Netherlands/ Germany | Participants viewed clips of penalty kicks for in-group/ out-group football teams and asked to judge if it was a goal or miss |
| Nugiel, T. et al. | 2020 | 50 | 50/36 | 22.18/4.70 | Political Affiliation: Democratic vs. Republican | Democratic | Participants judged (Yes/ No) whether in-group/ out-group members possessed various positive (e.g., Does NOBLE describe Barack Obama?) and/or negative (e.g., Does PHONY describe Barack Obama?) personality traits |
| Richins, M. T. et al. | 2019 | 69 | 69/42 | 20.57/3.04 | University of Exeter vs. Cardiff University vs. University of Sussex | Exeter students | Participants viewed photos of individuals that were denoted as either in-group members or out-group members experiencing painful or innocuous events |
| Wu, C. T. et al. | 2018 | 54 | 27/16 (KMT)  27/18 (DDP) | 23.30/0.67 (KMT)  24.22/4.60 (DDP) | KMT Candidate voters vs. DPP Candidate voters | KMT/ DDP | Participants played a binary trust game - decide whether or not to invest monetary units (MU) to in-group/ out-group members and participants can decide to reciprocate (to split the money equally so that in-group/out-group member and the participant each receives 20 MU) or defect (keep the entire amount of 40 MU and in-group/out-group member receives 0 MU). |
| **Minimal** | | | | | | | |
| Cao, Y. et al. | 2015 | 30 | 30/18 | 23.17/1.80 | Experimentally assigned: Similar Beliefs vs. Different Beliefs | Similar Beliefs | Participants viewed video clips of Chinese or Caucasian actors who were either in-group or out-group members receiving either painful touch with a syringe needle or non-painful touch with a cotton-tip |
| Contreras-Huerta, L. S. et al. | 2013 | 20 | 20/12 | 22.5 | Own-group vs. Other-group | Own-group | Participants viewed video clips of in-group/ out-group members experiencing painful and non-painful touch |
| Feng, C. et al. (Mortality Salience Group) | 2017 | 20 | 20/20 | 21.2/2.7 | Experimentally assigned: Yellow vs. Red Group | Yellow/ Red | Participants decided how to punish in-group/ out-group members (after viewing them offering fair/ unfair monetary proposals) by reducing their payoffs |
| Feng, C. et al. (Neutral Group) | 2017 | 20 | 20/20 | 22.1/2.6 | Experimentally assigned: Yellow vs. Red Group | Yellow/ Red |  |
| Lau, T. et al. | 2017 | 22 | 22/12 | 25.25/2.77 | Experimentally Assigned Group: Eagles vs. Rattlers | Eagles | Participants performed repetition suppression and enhancement task - read paired statements (e.g., "Sam is a Democrat" and "Y is an Eagle") about two targets and asked to indicate if they belong to one of three conditions: ‘identical in-group’ (Democrat-Democrat or Eagles-Eagles), ‘different in-group’ (Eagles-Democrat or Democrat-Eagles), and ‘out-group/in-group trials’ (Republican-Democrat or Rattler-Eagles) |
| Li, Z. et al. | 2020 | 31 | 31/19 | 21.3/1.1 | Experimentally assigned Group Yellow vs. Group Blue | Group Yellow/ Group Blue | Participants witnessed their own causal contribution to in-group/ out-group member receiving painful electric stimulation |
| Littlefield, M. M. et al. | 2015 | 23 | 23/18 | not provided | Experimentally assigned Blue vs. Purple group | Blue/ Purple Group | Participants performed a truth telling task in which they viewed faces of in-group/ out-group members and responded to questions that concern telling a social truth (e.g., is Person X a poor singer?) or a simple truth (e.g., does Person X have brown hair?) |
| Marsh, L. E. et al. | 2016 | 24 | 24/17 | 23.71 | Experimentally assigned Team Red vs. Blue | Team Red/ Blue | Participants viewed and imitated in-group/ out-group members' hand gesture that was either the same finger (imitatively congruent) or a different finger (imitatively incongruent) on the same side of space (spatially congruent) or a different side of space (spatially incongruent) to that shown |
| Molenberghs, P. et al. | 2013 | 24 | 24/0 | 23.8 | Experimentally assigned Red vs. Blue team | Red/ Blue team | Participants passively viewed in-group team and out-group team members perform hand actions |
| Molenberghs, P., Bosworth, R. et al. | 2014 | 48 | 48/24 | 22.5/4.9 | Experimentally assigned Red vs. Green team | Red/ Green team | Participants performed a moral evaluation task where they allocated rewards (i.e., money when in-group/ out-group member respond correctly to a question posed) and punishments (i.e., shocks when in-group/out-group did not respond correctly to a question posed) to in-group/ out-group members |
| Molenberghs, P., Morrison, S. et al. | 2014 | 20 | 20/14 | 23/4 | Experimentally assigned Red vs. Blue team | Red/ Blue team | Participants categorized Red Team and Blue Team words as My Team (in-group) and Other Team (out-group) by pressing a left or right button to indicate the side of the matching stimulus |
| Ruckmann, J. et al. | 2015 | 30 | 30/15 | 24.50/3.36 | Experimentally assigned problem solver type (conclusive vs. sequential) | Experimentally assigned problem solver type (conclusive vs. sequential) | Participants viewed photos showing in-group/ out-group members' right hands and feet in painful and neutral everyday situations |
| Steines, M. et al. | 2020 | 22 | 22/14 | 26/4.6 | Minimal: Problem solver type (conclusive vs. sequential) | Minimal: experimentally assigned | Participants viewed pictures of in-group/ out-group members facial emotions (anger/ neutral) |
| Van Bavel, J. J. et al. | 2008 | 17 | 17/10 | not provided | Experimentally assigned Team Leopards/ Tigers | Team Leopards/ Team Tigers | Participants viewed and categorized faces of in-group/ out-group members according to team membership or race |
| Volz, K. G. et al. | 2009 | 20 | 20/10 | 25.3/2.5 | Experimentally assigned Yellow or Blue | Yellow/ Blue Group | Participants assigned money to different in-group/ out-group members |
| Yan, Z. et al. | 2019 | 44 | 20/9 (Chinese)  24/12 (German) | 26.02/2.82 (Chinese)  25.38/5.44 (German) | Experimentally Assigned: Team Green vs. Magenta | Experimentally assigned | Participants viewed and categorized pictures of in-group/ out-group faces according to the corresponding group previously taught prior to fMRI scanning |
| **Nationality** | | | | | | | |
| Mauchaund, M. et al. | 2023 | 24 | 24/13 | 26.04/3.99 | French vs. Quebecois | French | Participants listened to short utterances describing a painful event, which were either produced in a neutral-sounding or complaining voice by both in-group (French) and out-group (French Canadian) speakers |
| Bestelmeyer, P. E. et al. | 2015 | 40 | 20/11 (Scottish)  20/8 (Southern English) | 23.45/3.62 (Scottish)  18.80/1.44 (Southern English) | English vs. Scottish | English/ Scottish | Participants listened passively to accents of in-group/ out-group members |
| Bruneau, E. G. et al. | 2012 | 24 | 10/10 (Arab)  14/14 (Israeli) | 25.1 (Arab)  29.6 (Israeli) | Israeli vs Arab | Arab/ Israeli | Participants read short verbal narratives of in-group/ out-group members in physically/ emotionally or non-painful scenarios |
| Bruneau, E. G. et al. (Arab Group) | 2010 | 16 | 16/16 (Non-Israeli Arab) | 21.5 (Non-Israeli Arab) | Israeli vs Arab | Arab | Participants read statements (e.g., information about Israeli/ Arabic society/ history/ economy) about in-group or out-group members and rated the reasonableness of each statement on a button press |
| Bruneau, E. G. et al. (Israeli Group) | 2010 | 16 | 16/16 (Jewish Israeli) | 29.0 (Jewish Israeli) | Israeli vs Arab | Israeli |  |
| Feng, C. et al. (Mortality Salience Group) | 2017 | 20 | 20/20 | 21.2/2.7 | Chinese vs. Korean | Chinese | Participants decided how to punish in-group/ out-group members (after viewing them offering fair/ unfair monetary proposals) by reducing their payoffs |
| Feng, C. et al. (Neutral Group) | 2017 | 20 | 20/20 | 22.1/2.6 | Chinese vs. Korean | Chinese |  |
| Hein, G. et al. (Control Group) | 2016 | 18 | 18/0 | not provided | Swiss vs. Balkan | Swiss | Participants observed the in-group or the out-group confederate receive painful stimulation |
| Hein, G. et al. (Experimental Group) | 2016 | 20 | 20/0 | not provided | Swiss vs. Balkan | Swiss |  |
| Izuma, K. et al. | 2019 | 70 | 70/27 | 18.9/1.11 | South Korea vs. Japan | Japanese | Participants viewed images related to in-group/ out-group (e.g., people/ national flags) [Note: The fMRI task comprises both face processing and the processing of stimuli unrelated to face, such as flags] |
| Jiang, X. et al. | 2018 | 25 | 25/25 | not provided | Canadian vs. American/French speakers | Canadian | Participants listened to audio recordings of English speakers and judged the extent to which they believe the statements were produced by in-group (native) or out-group (foreign/ regional) speakers |
| Raghunath, B. L. et al. | 2022 | 27 | 27/16 | 24.91/4.93 | Chinese vs. Indian | Chinese | Participants viewed photos of in-group/ out-group infant faces |
| Scheepers, D. et al. | 2013 | 41 | 41/0 | 21 | Leiden University vs. VU University Amsterdam | Leiden University | Participants observed pictures of an in-group or out-group member |
| Steines, M. et al. | 2020 | 22 | 22/14 | 26/4.6 | German vs. Turkish | German | Participants viewed pictures of in-group/ out-group members facial emotions (anger/ neutral) |
| van Gils, S. et al. | 2020 | 17 | 17/17 | not provided | Dutch university vs. German university | Dutch university | Participants were presented with a trolley dilemma situation, and asked to either sacrifice (a) an out-group member, to save a group of in-group members, or (b) an in-group member, to save a group of out-group members |
| Watson, R. et al. | 2017 | 21 | 21/13 | 22/3.22 | European vs. Black African | European | Participants viewed affective (angry and happy) body postures of both same race (white; in-group) and other race (black; out-group) individuals, while asked to perform an emotion categorisation task (indicate whether the in-group/ out-group member's posture was happy or angry) or a shape categorisation task (indicate whether a circle or square was superimposed on in-group/ out-group member's body) |
| **Gender** | | | | | | | |
| Losin, E. A. et al. | 2012 | 19 | 19/10 | 22.92/2.09 | Men vs. Women | Men/ Women | Participants imitated gestures performed by in-group/ out-group members |
| **Age** | | | | | | | |
| Ebner, N. C. et al. | 2013 | 62 | 30/16 (Young Adults)  32/18 (Older Adults) | 25.1/3.4 (Young Adults)  68.2/2.5 (Older Adults) | Own-age vs. Other-age | Own-age, Other-age | Participants performed Facial Expression Identification Task - indicate whether in-group/ out-group faces displayed a happy, neutral, or angry expression |
| **Others (i.e., groups involving more than one of the above types)** | | | | | | | |
| Morrison, S. et al. | 2012 | 20 | 20/14 | 22.9 | Participants' choice of 7 in-groups (any category, e.g., Gender, Nationality, Religious Affiliations, etc.) | Participants' choice of 7 in-groups | Participants categorized words on the basis of whether they belong to in-group (my team) or out-group (other team) |

# Table S1.5

*Descriptive information of included studies, differentiated by type of task*

| **Author(s)** | **Year** | **Sample Size (*n*)** | **N/ Females** | **Mean age/ SD (years)** | **Type of group membership** | **Participants' in-group** | **Short description of fMRI task** |
| --- | --- | --- | --- | --- | --- | --- | --- |
| **Empathy Processing** | | | | | | | |
| Mei, S. et al. | 2025 | 80 | 40/22 (Control) | 21.08/2.50 (Control) | Asian vs. White | Asian | Participants viewed video clips of white or Asian model receiving a painful stimulation (i.e., syringe penetration) to the left or right cheek that induced a pain expression or receiving a nonpainful stimulation (i.e., a cotton bud touch) and showing a neutral expression |
|  |  |  | 40/22 (Test) | 20.63/2.05 (Test) |  |  |  |
| Azevedo, R. T. et al. | 2013 | 27 | 14/7 (White Caucasian)  13/9 (Black-Africans) | 23.57/4.01 (White)  24.26/4.35 (Black) | White vs. Black | White-Caucasian/ Black-African | Participants observed right male hands of racial in-groups/ out-groups being either deeply penetrated by a hypodermic needle (pain condition) or touched by a Q‐tip (touch condition) |
| Baumgartner, T. et al. | 2012 | 16 | 16/0 | 24.5/2.2 | Own Platoon vs. Other Platoon | Own Platoon | Participants adopted the role of a third-party (in a Prisoners' Dilemma Game) confronted with decisions of in-group/ out-group members and assigned punishment points to them |
| Berlingeri, M. et al. | 2016 | 25 | 25/13 | 25.33/4.81 | Caucasian vs. African | Caucasian | Participants viewed painful (touched by painful object) and non-painful (touched by non-painful object) experiences of in-group/ out-group members |
| Bruneau, E. G. et al. | 2012 | 24 | 10 (Arab)  14 (Israeli) | 25.1 (Arab)  29.6 (Israeli) | Israeli vs Arab | Arab/ Israeli | Participants read short verbal narratives of in-group/ out-group members in physically/ emotionally or non-painful scenarios |
| Cao, Y. et al. | 2015 | 30 | 30/18 | 23.17/1.80 | Chinese vs. Caucasian  Experimentally assigned: Similar Beliefs vs. Different Beliefs | Chinese  Similar Beliefs | Participants viewed video clips of Chinese or Caucasian actors who were either in-group or out-group members receiving either painful touch with a syringe needle or non-painful touch with a cotton-tip |
| Chen, P. A. et al. | 2015 | 22 | 22/22 | not provided | Chinese vs. Caucasian | Chinese | Participants passively viewed facial expressions of in-group and out-group faces in varied emotions (fearful, happy, or surprised) |
| Cheon, B. K. et al. | 2011 | 27 | 13/5 (Koreans)  14/7 (Caucasians) | 23.08/4.35 (Koreans)  25.14/4.82 (Caucasians) | Korean vs Caucasians | Korean, Caucasian | Participants observed clips of in-group/ out-group members in emotional pain |
| Chiao, J. Y. et al. | 2008 | 20 | 10/10 (Japanese)  10/5 (Caucasians) | not provided | Japanese vs. Caucasian | Japanese/ Caucasian | Participants observed in-group/ out-group faces with different emotions (fear, angry, happy, or neutral) |
| Contreras-Huerta, L. S. et al. | 2013 | 20 | 20/12 | 22.5 | Caucasian vs. Chinese  Own-group vs. Other-group | Caucasian  Own-group | Participants viewed video clips of in-group/ out-group members experiencing painful and non-painful touch |
| Earls, H. A. et al. | 2013 | 20 | 20/0 | not provided | Caucasian vs. African-American | Caucasian | Participants observed and imitated actions presented by in-group/ out-group members |
| Falk, E. B. et al. | 2012 | 23 | 23/10 | 24/5.65 | Obama supporters vs. McCain supports | Obama/ McCain supporters | Participants responded to a range of issues relevant to the 2008 election from the perspectives of each of in-group/ out-group members |
| Fourie, M. M. et al. | 2017 | 38 | 19/11 (Black)  19/10 (White) | 40.11/4.12 (Black)  41.47/5.80 (White) | White vs. Black | Black/ White | Participants viewed faces of in-group/ out-group members expressing either physical pain, social pain (due to loss of loved one, physical, or sexual violence) or a neutral expression (no pain) |
| Fox, G. R. et al. | 2013 | 16 | 16/0 | 22/3.5 | Jewish vs. Neo-Nazi | Jewish | Participants viewed clips of in-group/ out-group members receiving a painful injection to the palm of the hand |
| Handley, G. et al. | 2023 | 58 | 58/28 | 24.72/6.87 | White vs. Black | White | Participants performed a Reading the Mind in the Eyes (RME) test - viewed a cropped images depicting a pair of in-group/ out-group member's eyes and selected which secondary emotion (out of four) possible options those eyes convey |
| Hein, G. et al. | 2010 | 16 | 16/0 | 29.8 | Same football team vs. Rival soccer team | Same football team | Participants observed in-group/ out-group members receive painful stimulation of varied intensity (low, medium, high) |
| Hein, G. et al. (Control Group) | 2016 | 18 | 18/0 | not provided | Swiss vs. Balkan | Swiss | Participants observed the in-group or the out-group confederate receive painful stimulation |
| Hein, G. et al. (Experimental Group) | 2016 | 20 | 20/0 | not provided | Swiss vs. Balkan | Swiss | Participants observed the in-group or the out-group confederate receive painful stimulation |
| Katsumi, Y. et al. | 2018 | 20 | 20/10 | not provided | Caucasian vs. East Asian/ South Asian/ African-American | Caucasian | Participants observed in-group/ out-group members displaying dynamic non-verbal behaviors that either encourage (approach: open postures, smiley faces) or discourage (avoidance: closed postures, frowny faces) further social interaction |
| Kim, K. et al. | 2015 | 24 | 24/14 | 21.67/3.23 | Liberal vs. Conservative | Liberal | Participants performed an Ownership Imagination task in which they imagined owning objects that were associated with in-group or out-group members |
| Lee, K. U. et al. | 2008 | 13 | 13/0 | 24.8/3.6 | Korean vs. Caucasian | Korean | Participants performed a sex discrimination task to elucidate incidental processing of different race emotional faces - press a button when male in-group (White)/ out-group (Black) members were shown on the screen |
| Li, X. et al. (Experiment 2; Mortality Salience Group) | 2015 | 20 | 20/9 | 23.15/2.35 | East Asian vs. Caucasian | East Asian | Participants viewed facial expression (pain vs. neutral) of in-group/ out-group members (after having primed with mortality salience/ negative affect) |
| Li, X. et al. (Experiment 2; Negative Affect Group) | 2015 | 20 | 20/12 | 21.95/2.09 | East Asian vs. Caucasian | East Asian | Participants viewed facial expression (pain vs. neutral) of in-group/ out-group members (after having primed with mortality salience/ negative affect) |
| Li, Z. et al. | 2020 | 31 | 31/19 | 21.3/1.1 | Experimentally assigned Group Yellow vs. Group Blue | Group Yellow/ Group Blue | Participants witnessed their own causal contribution to in-group/ out-group member receiving painful electric stimulation |
| Liu, Y. et al. | 2015 | 26 | 26/13 | 21.2/1.8 | Chinese vs. African | Chinese | Participants passively viewed faces of in-group/ out-group members in disgust presented on the screen without further cognitive processes |
| Losin, E. A. et al. | 2012 | 19 | 19/10 | 22.92/2.09 | Men vs. Women | Men/ Women | Participants imitated gestures performed by in-group/ out-group members |
| Losin, E. A. R. et al. | 2012 | 20 | 20/10 | 22.92/2.09 | European American vs. African American/ Han Chinese | European American | Participants passively observed and imitated in-group/ out-group members perform different hand signs |
| Luo, S. et al. (A/A Genotype Group) | 2015 | 30 | 30/14 (A/A) | 20.20/1.45 (A/A) | Asian vs. Caucasian | Asian | Participants viewed in-group/ out-group members receive painful (needle penetration) or non-painful (Q-tip touch) stimuli applied to the left or right cheeks while showing neutral expressions |
| Luo, S. et al. (G/G Genotype Group) | 2015 | 30 | 30/14 (G/G) | 20.33/1.65 (G/G) | Asian vs. Caucasian | Asian |  |
| Marsh, L. E. et al. | 2016 | 24 | 24/17 | 23.71 | Experimentally assigned Team Red vs. Blue | Team Red/ Blue | Participants viewed and imitated in-group/ out-group members' hand gesture that was either the same finger (imitatively congruent) or a different finger (imitatively incongruent) on the same side of space (spatially congruent) or a different side of space (spatially incongruent) to that shown |
| Mathur, V. A. et al. (African-American Group) | 2012 | 10 | 10/6 | 23.1 | African-American vs. Caucasian-American | African-American | Participants viewed in-group/ out-group members in emotionally painful (e.g., in the midst of a natural disaster) or neutral (e.g., attending an outdoor picnic) situation and indicated the extent to which they felt empathy for in-group/ out-group members |
| Mathur, V. A. et al. (Caucasian-American Group) | 2012 | 10 | 10/9 | 23.3 | African-American vs. Caucasian-American | Caucasian-American |  |
| Mathur, V. A. et al. (African-American Group) | 2010 | 14 | 14/10 | not provided | African-American vs. Caucasian-American | African-American | Participants observed in-group/ out-group members in a painful (e.g., in the midst of a natural disaster) or neutral (e.g., attending an outdoor picnic) situation |
| Mathur, V. A. et al. (Caucasian-American Group) | 2010 | 14 | 14/13 | not provided | African-American vs. Caucasian-American | Caucasian-American |  |
| Mathur, V. A. et al. (Full Sample) | 2010 | 28 | 14/10 (African-American)  14/13 (Caucasian-American) | not provided | African-American vs. Caucasian-American | African-American/ Caucasian-American |  |
| Rauchbauer, B. et al. | 2015 | 41 | 41/18 | 22.63/2.86 | European-Caucasian vs. African-American | European-Caucasian | Participants performed a social-affective mimicry task: imitated in-group/ out-group members' hand movement in congruent (movement of the same finger as the one required to lift by the participant) and incongruent (movement of the other finger as the one required to lift by the participant) trials |
| Ruckmann, J. et al. | 2015 | 30 | 30/15 | 24.50/3.36 | Experimentally assigned problem solver type (conclusive vs. sequential) | Experimentally assigned problem solver type (conclusive vs. sequential) | Participants viewed photos showing in-group/ out-group members' right hands and feet in painful and neutral everyday situations |
| Sheng, F. et al. | 2014 | 21 | 21/11 | 22.0/1.8 | Asian vs. Caucasian | Asian | Participants simultaneously performed a race judgment task (identify race of in-group/ out-group members while ignoring facial expressions) and pain judgment task (identify facial expression of in-group/ out-group members (pain vs. neutral)) while ignoring its race |
| Wang, C. et al. | 2015 | 30 | 30/14 | 22.6/2.4 | Asian vs. Caucasian | Asian | Participants viewed video clips of in-group/ out-group models receiving painful/ non-painful stimuli |
| Watson, R. et al. | 2017 | 21 | 21/13 | 22/3.22 | European vs. Black African | European | Participants viewed affective (angry and happy) body postures of both same race (white; in-group) and other race (black; out-group) individuals, while asked to perform an emotion categorization task (indicate whether the in-group/ out-group member's posture was happy or angry) or a shape categorization task (indicate whether a circle or square was superimposed on in-group/ out-group member's body) |
| Xu, X. et al. (Caucasian Group) | 2009 | 16 | 16/8 | 23/3.7 | Caucasian vs. Chinese | Caucasian | Participants viewed in-group/ out-group member receiving painful (needle penetration) or non-painful (Q-tip touch) stimulation and judged whether or not the model was feeling pain |
| Xu, X. et al. (Chinese Group) | 2009 | 17 | 17/9 | 23/2 | Caucasian vs. Chinese | Chinese |  |
| Zuo, S. et al. | 2013 | 20 | 20/12 | 23.3/3.39 | Chinese vs. Caucasian | Chinese | Participants viewed in-group/ out-group member receiving painful (needle penetration) or non-painful (Q-tip touch) stimulation and judged whether or not the model was feeling pain |
| **Face Processing** | | | | | | | |
| Zacharopoulos, G. et al. | 2023 | 20 | 20/10 | 22.6 | Cardiff University vs. Swansea University | Cardiff University | Participants compared the beauty of two women either from their ingroup or from an outgroup |
| Carollo, A. et al. | 2023 | 43 | 43/22 | 23.36/1.41 | Chinese vs. Indian/ Caucasian/ Arabic | Chinese | Participants passively viewed faces of typical in-group faces (Chinese), typical out-group faces (Indian), or non-typical out-group faces (Caucasian/ Arabic) |
| Cassidy, B. S. et al. | 2021 | 75 | 75/47 | 46.62/4.45 | White vs. Black | White | Participants viewed images of in-group and out-group faces |
| Contreras, J. M. et al. | 2013 | 17 | 17/9 | 22.18 | White vs. Black | White | Participants categorized in-group/ out-group faces by race (Black, White) |
| Cunningham, W. A. et al. | 2004 | 13 | 13/4 | 27 | White vs. Black | White | Participants pressed one of two buttons with their right hand to indicate whether faces of in-group/ out-group members appeared to the left or right of a fixation cross |
| Ebner, N. C. et al. | 2013 | 62 | 30/16 (Young Adults)  32/18 (Older Adults) | 25.1/3.4 (Young Adults)  68.2/2.5 (Older Adults) | Own-age vs. Other-age | Own-age/ Other-age | Participants performed Facial Expression Identification Task - indicate whether in-group/ out-group faces displayed a happy, neutral, or angry expression |
| Farmer, H. et al. | 2020 | 25 | 25/17 | 25.16/4.56 | White vs. Black | White | Participants viewed faces of in-group/ out-group members and categorised them on the basis of age (over or under 25 years old) or their favourite vegetables (as taught in the learning task) |
| Feng, L. et al. | 2011 | 30 | 30/11 | 23 | Chinese vs. Caucasian | Chinese | Participants viewed faces of in-group/ out-group members and categorised them based on race or the degree of similarity with a previously presented face |
| Kaplan, J. T. et al. | 2007 | 20 | 20/10 | 35.7/6.34 (Democrats)  35.6/9.29 (Republicans) | Democrats vs. Republicans | Democrat/ Republican | Participants viewed images of their supporting (in-group)/ opposing (out-group) presidential candidates (George Bush/ John Kerry) |
| Krendl, A. C. et al. | 2009 | 65 | 42/19 (Older Adults)  23/12 (Young Adults) | 73.14 (Older Adults)  19.53 (Young Adults) | Stigmatised Individuals (e.g., individuals with amputations) vs. Typical | Typical | Participants viewed images of stigmatised (out-group, e.g., persons with amputations/ deformities) and non-stigmatised (in-group, control, e.g., persons with no visible stigma) members |
| Lee, K. U. et al. | 2008 | 13 | 13/0 | 24.8/3.6 | Korean vs. Caucasian | Korean | Participants performed a sex discrimination task to elucidate incidental processing of different race emotional faces - press a button when male in-group (White)/ out-group (Black) members were shown on the screen |
| Li, T. et al. | 2016 | 44 | 44/24 | 24.4/4.32 | White vs. Black | White | Participants performed an impression formation task in which they formed impressions of out-group (Black) and in-group (White) members' faces paired with either positive or negative words |
| Lieberman, M. D. et al. | 2005 | 20 | 11/11 (Caucasian-American)  9/9 (African-American) | 23.7 (Caucasian-American)  24.9 (African-American) | African-American vs Caucasian-American | African-American/ Caucasian-American | Participants performed a perceptual encoding task (view and select a face that matched in-group/ out-group target's in terms of race) and verbal encoding task (select the race label that described the race of the in-group/ out-group target's face) |
| Losin, E. A. R. et al. | 2012 | 20 | 20/10 | 22.92/2.09 | European American vs. African American/ Han Chinese | European American | Participants passively observed still portraits of in-group/ out-group members |
| Mattan, B. D. et al. | 2018 | 60 | 60/0 | 23.8/4.59 | White vs. Black | White | Participants performed an impression-formation task - viewed faces of in-group/ out-group members with varying SES |
| McCutcheon, R. et al. (Black Group) | 2018 | 17 | 17/10 (Blacks) | 24.5 (Blacks) | White vs. Black | Black | Participants viewed faces of in-group/ out-group members |
| McCutcheon, R. et al. (White Group) | 2018 | 19 | 19/10 (Whites) | 24.0 (Whites) | White vs. Black | White |  |
| Raghunath, B. L. et al. | 2022 | 27 | 27/16 | 24.91/4.93 | Chinese vs. Indian | Chinese | Participants viewed photos of in-group/ out-group infant faces |
| Richeson, J. A. et al. | 2003 | 15 | 15/8 | 20.5 | White vs. Black | White | Participants viewed images of in-group/ out-group members faces |
| Ronquillo, J. et al. | 2007 | 11 | 11/0 | not provided | White vs. Black | White | Participants categorised dark/light-toned faces of in-group/ out-group members to older or younger than 24 |
| Rubien-Thomas, E. et al. | 2021 | 106 | 106/57 | 26.08 | White vs. Black | White/ Black | Participants performed go/no-go task - press a button as quickly as possible for in-groups ("Only press to male faces") and withhold a response for out-groups |
| Rule, N. O. et al. | 2010 | 28 | 14/14 (Americans)  14/14 (Japanese) | not provided | American vs. Japanese | American/ Japanese | Participants viewed faces of in-group/ out-group members and indicated via button-press whether they would or would not vote for each person |
| Scheepers, D. et al. | 2013 | 41 | 41/0 | 21 | Leiden University vs. VU University Amsterdam | Leiden University | Participants observed pictures of an in-group or out-group member |
| Steines, M. et al. | 2020 | 22 | 22/14 | 26/4.6 | German vs. Turkish  Minimal: Problem solver type (conclusive vs. sequential) | German  Minimal: experimentally assigned | Participants viewed pictures of in-group/ out-group members facial emotions (anger/ neutral) |
| Van Bavel, J. J. et al. | 2008 | 17 | 17/10 | not provided | White vs. Black  Minimal: Team Leopards/ Tigers | White  Minimal: experimentally assigned | Participants viewed and categorized faces of in-group/ out-group members according to team membership or race |
| Yan, Z. et al. | 2019 | 44 | 20/9 (Chinese)  24/12 (German) | 26.02/2.82 (Chinese)  25.38/5.44 (German) | Chinese vs. German  Minimal: Team Green vs. Magenta | Chinese/ German  Minimal: experimentally assigned | Participants viewed and categorized pictures of in-group/ out-group faces according to the corresponding group previously taught prior to fMRI scanning |
| **Decision-making** | | | | | | | |
| Fang, Z. et al. | 2024 | 26 | 25/9 | 21.62 | League of Legends vs. Honor of Kings Players | League of Legends Players | Participants carried out a dot estimation task with another partner (in-group/ out-group) and received feedback about their performance in the task in relation to in-group/ out-group member |
| Baumgartner, T. et al. | 2012 | 16 | 16/0 | 24.5/2.2 | Own Platoon vs. Other Platoon | Own Platoon | Participants adopted the role of a third-party in a Prisoners' Dilemma Game confronted with decisions of in-group/ out-group members and assigned punishment points to them |
| Bruneau, E. G. et al. (Arab Group) | 2010 | 16 | 16/16 (Non-Israeli Arab) | 21.5 (Non-Israeli Arab) | Israeli vs Arab | Arab | Participants read statements (e.g., information about Israeli/ Arabic society/ history/ economy) about in-group or out-group members and rated the reasonableness of each statements on a button press |
| Bruneau, E. G. et al. (Israeli Group) | 2010 | 16 | 16/16 (Jewish Israeli) | 29.0 (Jewish Israeli) | Israeli vs Arab | Israeli |  |
| Cui, F. et al. | 2023 | 28 | 28/14 | 21.3/2.1 | Shenzhen University vs. Different University (not specified) | Shenzhen University | Participants first observe the confederate (in-group or out-group member) and how much Monetary Units they were assigned that trial, which were either scarce (not enough for two players to escape physical threat) or abundant (enough for two players to escape physical threat), and then decides how much Monetary Units to share to reduce a physical threat (unpleasant noise). |
| Domínguez, D, J. F. et al. | 2018 | 48 | 48/35 | 25.3/8.92 | Muslim vs. Non-Muslim (Caucasian) | Non-Muslim (Caucasian) | Participants decided whether or not to shoot an in-group/ out-group member depending on what object (e.g., gun) they were holding |
| Feng, C. et al. (Mortality Salience Group) | 2017 | 20 | 20/20 | 21.2/2.7 | Experimentally assigned: Yellow vs. Red Group  Chinese vs. Korean | Yellow/ Red  Chinese | Participants decided how to punish in-group/ out-group members (after viewing them offering fair/ unfair monetary proposals) by reducing their payoffs |
| Feng, C. et al. (Neutral Group) | 2017 | 20 | 20/20 | 22.1/2.6 | Experimentally assigned: Yellow vs. Red Group  Chinese vs. Korean | Yellow/ Red  Chinese |  |
| Freeman, J. B. et al. | 2010 | 16 | 16/8 | not provided | White vs. Black | White | Participants read statements (superficial/ individuated) about the personality of in-group/ out-group members and judged by a button press whether those statements were true or false |
| Gilbert, S. J. et al. | 2012 | 16 | 16/16 | not provided | White vs. Black | White | Participants viewed a pair of in-group/ out-group faces and judged them according to which in-group/ out-group member is more likely to enjoy athletic activities (trait judgement) and which in-group/ out-group member is more likely to befriend (friendship judgement) |
| Jiang, X. et al. | 2018 | 25 | 25/25 | not provided | Canadian vs. American/French speakers | Canadian | Participants listened to audio recordings of English speakers and judged the extent to which they believe the statements were produced by in-group (native) or out-group (foreign/ regional) speakers |
| Krosch, A. R. et al. | 2019 | 30 | 30/16 | 19.63/1.40 | White vs. Black | White | Participants viewed in-group/ out-group faces and allocated monetary resources to them in scarce (assigned $10 to allocate, but was informed they had up to $100 to allocate) and neutral (up to $10 to allocate) trials |
| Lau, T. et al. | 2017 | 22 | 22/12 | 25.25/2.77 | Political Party Affiliation: Democrats vs. Republicans  Experimentally Assigned Group: Eagles vs. Rattlers | Democrats  Eagles | Participants performed repetition suppression and enhancement task - read paired statements (e.g., "Sam is a Democrat" and "Y is an Eagle") about two targets and asked to indicate if they belong to one of three conditions: ‘identical in-group’ (Democrat-Democrat or Eagles-Eagles), ‘different in-group’ (Eagles-Democrat or Democrat-Eagles), and ‘out-group/in-group trials’ (Republican-Democrat or Rattler-Eagles) |
| Lin, L. C. et al. | 2018 | 45 | 45/24 | 19.42/0.63 | American vs. Chinese | American/ Chinese | Participants performed a social influence task across three phases in which they (1) rated a set of image, (2) viewed how in-group and out-group members rated a subset of the images they had rated, and then (3) rated the images again themselves |
| Littlefield, M. M. et al. | 2015 | 23 | 23/18 | not provided | Experimentally assigned Blue vs. Purple group | Blue/ Purple Group | Participants performed a truth telling task in which they viewed faces of in-group/ out-group members and responded to questions that concern telling a social truth (e.g., is Person X a poor singer?) or a simple truth (e.g., does Person X have brown hair?) |
| Mitchell, G. P. et al. | 2006 | 15 | 15/6 | 24.4 | Liberal vs. Conservative | Liberal/ Conservative | Participants judged how likely in-group/ out-group members were to agree with several opinion questions relating to personal (e.g., to look forward to going home for Thanksgiving?) and societal issues (e.g., to believe that cultural diversity should be an important national issue?) |
| Molenberghs, P. et al. | 2016 | 48 | 48/24 | 22.2/5.3 | UQ students vs. QUT students | UQ | Participants passively watched a video of in-group/ out-group member (perpetrator) intentionally harming another in-group/ out-group member (victim) |
| Molenberghs, P., Bosworth, R. et al. | 2014 | 48 | 48/24 | 22.5/4.9 | Experimentally assigned Red vs. Green team | Red/ Green team | Participants performed a moral evaluation task where they allocated rewards (i.e., money when in-group/ out-group member respond correctly to a question posed) and punishments (i.e., shocks when in-group/out-group did not respond correctly to a question posed) to in-group/ out-group members |
| Molenberghs, P., Morrison, S. et al. | 2014 | 20 | 20/14 | 23/4 | Experimentally assigned Red vs. Blue team | Red/ Blue team | Participants categorized Red Team and Blue Team words as My Team (in-group) and Other Team (out-group) by pressing a left or right button to indicate the side of the matching stimulus |
| Nugiel, T. et al. | 2020 | 50 | 50/36 | 22.18/4.70 | Political Affiliation: Democratic vs. Republican | Democratic | Participants judged (Yes/ No) whether in-group/ out-group members possessed various positive (e.g., Does NOBLE describe Barack Obama?) and/or negative (e.g., Does PHONY describe Barack Obama?) personality traits |
| Telzer, E. H. et al. | 2015 | 26 | 13/7 (American)  13/6 (Chinese) | 19.02 (American)  19.38 (Chinese) | American vs. Chinese | American/ Chinese | Participants performed a prosocial task (modified dictator game) in which they were presented with financial offers and were asked to keep the money for themselves or donate a sum of money to in-group/ out-group members |
| van Gils, S. et al. | 2020 | 17 | 17/17 | not provided | Dutch university vs. German university | Dutch university | Participants were presented with a trolley dilemma situation, and asked to either sacrifice (a) an out-group member, to save a group of in-group members, or (b) an in-group member, to save a group of out-group members |
| Volz, K. G. et al. | 2009 | 20 | 20/10 | 25.3/2.5 | Experimentally assigned Yellow or Blue | Yellow/ Blue Group | Participants assigned money to different in-group/ out-group members |
| Wu, C. T. et al. | 2018 | 54 | 27/16 (KMT)  27/18 (DDP) | 23.30/0.67 (KMT)  24.22/4.60 (DDP) | KMT Candidate voters vs. DPP Candidate voters | KMT/ DDP | Participants played a binary trust game - decide whether or not to invest monetary units (MU) to in-group/ out-group members and participants can decide to reciprocate (to split the money equally so that in-group/out-group member and the participant each receives 20 MU) or defect (keep the entire amount of 40 MU and in-group/out-group member receives 0 MU). |
| **Implicit Processing** | | | | | | | |
| Mauchaund, M. et al. | 2023 | 24 | 24/13 | 26.04/3.99 | French vs. Quebecois | French | Participants listened to short utterances describing a painful event, which were either produced in a neutral-sounding or complaining voice by both in-group (French) and out-group (French Canadian) speakers |
| Azevedo, R. T. et al. | 2013 | 27 | 14/7 (White Caucasian)  13/9 (Black Africans) | 23.57/4.01 (White Caucasian)  24.26/4.35 (Black Africans) | White vs. Black | White-Caucasian/ Black-African | Participants observed right male hands of racial in-groups/ out-groups being either deeply penetrated by a hypodermic needle (pain condition) or touched by a Q‐tip (touch condition) |
| Bestelmeyer, P. E. et al. | 2015 | 40 | 20/11 (Scottish)  20/8 (Southern English) | 23.45/3.62 (Scottish)  18.80/1.44 (Southern English) | English vs. Scottish | English/ Scottish | Participants listened passively to accents of in-group/ out-group members |
| Brown, T. I. et al. | 2017 | 19 | 19/0 | 23.26/4.69 | African American/ European American | African American/ European American | Participants intentionally encoded faces of in-group/ out-group members by generating imaginative stories involving in-group/ out-group members pictured in the stimuli in a manner that would facilitate their later retrieval of the faces the following day |
| Firat, R. B. et al. | 2017 | 13 | 13/6 | 47.2/7.6 | White vs. Black | Whites | Participants viewed pictures of in-group/ out-group members in different socio-economic positions [lower class (e.g., homeless people); middle class (e.g., people barbecuing); upper class (e.g., people wearing upscale clothing and jewellery)] with one of eight primary emotions expressed (e.g., happy, pride, sad) |
| Izuma, K. et al. | 2019 | 70 | 70/27 | 18.9/1.11 | South Korea vs. Japan | Japanese | Participants viewed images related to in-group/ out-group (e.g., people/ national flags) [Note: The fMRI task comprises both face processing and the processing of stimuli unrelated to face, such as flags] |
| Kang, P. et al. | 2021 | 29 | 29/19 | 22.51/0.54 | Left-wing vs. Right-wing political attitudes | Left-wing | Participants performed an observational learning task - learn about the reward probability of two fractal images from in-group/ out-group demonstrators by viewing their actions only (action-based learning) or their actions and the outcomes associated with them (outcome-based learning) |
| Li, T. et al. | 2016 | 44 | 44/24 | 24.4/4.32 | White vs. Black | White | Participants performed an impression formation task in which they formed impressions of out-group (Black) and in-group (White) members' faces paired with either positive or negative words |
| Losin, E. A. R. et al. | 2012 | 20 | 20/10 | 22.92/2.09 | European American vs. African American/ Han Chinese | European American | Participants passively observed still portraits of in-group/ out-group members |
| Molapour, T. et al. | 2015 | 20 | 20/10 | 22.39/3.82 | White vs. Black | White | Participants were classically conditioned with electric shocks across three phases: habituation (view images of in-group/ out-group), acquisition (receive shocks when viewing in-group/ out-group), and extinction (view images of in-group/ out-group without shocks) |
| Molenberghs, P. et al. | 2013 | 24 | 24/0 | 23.8 | Experimentally assigned Red vs. Blue team | Red/ Blue team | Participants passively viewed in-group team and out-group team members perform hand actions |
| Molenberghs, P. et al. | 2017 | 40 | 40/20 | 20.6 | Political Affiliation: Liberals vs. Labor | Liberals/ Labors | Participants were presented with inspirational collective-oriented (e.g., "We will work together and shape a future of indiscriminate prosperity") and non-inspirational personal-oriented statements by in-group/ out-group political members |
| Moradi, Z. et al. | 2017 | 20 | 20/1 | 31/7.50 | Favourite football team vs. Rival team | Favourite football team | Participants performed associative learning task - learn the association between arbitrary geometric shapes and the badges of in-group/ out-group teams in match (both shape/ badge belong to the same in-group/ out-group) and unmatched (shape/ badge deviates from the same group) trials |
| Morrison, S. et al. | 2012 | 20 | 20/14 | 22.9 | Participants' choice of 7 in-groups (any category, e.g., Gender, Nationality, Religious Affiliations, etc.) | Participants' choice of 7 in-groups | Participants categorized words on the basis of whether they belong to in-group (my team) or out-group (other team) |
| Newman-Norlund, R. D. et al. | 2008 | 22 | 22/1 | 24 | Netherlands vs. Germany | Netherlands/ Germany | Participants viewed clips of penalty kicks for in-group/ out-group football teams and asked to judge if it was a goal or miss |
| Richins, M. T. et al. | 2019 | 69 | 69/42 | 20.57/3.04 | University of Exeter vs. Cardiff University vs. University of Sussex | Exeter students | Participants viewed photos of individuals that were denoted as either in-group members or out-group members experiencing painful or innocuous events |
| Ruckmann, J. et al. | 2015 | 30 | 30/15 | 24.50/3.36 | Experimentally assigned problem solver type (conclusive vs. sequential) | Experimentally assigned problem solver type (conclusive vs. sequential) | Participants viewed photos showing in-group/ out-group members' right hands and feet in painful and neutral everyday situations |

# Table S1.6

*Additional descriptive information of included studies*

| **Author(s)** | **Year** | **Study Country** | **Smoothing Kernel (mm)** | **Analyzing software package for fMRI** | **Magnetic field strength (T)** | **In-group contrast(s) used** | **Out-group contrast(s) used** |
| --- | --- | --- | --- | --- | --- | --- | --- |
| Mei, S. et al. | 2025 | China | 4 | SPM12 | 3 | In-Group > Out-Group for Pain (> Non-Pain) Stimulations | - |
| Fang, Z. et al. | 2024 | China | 8 | SPM12 | 3 | In-group > Out-group* | Out-group > In-group* |
| Mauchaund, M. et al. | 2023 | Canada | 5 | SPM12 | 3 | In-Group > Out-Group  In-group (Complaining > Neutral) > Out-group (Complaining > Neutral)* | Out-group > In-group  Out-group (Complaining > Neutral) > In-group (Complaining > Neutral) |
| Zacharopoulos, G. et al. | 2023 | United Kingdom | 8 | SPM12 | 3 | In-Group > Out-Group* | - |
| Azevedo, R. T. et al. | 2013 | Italy | 8 | SPM8 | 3 | Main effect of in‐group (i.e., in‐group stimuli > out-group stimuli) [not differentiated between pain and touch stimuli]  Observing in-group members in pain > out-group members in pain | - |
| Baumgartner, T. et al. | 2012 | Switzerland | 8 | SPM5 | 3 | Observing punishment of in-group > punishment of out-group (in-group effects for punishment network)  Mentalizing decisions of in-group > out-group (in-group effects for mentalization network) | Observing punishment of out-group > punishment of in-group (outgroup effects for punishment network)  Mentalizing decisions of out-group > in-group (out-group effects for mentalization network) |
| Berlingeri, M. et al. | 2016 | Italy | 8 | SPM8 | 1.5 | In-group differential empathic activation for race effect during Stimulus phase (painful > neutral) | Out-group differential empathic activation for race during Response phase (painful > neutral) |
| Bestelmeyer, P. E. et al. | 2015 | Scotland | 8 | SPM8 | 3 | In-group > out-group accent | - |
| Brown, T. I. et al. | 2017 | United States | 8 | SPM8 | 3 | Memory encoding-related activity for same- (in-group) > other-race (out-group) | - |
| Bruneau, E. G. et al. | 2012 | United States | 5 | SPM8, SnPM5, and In-house | 3 | - | Emotional pain of conflict out-group > in-group member (Arab > Israeli) |
| Bruneau, E. G. et al. | 2010 | United States | 5 | SPM2 | 3 | Among Arab participants: pro-Arab (in-group) > pro-Israel (out-group) statements  Among Israeli participants: pro-Israel (in-group) > pro-Arab (out-group) statements | Among Arab participants: pro-Israel (out-group) > pro-Arab (in-group) statements  Among Israeli participants: pro-Arab (out-group) > pro-Israel (in-group) statements |
| Cao, Y. et al. | 2015 | China | 6 | SPM8 | 3 | Observing Chinese (in-group) in pain > observing Caucasian (out-group) in pain (interaction effects comparing neural empathic activation to Chinese > Caucasian faces)  Observing in-group in pain > observing out-group in pain* | - |
| Carollo, A. et al. | 2023 | Singapore | 8 | SPM12 | 3 | Viewing Chinese (in-group) faces > Arabic + Indian + Caucasian (out-groups) faces  Viewing Chinese (in-group) faces > Indian (out-group) faces  Viewing Chinese (in-group) faces > Arabic (out-group) faces | - |
| Cassidy, B. S. et al. | 2021 | United States | 8 | SPM12 | 3 | Main effect of target race (viewing in-group faces > out-group)* | Main effect of target race (viewing out-group faces > in-group)* |
| Chen, P. A. et al. | 2015 | United States | 6 | SPM8 | 3 | Main effect of Group: viewing in-group emotional faces > viewing out-group emotional faces | - |
| Cheon, B. K. et al. | 2011 | South Korea & United States | 8 | SPM2 | 3 | In-group bias in empathy  Ingroup Bias in Empathy for Caucasian > Korean  Ingroup Bias in Empathy for Korean > Caucasian | Outgroup Bias in empathy [(Outgroup Pain > Outgroup Neutral) > (Ingroup Pain > Ingroup Neutral)] |
| Chiao, J. Y. et al. | 2008 | Japan & US | 8 | SPM99 | 3 | Own-culture Fear > Other-culture Fear | - |
| Contreras-Huerta, L. S. et al. | 2013 | Australia | 6 | SPM8 | 3 | Stimuli x Race interaction (in-group bias): observed painful > non-painful touch in actors of the same race (Caucasian, in-group) > actors of the other race (Chinese, out-group) | - |
| Contreras, J. M. et al. | 2013 | United States | 8 | SPM8 & In-house | 3 | Main Effect of Race: categorizing White faces > Black faces | Main Effect of Race: categorizing Black faces > White faces |
| Cui, F. et al. | 2023 | China | 6 | SPM12 | 3 | In-group bias under scarcity (allocating scarce resource to in-group > out-group members)  Group membership x resource information interaction [(scarcity-ingroup > scarcity-outgroup) > (abundance-ingroup > abundance-outgroup)] (allocating scarce resource to in-group members > out-group members and abundant resources) | - |
| Cunningham, W. A. et al. | 2004 | United States | 9 | SPM99 | 1.5 | Greater response to White > Black faces (525-ms condition) | Greater response to Black > White faces (30-ms condition)  Greater response to Black > White faces (525-ms condition) |
| Domínguez, D, J. F. et al. | 2018 | Australia | 9 | SPM12 | 3 | Justified shooting decisions towards muslims (out-group) > non-muslims (in-group)*  Unjustified shooting decisions towards muslims (out-group) > non-muslims (in-group)* | - |
| Earls, H. A. et al. | 2013 | United States | 5 | FSL | 3 | Actor race × condition interaction: observing and imitating own-race (Caucasian; in-group) > other-race (African-American; out-group) | - |
| Ebner, N. C. et al. | 2013 | United States | 9 | SPM5 | 3 | Age of Face × Participant Age interaction across facial expressions: (greater activity to own-age > other-age faces for both young and older adults) | - |
| Falk, E. B. et al. | 2012 | United States | 8 | SPM5 | 3 | Taking perspectives of one's own candidate (in-group) > opponent (out-group) | Taking perspective of opponent's candidate (out-group) > one's own candidate (in-group) |
| Farmer, H. et al. | 2020 | England | 8 | SPM12 | 1.5 | viewing White faces (in-group) > viewing Black faces (out-group) | - |
| Feng, C. et al. | 2017 | China | 8 | SPM8 | 3 | Mortality salience priming: punishment of in-group > out-group members*  Control: punishment of in-group > out-group members* | - |
| Feng, L. et al. | 2011 | China | 8 | SPM8 | 3 | Categorizing Chinese (in-group) faces > categorizing Caucasian (out-group) faces | - |
| Firat, R. B. et al. | 2017 | United States | 4 | AFNI | 3 | Experiment 2: viewing middle-class depictions of Whites (in-group) > Blacks (out-group) | - |
| Fourie, M. M. et al. | 2017 | South Africa | not provided | BrainVoyagerQX Version 2.8 | 3 | In-group biases in activation for perceived physical pain, in-group > out-group (Participant Race x Pain x Victim Race)  In-group biases in activation for perceived social pain, in-group > out-group (Participant Race x Distress x Victim Race) | - |
| Fox, G. R. et al. | 2013 | United States | 8 | BrainVoyagerQX & MATLAB (version 2007a) | 3 | - | Processing hateful people (out-group; neo-Nazis) in pain > processing likeable people (in-group) in pain |
| Freeman, J. B. et al. | 2010 | United States | 7 | BrainVoyagerQX | 3 | Judgment × race effect: selectivity for individuated judgments for Whites (in-group) > Blacks (out-group) | - |
| Gilbert, S. J. et al. | 2012 | United States | 4 | SPM8 | 3 | Judgement of White (in-group) > Black (out-group) faces (collapsed over trait and friendship judgements)  Friendship (evaluative) judgments of White (in-group) > Black (out-group) faces  Stereotype-related trait judgement of White (in-group) > Black (out-group) faces" | - |
| Handley, G. et al. | 2023 | United States | 8 | SPM8 | 3 | Responding to White (in-group) Reading the Mind in the Eyes (RME) test > responding Black (out-group) Reading the Mind in the Eyes (RME) test | - |
| Hein, G. et al. | 2010 | Switzerland | 10 | SPM5 | 3 | (high > low in-group pain) > (high > low out-group pain) | - |
| Hein, G. et al. | 2016 | Switzerland | 6 | SPM8 | 3 | Before intervention (control group): responses to in-group > out-group pain  Before intervention (experimental group): responses to in-group > out-group pain | - |
| Izuma, K. et al. | 2019 | Japan | 3 | SPM8 | 8 | - | Viewing images related to South Korea (out-group) > Japan (in-group) |
| Jiang, X. et al. | 2018 | Canada | 5 | FSL | 3 | Main Effect of Accent: In-group > Out-group (Regional + Foreign)  In-group (Confidence>Neutral) > Out-group/Regional (Confident>Neutral) | Main Effect of Accent: Out-group (Regional + Foreign) > In-group  Out-group/Regional (Confident>Neutral) > In-group (Confident>Neutral)  Out-group/Regional (Confident>Doubtful) > In-group (Confident>Doubtful)  Out-group/Foreign (Confident>Neutral) > In-group (Confident>Neutral)  Out-group/Foreign (Confident>Doubtful) > In-group (Confident>Doubtful)  Out-group/Foreign (Doubtful>Neutral) > In-group (Doubtful>Neutral) |
| Kang, P. et al. | 2021 | Switzerland | 8 | SPM12 | 3 | Learning from observing the outcomes and actions of in-group members > out-group members* | - |
| Kaplan, J. T. et al. | 2007 | United States | 5 | FSL | 3 | - | Viewing opposing candidate's (out-group) face > own candidate's (in-group) face |
| Katsumi, Y. et al. | 2018 | United States | 8 | SPM8 | 3 | Main effect of race (perception/ categorization of in-group > out-group affective body postures)* | Main effect of race (perception/ categorization of out-group > in-group affective body postures)* |
| Kim, K. et al. | 2015 | United States | 5 | FSL | 3 | Viewing in-group preference > viewing out-group preferences | - |
| Krendl, A. C. et al. | 2009 | United States | 8 | SPM2 | 3 | - | Viewing stigmatized members' face (out-group) > control face (in-group) |
| Krosch, A. R. et al. | 2019 | United States | 6 | SPM8 | 3 | Main effect of viewing: White (in-group) > Black (out-group) faces | Main effect of viewing: Black (out-group) > White (in-group) faces |
| Lau, T. et al. | 2017 | United States | 5 | SPM8 | 3 | Repetition enhancement: in-group (Democrats/ Eagles) > out-group (Republicans/ Rattlers) | Repetition suppression: out-group (Republicans/ Rattlers) > in-group (Democrats/ Eagles) |
| Lee, K. U. et al. | 2008 | Korea | not provided | SPM2 | 1.5 | Own-race > other-race neutral face  Own-race > other-race emotional face (happy and sad) | Other-race > own-race neutral face  Other-race > own-race emotional face (happy and sad) |
| Li, T. et al. | 2016 | United States | 8 | SPM8 | 3 | Viewing White (in-group) face > viewing Black (out-group) face  Viewing White (in-group) face with positive trait > Black (out-group) face with positive trait | - |
| Li, X. et al. | 2015 | China | 8 | SPM8 | 3 | Mortality salience group: viewing in-group in pain > out-group  Negative affect group: viewing in-group in pain > out-group | - |
| Li, Z. et al. | 2020 | China | 8 | SPM8 | 3 | Group-based guilt: Observe in-group in pain ​> ​out-group | - |
| Lieberman, M. D. et al. | 2005 | United States | 8 | SPM99 | 3 | Viewing in-group face > out-group face | Viewing out-group face > in-group face |
| Lin, L. C. et al. | 2018 | United States | 6 | SPM8 | 3 | Aligning ratings during the social influence task to in-group > out-group members | - |
| Littlefield, M. M. et al. | 2015 | Denmark | 6 | SPM8 | 3 | Main effect of group membership: responding to questions about in-group members > out-group members* | - |
| Liu, Y. et al. | 2015 | China | 6 | SPM8 | 3 | - | Main effect of group: Out-group > In-group  (Viewing disgusted out‐group member > viewing neutral out‐group) > (viewing disgusted in‐group > viewing neutral in‐group) |
| Losin, E. A. et al. | 2012 | United States | 6 | FSL, AFNI, & ART | 3 | Imitate gesture own gender (in-group) > other gender (out-group) | - |
| Losin, E. A. R. et al. | 2012 | United States | 6 | FSL, AFNI, & ART | 3 | Imitate gesture of EA (in-group) > CH (out-group)  Viewing EA portrait (in-group) > CH portrait (out-group)  Viewing EA portrait (in-group) > AA portrait (out-group)  Observe Gesture EA (in-group) > CH (out-group) | Imitate gesture AA (out-group) > EA (in-group) |
| Luo, S. et al. | 2015 | China | 8 | SPM8 | 3 | G/G genotype individuals: Viewing Asian (in-group) > Caucasian (out-group) faces in pain | A/A genotype individuals: Viewing Caucasian (out-group) > Asian (in-group) faces in pain |
| Marsh, L. E. et al. | 2016 | United Kingdom | 12 | SPM12 | 3 | Imitative compatibility toward IG > OG (incompatible > compatible)  Spatial compatibility toward IG > OG (incompatible > compatible)  General compatibility [spatial and imitative compatibility were consistent (both compatible, or both incompatible)] toward IG > OG (incompatible > compatible) | - |
| Mathur, V. A. et al. | 2012 | United States | 8 | SPM2 | 3 | Main effect of racial group: viewing African-American (in-group) in pain > Caucasian-American (out-group) in pain  Main effect of racial group: viewing Caucasian-American (in-group) in pain > African-American (out-group) in pain | - |
| Mathur, V. A. et al. | 2010 | United States | 8 | SPM2 | 3 | African-American: observing in-group in pain > observing out-group in pain  Caucasian-American: observing in-group in pain > observing out-group in pain  Observing in-group in pain and no pain > observing out-group in pain and no pain | Observing out-group in pain and no pain > observing in-group in pain and no pain |
| Mattan, B. D. et al. | 2018 | United States | 8 | SPM8 | 3 | Main effect of race: Viewing White (in-group) face > viewing Black (out-group) face | Main effect of race: Viewing Black (out-group) face > viewing White (in-group) face  Status × Race Interaction |
| McCutcheon, R. et al. | 2018 | United Kingdom | 8 | SPM8 | 3 | Whites: viewing White faces > Black faces* | Blacks: viewing White faces > Black faces |
| Mitchell, G. P. et al. | 2006 | United States | 8 | SPM99 | 1.5 | Judgement of similar others [in-group, Liberal/ Conservative (dependent on participant) target] > judgement of dissimilar others [out-group, Liberal/ Conservative (dependent on participant) target] | Judgement of dissimilar others [out-group, Liberal/ Conservative (dependent on participant) target] > judgement of similar others [in-group, Liberal/ Conservative (dependent on participant) target] |
| Molapour, T. et al. | 2015 | Sweden | 8 | SPM8 | 3 | Acquisition: Overall activity (White > Black)  Extinction: Overall activity (White > Black)  Acquisition: linear change over time: White shock > Black shock | Acquisition: Overall activity (Black > White)  Extinction: Overall activity (Black > White)  Acquisition: linear change over time: Black shock > White shock  Extinction: linear change over time: Black shock > White shock" |
| Molenberghs, P. et al. | 2016 | Australia | 6 | SPM8 | 3 | Main effect of perpetrator's group membership (in-group > out-group)*  Main effect of victim's group membership (in-group > out-group)*  Perpetrator's × victim's group membership (in-group > out-group) interaction* | - |
| Molenberghs, P. et al. | 2013 | Australia | 7 | SPM5 | 1.5 | Passive viewing own team (in-group) gestures > other team (out-group) gestures* | - |
| Molenberghs, P. et al. | 2017 | Australia | 9 | SPM8 | 3 | Main effect of group membership: reading statements from in-group > out-group members* | - |
| Molenberghs, P., Bosworth, R. et al. | 2014 | Australia | 6 | SPM8 | 3 | Rewarding in-group > out-group  Punishing in-group > out-group | - |
| Molenberghs, P., Morrison, S. et al. | 2014 | Australia | 6 | SPM8 | 3 | Categorizing ‘My Team’ > categorizing ‘Other Team’ condition | - |
| Moradi, Z. et al. | 2017 | United Kingdom | 8 | SPM8 | 3 | - | Mismatch trials: out-group > in-group |
| Morrison, S. et al. | 2012 | Australia | 6 | SPM8 | 3 | Viewing words related to in-group > out-group | - |
| Newman-Norlund, R. D. et al. | 2008 | Netherlands | 6 | SPM2 | 3 | Observation of in-group > out-group members  Observation of errors of in-group > out-group members | - |
| Nugiel, T. et al. | 2020 | United States | 8 | FSL | 3 | In-group > out-group evaluation | Out-group > in-group evaluation |
| Raghunath, B. L. et al. | 2022 | Singapore | 3 | SPM12 | 3 | Viewing in-group face > viewing out-group face* | Viewing out-group face > in-group face* |
| Rauchbauer, B. et al. | 2015 | Austria | 8 | SPM8 | 3 | In-group (Incongruent > Congruent) masked inclusively with In-group (Incongruent > Congruent) > Out-group (Incongruent > Congruent) | Out-group (Incongruent > Congruent) masked inclusively with Out-group (Incongruent > Congruent) > In-group (Incongruent > Congruent) |
| Richeson, J. A. et al. | 2003 | United States | 6 | SPM99 | 1.5 | - | Experiment 1: Viewing Black face > viewing White face |
| Richins, M. T. et al. | 2019 | United Kingdom | 5 | FSL | 1.5 | Main effect Target Group: (Exeter > Cardiff) + (Sussex > Cardiff) (not differentiated between pain and no-pain conditions) | - |
| Ronquillo, J. et al. | 2007 | Canada | not provided | BrainVoyagerQX | 3 | Interaction [(viewing White (in-group) face in dark tone > light tone) > (viewing Black (out-group) face in dark tone > light tone)] | Viewing Black (out-group) face > viewing White (in-group) face |
| Rubien-Thomas, E. et al. | 2021 | United States | 5 | FSL | 3 | Stimulus Race by Participant Race Interaction: Black > White Participants in Black > White Faces | - |
| Ruckmann, J. et al. | 2015 | Germany | 8 | SPM8 | 3 | Main effect of condition (in-group > out-group) [not differentiated between pain and no-pain conditions]  Interaction between pain × condition (observing in-group in pain > out-group in pain) | - |
| Rule, N. O. et al. | 2010 | United States | 7 | BrainVoyagerQX | 3 | - | Across ALL participants: Target × participant interaction: observing out-group > in-group faces |
| Scheepers, D. et al. | 2013 | Netherlands | 8 | SPM8 | 3 | Viewing In-group face > viewing Out-group face | - |
| Sheng, F. et al. | 2014 | China | 8 | SPM8 | 3 | Race judgements: Asian (in-group) face (Pain > Neutral) > Caucasian (out-group) face (Pain > Neutral) | - |
| Steines, M. et al. | 2020 | Germany | 8 | SPM12 | 3 | Main effect of ethnic group: viewing ethnic in-group faces > ethnic out-group faces*  Main effect of minimal group: viewing minimal in-group faces > minimal out-group faces* | Main effect of ethnic group: viewing ethnic out-group faces > ethnic in-group faces*  Main effect of minimal group: viewing minimal out-group faces > minimal in-group faces* |
| Telzer, E. H. et al. | 2015 | United States | 8 | SPM8 | 3 | Donating to in-group members > donating to out-group members | - |
| Van Bavel, J. J. et al. | 2008 | not provided | 9 | SPM5 | 3 | Main effect of team (Greater activity in response to novel in-group > novel out-group faces) | Main effect of race: observing Black (out-group) > White (in-group) faces |
| van Gils, S. et al. | 2020 | Netherlands | 8 | BrainVoyagerQX | 3 | - | Sacrifice out-group > in-group |
| Volz, K. G. et al. | 2009 | Germany | 5.65 | LIPSIA | 3 | Assigning money in in-group (mono-chrome matrices in the color of the in-group) > out-group (monochrome matrices in the color of the out-group) trials | - |
| Wang, C. et al. | 2015 | China | 8 | SPM8 | 3 | Priming × Race interaction analysis of the contrast of painful > non-painful stimuli (in-group > out-group) | - |
| Watson, R. et al. | 2017 | Netherlands | 6 | BrainVoyagerQX | 3 | Both Tasks: White (in-group) angry bodies > Black (out-group) angry bodies  Emotion categorization task: White (in-group) bodies > Black (out-group) bodies  Shape categorization task: White (in-group) angry bodies > Black (out-group) angry bodies | Both Tasks: Black (out-group) bodies > viewing White (in-group) bodies  Both Tasks: Black (out-group) happy bodies > White (in-group) happy bodies  Emotion categorization task: Black (out-group) bodies > White (in-group) bodies  Emotion categorization task: Black (out-group) happy bodies > White (in-group) happy bodies  Shape categorization task: Black (out-group) bodies > White (in-group) bodies  Shape categorization task: Black (out-group) happy bodies > White (in-group) happy bodies |
| Wu, C. T. et al. | 2018 | Taiwan | 10 | SPM8 | 3 | Playing Binary trust game with person of the same political identity > different political identity (SAME-DM2 > DIFF-DM2) | - |
| Yan, Z. et al. | 2019 | Germany | 9 | SPM12 | 3 | Categorizing in-ethnicity faces > categorizing out-ethnicity faces*  Categorizing in-team faces > categorizing out-team faces* | - |
| Zuo, S. et al. | 2013 | China | 8 | SPM8 | 3 | Racial in-group bias in empathy (Pain × Race interaction)* | **-** |

*Note:* Contrasts indicated with an asterisk (*) denote that there was no significant brain activation observed for the selected contrasts. Given that the Activation Likelihood Estimation (ALE) procedure assesses spatial convergence among published coordinates (rather than the presence of a real effect), the ALE procedure does not accommodate studies with null findings. These contrasts were excluded from the ALE meta-analysis.

# Table S1.7

*Quality assessment of included studies*

| **Author(s)** | **Year** | **Sample Size** | **Magnetic field strength (T)** | **Analyzing software package for fMRI** | **Smoothing Kernel (mm)** | **Thresholding Methods** | **Quality Assessment Score** |
| --- | --- | --- | --- | --- | --- | --- | --- |
| Azevedo, R. T. (1) | 2013 | 27 | 3 | SPM8 | 8 | voxel level at P < 0.001(uncorrected) and clusters significance set at P < 0.05 (FWE‐corrected) | 6 |
| Azevedo, R. T. (2) | 2013 | 14 | 3 | SPM8 | 8 | voxel level at P < 0.001(uncorrected) and clusters significance set at P < 0.05 (FWE‐corrected) | 4 |
| Baumgartner, T. (1) | 2012 | 16 | 3 | SPM5 | 8 | at P < 0.005, voxel extent threshold: 10 voxels, activity in all regions survives small volume family‐wise‐error (FWE) corrections at P < 0.05, except dorsal caudatus (OG>IG) | 6 |
| Baumgartner, T. (2) | 2012 | 16 | 3 | SPM5 | 8 | at P < 0.005, voxel extent threshold: 10 voxels, activity in all regions survives small volume family‐wise‐error (FWE) corrections at P < 0.05, except dorsal caudatus (OG>IG) | 6 |
| Berlingeri, M. | 2016 | 25 | 1.5 | SPM8 | 8 | p<.05 FWE-corrected voxelwise; p<.05 FDR-corrected voxelwise | 5 |
| Bestelmeyer, P. E. | 2015 | 40 | 3 | SPM8 | 8 | corrected at cluster level (P > 0.05) | 6 |
| Brown, T. I. | 2017 | 19 | 3 | SPM8 | 8 | reduced cluster extent threshold was k = 29 with a voxel-level difference at p < 0.005 | 5 |
| Bruneau, E. G. | 2012 | 24 | 3 | SPM8, SnPM5, and In-house | 5 | Corrected p thresholds, at p < 0.05, based on Monte Carlo simulations of the false positive rate | 5 |
| Bruneau, E. G. (Arab Group) | 2010 | 16 | 3 | SPM2 | 5 | thresholding was done at t > 4.0, p < 0.001 with k > 10 | 5 |
| Bruneau, E. G. (Israeli Group) | 2010 | 16 | 3 | SPM2 | 5 | thresholding was done at t > 4.0, p < 0.001 with k > 10 | 5 |
| Cao, Y. (1) | 2015 | 30 | 3 | SPM8 | 6 | cluster-level threshold of PFWE < .05, corrected for multiple comparisons, with clusters formed by the voxel-level height threshold of Puncorrected < .001 | 7 |
| Cao, Y. (2) | 2015 | 30 | 3 | SPM8 | 6 | cluster-level threshold of PFWE < .05, corrected for multiple comparisons, with clusters formed by the voxel-level height threshold of Puncorrected < .001 | 7 |
| Carollo, A. | 2023 | 43 | 3 | SPM12 | 8 | p < 0.0001 (uncorrected) | 5 |
| Cassidy, B. S. | 2021 | 75 | 3 | SPM12 | 8 | p < .005 (k = 50) | 6 |
| Chen, P. A. | 2015 | 22 | 3 | SPM8 | 6 | uncorrected threshold at P < 0.001 for a whole-brain correction of P < 0.05, which was 44 continuous voxels | 5 |
| Cheon, B. K. | 2011 | 27 | 3 | SPM2 | 8 | < 0.005, extant threshold = 10 voxels | 6 |
| Chiao, J. Y. | 2008 | 20 | 3 | SPM99 | 8 | p < .001, Extant Threshold = 10 Voxels | 5 |
| Contreras-Huerta, L. S. (1) | 2013 | 20 | 3 | SPM8 | 6 | a cluster-level probability threshold of PFWE<0.05, with clusters defined by the voxel-level threshold Puncorrected<0.001 | 6 |
| Contreras-Huerta, L. S. (2) | 2013 | 20 | 3 | SPM8 | 6 | a cluster-level probability threshold of PFWE<0.05, with clusters defined by the voxel-level threshold Puncorrected<0.001 | 6 |
| Contreras, J. M. | 2013 | 17 | 3 | SPM8 & In-house | 8 | cluster-extent corrected (Monte Carlo, p < .005 voxelwise + 75 voxels) | 5 |
| Cui, F. | 2023 | 28 | 3 | SPM12 | 6 | p < .001 uncorrected, k > 20, at the voxel level and to an extent threshold of p < .05 with family-wise error (FDR) correction at the cluster level | 7 |
| Cunningham, W. A. | 2004 | 13 | 1.5 | SPM99 | 9 | 13 contiguous voxels differed for Black and White faces at a significance level greater than p<.005 (t>3.05) | 3 |
| Domínguez, D, J. F. | 2018 | 48 | 3 | SPM12 | 9 | cluster extent threshold of 111 contiguous voxels, p<.05 corrected | 7 |
| Earls, H. A. | 2013 | 20 | 3 | FSL | 5 | false discovery rate of 0.05 | 6 |
| Ebner, N. C. | 2013 | 62 | 3 | SPM5 | 9 | each at p < .001 (Only clusters > 2 voxels are reported) | 6 |
| Falk, E. B. | 2012 | 23 | 3 | SPM5 | 8 | voxel-wise threshold of p < 0.005, k = 45, corresponding to false discovery rate (FDR)-corrected p < 0.05 based on a Monte Carlo simulation implemented using AlphaSim in the software package AFNI | 6 |
| Farmer, H. | 2020 | 25 | 1.5 | SPM12 | 8 | voxel level FWE corrected significance of 0.05 | 4 |
| Feng, C. (Mortality Salience Group) | 2017 | 20 | 3 | SPM8 | 8 | Voxel‐wise q(FDR) < 0.01 in conjunction with cluster size >= 20 voxels | 6 |
| Feng, C. (Mortality Salience Group) | 2017 | 20 | 3 | SPM8 | 8 | Voxel‐wise q(FDR) < 0.01 in conjunction with cluster size >= 20 voxels | 6 |
| Feng, C. (Neutral Group) | 2017 | 20 | 3 | SPM8 | 8 | Voxel‐wise q(FDR) < 0.01 in conjunction with cluster size >= 20 voxels | 6 |
| Feng, C. (Neutral Group) | 2017 | 20 | 3 | SPM8 | 8 | Voxel‐wise q(FDR) < 0.01 in conjunction with cluster size >= 20 voxels | 6 |
| Feng, L. | 2011 | 30 | 3 | SPM8 | 8 | uncorrected threshold of p < .0001 with an extent threshold of 30 voxels | 5 |
| Firat, R. B. | 2017 | 13 | 3 | AFNI | 4 | corrected cluster-wise activation threshold of P < 0.05, the minimum cluster sizes of 37 and 24 should be considered for voxel-wise thresholds of P < 0.01, P < 0.005, respectively | 6 |
| Fourie, M. M. | 2017 | 38 | 3 | Brain Voyager QX version 2.8 | not provided | cluster-level thresholding at an uncorrected p < .05 | 5 |
| Fox, G. R. | 2013 | 16 | 3 | Brain Voyager & MATLAB (version 2007a) | 8 | false discovery rate (FDR) of p < 0.05 | 6 |
| Freeman, J. B. | 2010 | 16 | 3 | BrainVoyagerQX | 7 | P < 0.0005 (for the judgment × race analysis) and a cluster extent of at least 10 contiguous functional voxels. | 5 |
| Gilbert, S. J. | 2012 | 16 | 3 | SPM8 | 4 | uncorrected threshold of p<.005, in conjunction with an extent threshold determined by SPM8 to yield a family-wise-error corrected whole-brain threshold of p<.05 | 6 |
| Handley, G. | 2023 | 58 | 3 | SPM8 | 8 | uncorrected voxel-level threshold of p ⟨0.001 and a cluster extent threshold of 53 voxels as determined by AlphaSim | 6 |
| Hein, G. | 2010 | 16 | 3 | SPM5 | 10 | P < 0.001 uncorrected; P < 0.05 whole brain FDR corrected | 5 |
| Hein, G. (Control Group) | 2016 | 18 | 3 | SPM8 | 6 | uncorrected, P < 0.001, k = 5 | 5 |
| Hein, G. (Experimental Group) | 2016 | 20 | 3 | SPM8 | 6 | uncorrected, P < 0.001, k = 5 | 5 |
| Izuma, K. | 2019 | 70 | 8 | SPM8 | 3 | p < 0.001 voxelwise (uncorrected) and cluster p < 0.05 (FWE corrected for multiple comparisons) | 8 |
| Jiang, X. | 2018 | 25 | 3 | FSL | 5 | cluster-level z > 1.96, p < .05 (GRF corrected) | 6 |
| Kang, P. | 2021 | 29 | 3 | SPM12 | 8 | p<0.05, family wise cluster-level whole brain corrected with a cluster inducing voxel-level threshold of p<0.001 | 7 |
| Kaplan, J. T. | 2007 | 20 | 3 | FSL | 5 | clusters determined by Z > 2.3 and a (corrected) cluster significance threshold of p = .05 | 5 |
| Katsumi, Y. | 2018 | 20 | 3 | SPM8 | 8 | height threshold of p < 0.05 corrected for false discovery rate | 6 |
| Kim, K. | 2015 | 24 | 3 | FSL | 5 | voxels were thresholded at an entry level of Z > 2.3 and the significance of the resulting cluster was then evaluated at a cluster probability P < 0.05 using a Gaussian random field theory approach to correct for multiple comparisons | 5 |
| Krendl, A. C. | 2009 | 65 | 3 | SPM2 | 8 | p < .001, uncorrected, with five-voxel extent threshold | 6 |
| Krosch, A. R. | 2019 | 30 | 3 | SPM8 | 6 | p < .005, k > 20 | 6 |
| Lau, T. | 2017 | 22 | 3 | SPM8 | 5 | minimum cluster size of 365 voxels to achieve corrected p < 0.001 whole-brain contrasts, with a voxelwise threshold of p < 0.005 | 5 |
| Lau, T. | 2017 | 22 | 3 | SPM8 | 5 | minimum cluster size of 365 voxels to achieve corrected p < 0.001 whole-brain contrasts, with a voxelwise threshold of p < 0.005 | 5 |
| Lee, K. U. (1) | 2008 | 13 | 1.5 | SPM2 | not provided | five contiguous voxels and a P value less than 0.005 uncorrected (t>3.05) | 3 |
| Lee, K. U. (2) | 2008 | 13 | 1.5 | SPM2 | not provided | five contiguous voxels and a P value less than 0.005 uncorrected (t>3.05) | 3 |
| Li, T. (1) | 2016 | 44 | 3 | SPM8 | 8 | threshold = p < .001, uncorrected; clusters >/= 66 voxels determined by AlphaSim | 6 |
| Li, T. (2) | 2016 | 44 | 3 | SPM8 | 8 | threshold = p < .001, uncorrected; clusters >/= 66 voxels determined by AlphaSim | 6 |
| Li, X. (Experiment 2; Mortality Salience Group) | 2015 | 20 | 3 | SPM8 | 8 | p < 0.05 corrected for multiple comparisons based on a combined voxelwise and cluster-size threshold (p < 0.05, k = 32) | 5 |
| Li, X. (Experiment 2; Negative Affect Group) | 2015 | 20 | 3 | SPM8 | 8 | p < 0.05 corrected for multiple comparisons based on a combined voxelwise and cluster-size threshold (p < 0.05, k = 32) | 5 |
| Li, Z. | 2020 | 31 | 3 | SPM8 | 8 | voxel-wise P < 0.005, minimum cluster extent = 46 voxels | 6 |
| Lieberman, M. D. | 2005 | 20 | 3 | SPM99 | 8 | threshold of P <.005 combined with a cluster size threshold of 5 voxels – corresponding to a corrected threshold of P<.05 | 5 |
| Lin, L. C. | 2018 | 45 | 3 | SPM8 | 6 | voxel-wise threshold of P < 0.005 in combination with a minimum cluster size of 231 voxels for the one-sample t tests and 226 voxels for the two-sample t tests, corresponding to P < 0.05, family-wise error corrected | 7 |
| Littlefield, M. M. | 2015 | 23 | 3 | SPM8 | 6 | p < 0.05, family-wise error (FWE) whole-brain corrected for multiple comparisons | 6 |
| Liu, Y. | 2015 | 26 | 3 | SPM8 | 6 | height threshold of P < 0.01 and an extent threshold of P < 0.05, with family‐wise error corrections for multiple comparisons based on nonstationary suprathreshold cluster‐size distributions computed using Monte Carlo simulations | 7 |
| Losin, E. A. | 2012 | 19 | 3 | FSL, AFNI, & ART | 6 | statistical threshold of Z > 2.3, whole-brain corrected for multiple comparions (P < 0.05) | 5 |
| Losin, E. A. R. (1) | 2012 | 20 | 3 | FSL, AFNI, & ART | 6 | thresholded at z > 2.3 corrected for multiple comparisons using cluster-based Gaussian random field theory controlling familywise error across the whole-brain at p = 0.05 | 6 |
| Losin, E. A. R. (2) | 2012 | 20 | 3 | FSL, AFNI, & ART | 6 | thresholded at z > 2.3 corrected for multiple comparisons using cluster-based Gaussian random field theory controlling familywise error across the whole-brain at p = 0.05 | 6 |
| Losin, E. A. R. (3) | 2012 | 20 | 3 | FSL, AFNI, & ART | 6 | thresholded at z > 2.3 corrected for multiple comparisons using cluster-based Gaussian random field theory controlling familywise error across the whole-brain at p = 0.05 | 6 |
| Luo, S. (A/A Genotype Group) | 2015 | 30 | 3 | SPM8 | 8 | voxel threshold: p = 0.001; Cluster threshold: p =0.05 FWE corrected | 7 |
| Luo, S. (G/G Genotype Group) | 2015 | 30 | 3 | SPM8 | 8 | voxel threshold: p = 0.001; Cluster threshold: p =0.05 FWE corrected | 7 |
| Marsh, L. E. | 2016 | 24 | 3 | SPM12 | 12 | voxel level threshold of p = 0.001 (uncorrected) with cluster level correction (p = 0.05 FWE). | 6 |
| Mathur, V. A. (African-American Group) | 2012 | 10 | 3 | SPM2 | 8 | threshold of P < 0.005, extant threshold = 10 voxels | 5 |
| Mathur, V. A. (African-American Group) | 2010 | 14 | 3 | SPM2 | 8 | threshold of p < 0.005, extant threshold = 10 voxels | 5 |
| Mathur, V. A. (Caucasian-American Group) | 2012 | 10 | 3 | SPM2 | 8 | threshold of P < 0.005, extant threshold = 10 voxels | 5 |
| Mathur, V. A. (Caucasian-American Group) | 2010 | 14 | 3 | SPM2 | 8 | threshold of p < 0.005, extant threshold = 10 voxels | 5 |
| Mathur, V. A. (Full Sample) | 2010 | 28 | 3 | SPM2 | 8 | threshold of p < 0.005, extant threshold = 10 voxels | 6 |
| Mattan, B. D. | 2018 | 60 | 3 | SPM8 | 8 | voxel-level threshold of P < 0.001 with a cluster extent threshold of 53 voxels | 6 |
| McCutcheon, R. (Black Group) | 2018 | 17 | 3 | SPM8 | 8 | peak threshold p < 0.001, cluster threshold p < 0.05 FWE corrected | 6 |
| McCutcheon, R. (White Group) | 2018 | 19 | 3 | SPM8 | 8 | peak threshold p < 0.001, cluster threshold p < 0.05 FWE corrected | 6 |
| Mitchell, G. P. | 2006 | 15 | 1.5 | SPM99 | 8 | experiment-wise threshold of p < 0.05, corrected for multiple comparisons | 4 |
| Molapour, T. | 2015 | 20 | 3 | SPM8 | 8 | uncorrected threshold of (p < .001) | 4 |
| Molenberghs, P. | 2017 | 40 | 3 | SPM8 | 9 | cluster-level threshold with a familywise error (FWE) rate (p) of less than .05, corrected for multiple comparisons | 7 |
| Molenberghs, P. | 2016 | 48 | 3 | SPM8 | 6 | cluster-level or voxel-level threshold with an FWE or FDR rate of P < 0.05 corrected for the whole brain, with clusters defined by a voxel-level probability threshold of P < 0.001 and a minimum cluster size of 20 voxels | 7 |
| Molenberghs, P. | 2013 | 24 | 1.5 | SPM5 | 7 | cluster‐level probability threshold of P FWE < 0.05 corrected for the whole‐brain search volume (with clusters defined by the voxel‐level threshold P < 0.001) | 5 |
| Molenberghs, P. and Bosworth, R. (1) | 2014 | 48 | 3 | SPM8 | 6 | cluster‐level threshold with a familywise error rate (FWE) of P < 0.05 corrected for the whole brain was used to identify significant activation, with clusters defined by a voxel‐level probability threshold of P < 0.001 | 7 |
| Molenberghs, P. and Bosworth, R. (2) | 2014 | 48 | 3 | SPM8 | 6 | cluster‐level threshold with a familywise error rate (FWE) of P < 0.05 corrected for the whole brain was used to identify significant activation, with clusters defined by a voxel‐level probability threshold of P < 0.001 | 7 |
| Molenberghs, P. and Morrison, S. | 2014 | 20 | 3 | SPM8 | 6 | familywise error (FWE) rate of P <0.05, was used to define significant activation for all contrast analyses, and a voxel-level probability threshold of P <0.001 | 6 |
| Moradi, Z. | 2017 | 20 | 3 | SPM8 | 8 | P < 0.001 uncorrected at the whole brain level and an extent threshold of >70 voxels | 5 |
| Morrison, S. | 2012 | 20 | 3 | SPM8 | 6 | familywise error rate (FWE) of p<0.05, was used to define significant activation for all analyses, and a voxel-level probability threshold of p<.001 was used to define each cluster | 6 |
| Newman-Norlund, R. D. | 2008 | 22 | 3 | SPM2 | 6 | P < 0.001, uncorrected for multiple comparisons, minimum cluster size of 10 | 5 |
| Nugiel, T. | 2020 | 50 | 3 | FSL | 8 | cluster threshold of z > 3.1, p < .05 | 6 |
| Raghunath, B. L. | 2022 | 27 | 3 | SPM12 | 9 | p < 0.05 with FWE correction at the voxel level | 7 |
| Rauchbauer, B. | 2015 | 41 | 3 | SPM8 | 8 | Threshold p = .05, cluster level multiple comparison correction (selection threshold p = .001) | 6 |
| Richeson, J. A. | 2003 | 15 | 1.5 | SPM99 | 6 | thresholded at P < 0.005 corrected, minimal cluster size of 10 voxels | 5 |
| Richins, M. T. | 2019 | 69 | 1.5 | FSL | 5 | p < .05, family-wiseerror rate (FWER) corrected with a cluster-extent basedthresholding method | 6 |
| Ronquillo, J. | 2007 | 11 | 3 | BrainVoyagerQX | not provided | Threshold at P < 0.01, 20 voxel extent | 4 |
| Rubien-Thomas, E. | 2021 | 106 | 3 | FSL | 5 | corrected cluster significance threshold of Z > 3.1 and cluster significance threshold of p < 0.05 | 6 |
| Ruckmann, J. (1) | 2015 | 30 | 3 | SPM8 | 8 | p≤0.05 and a minimum cluster size of k≥50 were set | 6 |
| Ruckmann, J. (2) | 2015 | 30 | 3 | SPM8 | 8 | p≤0.05 and a minimum cluster size of k≥50 were set | 6 |
| Rule, N. O. | 2010 | 28 | 3 | BrainVoyagerQX | 7 | voxelwise p < 0.005, minimum cluster size > five functional voxels | 6 |
| Scheepers, D. | 2013 | 41 | 3 | SPM8 | 8 | p < 0.0005 (uncorrected) and a contiguity threshold of 20 voxels | 6 |
| Sheng, F. | 2014 | 21 | 3 | SPM8 | 8 | threshold of (P < 0.05, FDR-corrected) | 6 |
| Steines, M. (1) | 2020 | 22 | 3 | SPM12 | 8 | p < .05, cluster FWE-corrected | 6 |
| Steines, M. (2) | 2020 | 22 | 3 | SPM12 | 8 | p < .05, cluster FWE-corrected | 6 |
| Telzer, E. H. | 2015 | 26 | 3 | SPM8 | 8 | voxel-wise threshold of p < .005 combined with a minimum cluster size of 42 voxels, corresponding to p < .05, False Wise Error (FWE) corrected | 7 |
| Van Bavel, J. J. (1) | 2008 | 17 | 3 | SPM5 | 9 | p value of at least .99, p ≤ .001 (uncorrected), and a significant effect was reported if activity in at least 10 contiguous voxels | 5 |
| Van Bavel, J. J. (2) | 2008 | 17 | 3 | SPM5 | 9 | p value of at least .99, p ≤ .001 (uncorrected), and a significant effect was reported if activity in at least 10 contiguous voxels | 5 |
| van Gils, S. | 2020 | 17 | 3 | BrainVoyager 21.4 | 8 | A primary voxelwise threshold of p < .001 (uncorrected) to form clusters, and a cluster-threshold estimation procedure (Forman et al., 1995) to derive minimum cluster sizes that correspond to a corrected confidence level of α = .05. | 5 |
| Volz, K. G. | 2009 | 20 | 3 | LIPSIA | 9 | voxelwise threshold of p < .001 (uncorrected) with a cluster extent threshold of ≥ 5 contiguous voxels | 5 |
| Wang, C. | 2015 | 30 | 3 | SPM8 | 8 | voxel-level threshold of P < 0.001 and an extent threshold k > 20 voxels | 6 |
| Watson, R. | 2017 | 21 | 3 | BrainVoyagerQX | 6 | p < 0.05 FDR corrected for cluster size | 6 |
| Wu, C. T. | 2018 | 54 | 3 | SPM8 | 10 | corrected statistical threshold of p < 0.05 achieved by a voxel-wise p < 0.001 and an extent threshold of k > 70 voxels | 5 |
| Xu, X. (Caucasian Group) | 2009 | 16 | 3 | SPM2 | 8 | voxel threshold of p < 0.001 and a spatial extent threshold of k = 50 | 5 |
| Xu, X. (Chinese Group) | 2009 | 17 | 3 | SPM2 | 8 | voxel threshold of p < 0.001 and a spatial extent threshold of k = 50 | 5 |
| Yan, Z. (1) | 2019 | 44 | 3 | SPM12 | 9 | voxel-wise p < 0.05 FWE-corrected, k ≥ 10 | 7 |
| Yan, Z. (2) | 2019 | 44 | 3 | SPM12 | 9 | voxel-wise p < 0.05 FWE-corrected, k ≥ 10 | 7 |
| Zuo, S. | 2013 | 20 | 3 | SPM8 | 8 | voxel threshold of p < 0.001 and a spatial extent threshold of k = 50 | 5 |
| Fang, Z. | 2024 | 25 | 3 | SPM12 | 8 | GRF corrected for multiple comparisons, with voxel p < 0.001; cluster p < 0.05; two-tailed | 6 |
| Mei, S. | 2025 | 40 Controls, 40 Test | 3 | SPM12 | 4 | Voxel-level threshold P < 0.001, cluster-level P < 0.05, FWE corrected | 7 |
| Zacharopoulos, G. | 2023 | 20 | 3 | SPM12 | 8 | Whole-brain voxel-wise PFWE<.05 threshold | 6 |
| Mauchaund, M. (1) | 2023 | 24 | 3 | SPM12 | 5 | Family-Wise-Error (FWE) corrected at P < 0.05 cluster-wise | 6 |
| Mauchaund, M. (2) | 2023 | 24 | 3 | SPM12 | 5 | Family-Wise-Error (FWE) corrected at P < 0.05 cluster-wise | 6 |

Note. Quality assessment scoring criteria for each experiment included in the meta-analysis. Sample size: <15 participants (0, unless block design), 15-25 (1), >25 (2). Scanner field strength: 1.5T (0), 3T (1), >3T (2). Analysis software: non-validated/undocumented versions (0), standard validated software (1). Smoothing kernel: not reported or >3x voxel size (0), reported and 2-3x voxel size (1). Statistical correction: uncorrected or not reported (0), cluster extent threshold (1), FWE/FDR or other correction methods (2). Total score ranges from 0-8 points. Each row represents an individual experiment; articles contributing multiple experiments are indicated by numbers in parentheses. Block designs were given exemption for small sample sizes due to their higher statistical power. Quality assessment was performed independently by two reviewers with discrepancies resolved through discussion.
