# Supplementary material for: A Meta‐Analysis of Functional Magnetic Resonance Imaging Studies on In‐Group and Out‐Group Categorization
Source: Brain Behav. 2026 Mar 31;16(4):e71314. doi: 10.1002/brb3.71314 (PMC13112008; doi:10.1002/brb3.71314)
Supplement: Supplementary file 2 — Supplementary Materials: brb371314‐sup‐0002‐SuppMat.docx [file BRB3-16-e71314-s002.docx]

**Underpowered Results**

**Group Memberships**

***Ethnicity, OG>IG***

Though underpowered, across all studies (n = 19), there were 28 experiments (458 subjects, 171 foci, 5 out-of-mask foci) for bias against Ethnicity out-group (OG>IG) analysis. Analysis for Ethnicity (OG>IG) revealed 15 significant clusters (Table S2.1; Figure S2.1), with the largest (1312 mm^3^) located in the left posterior fusiform gyrus (occipital face area; OFA). Other clusters included the right inferior temporal gyrus, bilateral insula, left inferior parietal lobule, right inferior occipital gyrus, and right precentral gyrus.

**Table S2.1**

*ALE results of Ethnicity group, OG>IG*

| Cluster | Volume (mm^3^) | Max ALE (x10^-2^) | MNI Coordinates | | | Label |
| --- | --- | --- | --- | --- | --- | --- |
|  |  |  | x | y | z |  |
| 1 | 1312 | 1.44 | -30 | -80 | -16 | Left Posterior Fusiform Gyrus (Occipital Face Area) |
| 2 | 824 | 1.73 | 50 | -54 | 0 | Right Inferior Temporal Gyrus |
| 3 | 584 | 1.34 | -28 | 22 | 12 | Left Insula |
| 4 | 584 | 1.31 | -50 | -34 | 54 | Left Inferior Parietal Lobule |
| 5 | 464 | 1.16 | 30 | -94 | -8 | Right Inferior Occipital Gyrus |
| 6 | 432 | 1.04 | 50 | 2 | 28 | Right Precentral Gyrus |
|  |  | 0.98 | 46 | -2 | 30 | Right Precentral Gyrus |
|  |  | 0.96 | 52 | 8 | 26 | Right Inferior Frontal Gyrus |
| 7 | 424 | 1.03 | 32 | 24 | 4 | Right Insula |
|  |  | 0.95 | 38 | 26 | -12 | Right Inferior Frontal Gyrus |
|  |  | 0.89 | 38 | 24 | -20 | Right Inferior Frontal Gyrus |
|  |  | 0.84 | 34 | 28 | -4 | Right Insula |
| 8 | 416 | 1.27 | 2 | 10 | 46 | Left Medial Frontal Gyrus |
| 9 | 360 | 1.27 | 52 | 0 | 46 | Right Precentral Gyrus |
| 10 | 304 | 1.12 | 32 | -2 | -18 | Right Parahippocampal Gyrus/ Amygdala |
| 11 | 304 | 1.15 | 32 | 30 | 42 | Right Middle Frontal Gyrus |
| 12 | 256 | 0.90 | -30 | -78 | 20 | Left Middle Occipital Gyrus |
|  |  | 0.88 | -38 | -82 | 20 | Left Middle Occipital Gyrus |
|  |  | 0.87 | -22 | -76 | 24 | Left Precuneus |
| 13 | 248 | 1.02 | -44 | 50 | 4 | Left Middle Frontal Gyrus |
| 14 | 240 | 1.04 | -2 | 0 | 68 | Left Superior Frontal Gyrus |
| 15 | 224 | 1.01 | -30 | -58 | -12 | Left Cerebellum/Declive |

*Note*: All clusters reported survived a voxel-level uncorrected threshold of *p* < .005, with a minimum cluster size of 200 mm^3^. Coordinates are reported in the Montreal Neurological Institute (MNI) convention. ALE = Activation Likelihood Estimate.

**Figure S2.1**

*Activation map of Ethnicity group, OG>IG*

*
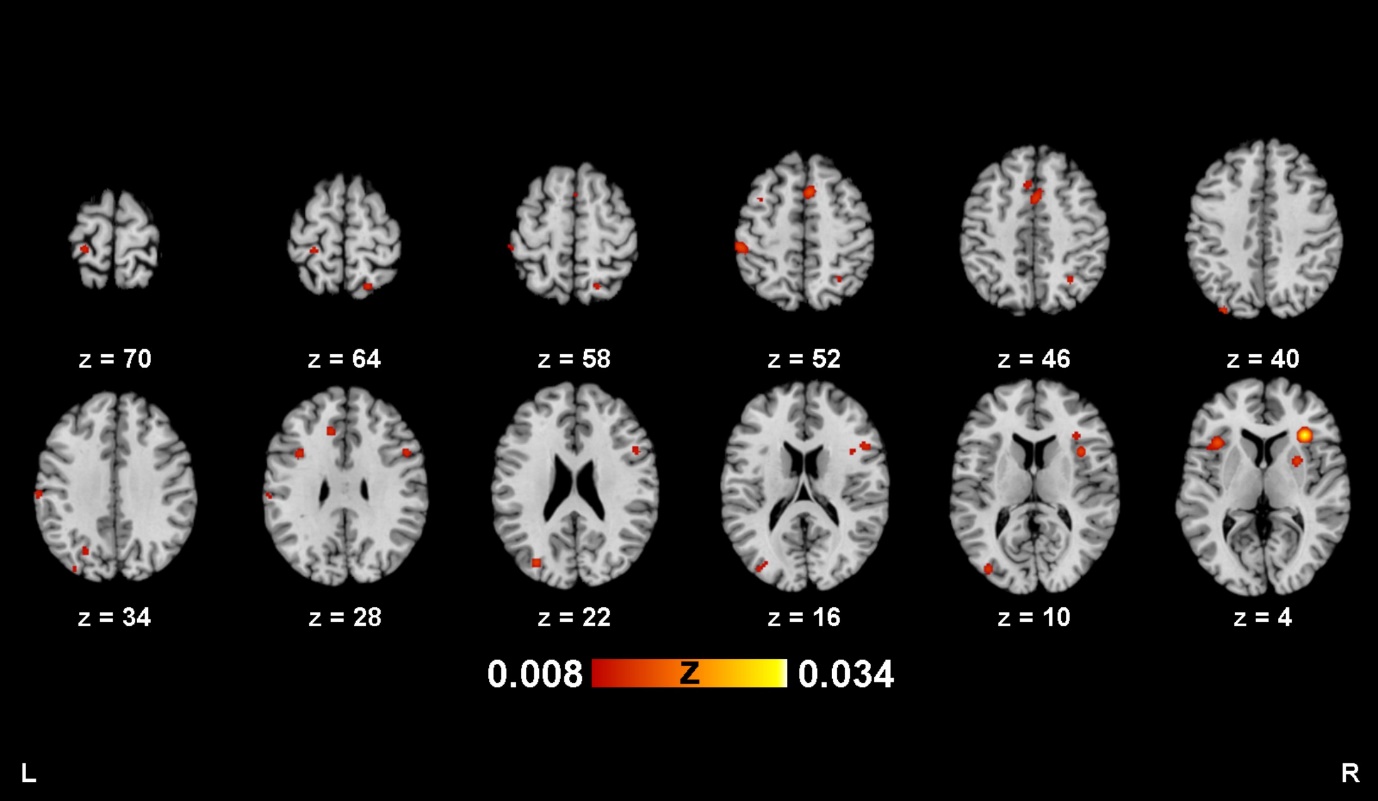
*

*Note.* Activation maps were thresholded at *p* < .005 (uncorrected), with a minimum cluster size of 200 mm^3^. Images are in neurological convention.

***Nationality Group***

Though underpowered, across all studies (n = 7), there were 12 experiments (221 subjects, 65 foci, 1 out-of-mask foci) for bias toward Nationality in-group (IG>OG) analysis. Analysis for Nationality (IG>OG) revealed 11 significant clusters (Table S2.2a; Figure S2.2a), with the largest (2000 mm^3^) located in the left insula, left frontal orbital cortex, and left inferior frontal gyrus. Other clusters included the right fusiform gyrus, left medial frontal gyrus, right inferior parietal lobule, right cingulate gyrus, right middle frontal gyrus, right middle temporal gyrus, left medial frontal, left lingual gyrus, right supramarginal gyrus, and right precuneus.

Across all studies (n = 6), there were 19 experiments (229 subjects, 116 foci, 2 out-of-mask foci) for bias against Nationality out-group (OG>IG) analysis. Analysis for Nationality (OG>IG) revealed 16 significant clusters (Table S2.2b; Figure S2.2b), with the largest (1224 mm^3^) located in the right superior frontal gyrus. Other clusters included the right insula, right inferior parietal lobule, right precentral gyrus, right superior temporal gyrus, left insula, left middle occipital gyrus, right superior parietal lobule, left postcentral gyrus, left fusiform gyrus, left superior occipital gyrus, left inferior frontal gyrus, left inferior occipital gyrus, and left precuneus.

**Table S2**

*ALE results of Nationality group, (a) IG>OG, (b) OG>IG*

| (a) Nationality, IG>OG | | |  |  |  |  |
| --- | --- | --- | --- | --- | --- | --- |
| Cluster | Volume (mm^3^) | Max ALE (x10^-2^) | MNI Coordinates | | | Label |
|  |  |  | x | y | z |  |
| 1 | 2000 | 1.48 | -32 | 16 | -4 | Left Insula |
|  |  | 0.98 | -24 | 8 | -20 | Left Frontal Orbital Cortex |
|  |  | 0.94 | -28 | 16 | -14 | Left Insula |
|  |  | 0.84 | -38 | 26 | -8 | Left Inferior Frontal Gyrus |
|  |  | 0.83 | -30 | 26 | -14 | Left Inferior Frontal Gyrus |
| 2 | 1160 | 2.09 | 46 | -48 | -16 | Right Fusiform Gyrus |
| 3 | 728 | 1.54 | -2 | 14 | 44 | Left Medial Frontal Gyrus |
| 4 | 688 | 1.55 | 46 | -26 | 46 | Right Inferior Parietal Lobule |
| 5 | 600 | 1.31 | 10 | 4 | 48 | Right Cingulate Gyrus |
| 6 | 528 | 1.05 | 46 | 8 | 40 | Right Middle Frontal Gyrus |
| 7 | 488 | 0.97 | 40 | -64 | 14 | Right Middle Temporal Gyrus |
|  |  | 0.90 | 42 | -68 | 16 | Right Middle Occipital Gyrus |
| 8 | 376 | 0.92 | -8 | 38 | 34 | Left Medial Frontal Gyrus |
|  |  | 0.82 | -6 | 30 | 40 | Left Cingulate Gyrus |
| 9 | 216 | 0.96 | -12 | -64 | -6 | Left Lingual Gyrus |
| 10 | 216 | 0.97 | 40 | -46 | 38 | Right Supramarginal Gyrus |
| 11 | 216 | 0.96 | 30 | -78 | 44 | Right Precuneus |
| (b) Nationality, OG>IG | | |  |  |  |  |
| 1 | 1224 | 1.73 | 6 | 14 | 50 | Right Superior Frontal Gyrus |
| 2 | 1008 | 2.15 | 34 | 26 | 2 | Right Insula |
| 3 | 864 | 1.24 | 64 | -44 | 24 | Right Inferior Parietal Lobule |
|  |  | 1.04 | 60 | -50 | 26 | Right Supramarginal Gyrus |
|  |  | 0.92 | 54 | -44 | 28 | Right Inferior Parietal Lobule |
| 4 | 664 | 1.27 | 56 | 0 | 6 | Right Precentral Gyrus |
|  |  | 0.91 | 62 | -2 | -2 | Right Superior Temporal Gyrus |
| 5 | 640 | 1.15 | 54 | -20 | -2 | Right Superior Temporal Gyrus |
|  |  | 0.91 | 64 | -18 | 2 | Right Superior Temporal Gyrus |
| 6 | 552 | 1.49 | -38 | 20 | 0 | Left Insula |
| 7 | 544 | 1.58 | -38 | -84 | 12 | Left Middle Occipital Gyrus |
| 8 | 536 | 1.51 | 18 | -64 | 62 | Right Superior Parietal Lobule |
| 9 | 512 | 1.48 | -24 | -34 | 68 | Left Postcentral Gyrus |
| 10 | 496 | 1.25 | -50 | -62 | -10 | Left Fusiform Gyrus |
| 11 | 496 | 1.55 | -32 | -84 | 38 | Left Superior Occipital Gyrus |
| 12 | 488 | 1.22 | 50 | -4 | 4 | Right Insula |
| 13 | 464 | 1.19 | -36 | 10 | 28 | Left Inferior Frontal Gyrus |
| 14 | 456 | 1.11 | -44 | -74 | -6 | Left Inferior Occipital Gyrus |
| 15 | 280 | 1.04 | -24 | -70 | 34 | Left Precuneus |
| 16 | 232 | 0.89 | -50 | -28 | 40 | Left Postcentral Gyrus |
|  |  | 0.87 | -44 | -26 | 46 | Left Inferior Parietal Lobule |

*Note*: All clusters reported survived a voxel-level uncorrected threshold of *p* < .005, with a minimum cluster size of 200 mm^3^. Coordinates are reported in the Montreal Neurological Institute (MNI) convention. ALE = Activation Likelihood Estimate.

**Figure S2.2**

*Activation map of Nationality group, (a) IG>OG, (b), OG>IG*

***
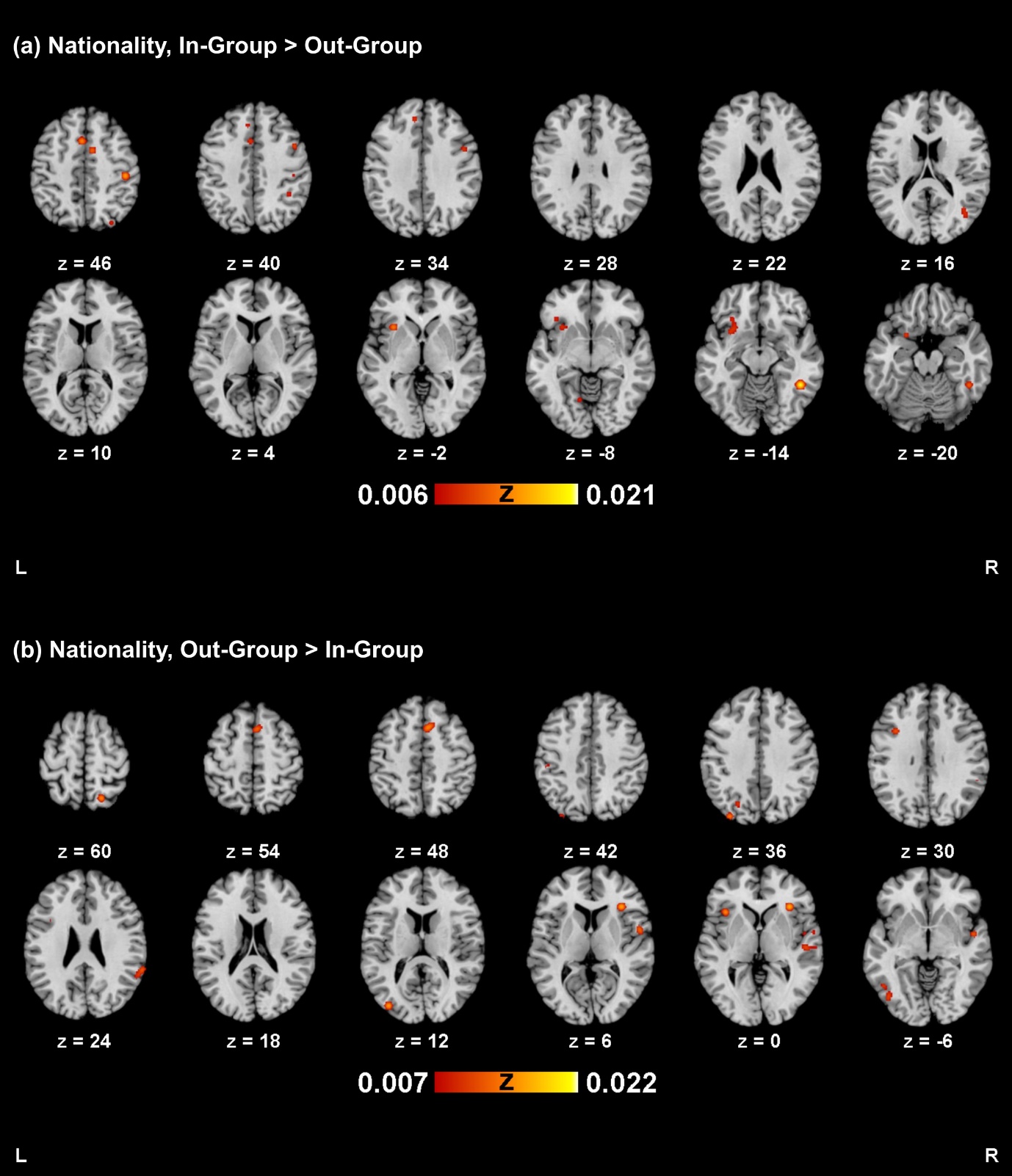
***

*Note.* Activation maps were thresholded at *p* < .005 (uncorrected), with a minimum cluster size of 200 mm^3^. Images are in neurological convention.

**fMRI Task Types**

***Empathy Processing, OG>IG***

Though underpowered, across all studies (n = 14), there were 24 experiments (350 subjects, 167 foci, 2 out-of-mask foci) for bias during empathy processing tasks against out-group (OG>IG) analysis. Analysis for OG>IG revealed 25 significant clusters (Table S2.3; Figure S2.3), with the largest (1656 mm^3^) located in the right insula. Other clusters included the right superior frontal gyrus, left insula, left middle occipital gyrus, left inferior parietal lobule, left inferior frontal gyrus, left fusiform gyrus, and left postcentral gyrus.

**Table S2.3**

*ALE results of Empathy Processing task, OG>IG*

| Cluster | Volume (mm^3^) | Max ALE (x10^-2^) | MNI Coordinates | | | Label |
| --- | --- | --- | --- | --- | --- | --- |
|  |  |  | x | y | z |  |
| 1 | 1656 | 3.37 | 34 | 26 | 4 | Right Insula |
| 2 | 1536 | 1.70 | 40 | 12 | 12 | Right Insula |
|  |  | 1.44 | 50 | 12 | 26 | Right Inferior Frontal Gyrus |
|  |  | 1.18 | 50 | 16 | 16 | Right Inferior Frontal Gyrus |
| 3 | 1384 | 1.69 | 6 | 14 | 50 | Right Superior Frontal Gyrus |
| 4 | 1232 | 2.02 | -38 | 20 | 2 | Left Insula |
| 5 | 1008 | 1.59 | -36 | -84 | 12 | Left Middle Occipital Gyrus |
|  |  | 1.58 | -30 | -78 | 20 | Left Middle Occipital Gyrus |
| 6 | 920 | 1.61 | -52 | -32 | 54 | Left Inferior Parietal Lobule |
|  |  | 0.90 | -44 | -42 | 56 | Left Inferior Parietal Lobule |
| 7 | 512 | 1.23 | -36 | 10 | 30 | Left Inferior Frontal Gyrus |
|  |  | 0.89 | -40 | 2 | 32 | Left Precentral Gyrus |
| 8 | 480 | 1.13 | -30 | -76 | -16 | Left Fusiform Gyrus |
| 9 | 432 | 1.54 | -12 | -28 | -44 | Left Cerebellum |
| 10 | 432 | 1.51 | 18 | -64 | 62 | Right Superior Parietal Lobule |
| 11 | 432 | 1.48 | -24 | -34 | 68 | Left Postcentral Gyrus |
| 12 | 416 | 1.44 | 28 | 4 | 4 | Right Basal Ganglia |
| 13 | 400 | 1.55 | -32 | -84 | 38 | Left Superior Occipital Gyrus |
| 14 | 392 | 1.32 | -62 | -22 | 32 | Left Inferior Parietal Lobule |
| 15 | 384 | 1.23 | -48 | -60 | -4 | Left Inferior Temporal Gyrus |
| 16 | 384 | 1.34 | 30 | -58 | 48 | Right Superior Parietal Lobule |
| 17 | 368 | 1.17 | 54 | -56 | -2 | Right Middle Temporal Gyrus |
| 18 | 368 | 1.26 | -12 | 28 | 28 | Left Cingulate Gyrus |
| 19 | 320 | 1.20 | -44 | -48 | -18 | Left Fusiform Gyrus |
|  |  |  |  |  |  |  |
| 20 | 312 | 1.11 | -4 | 20 | 44 | Left Medial Frontal Gyrus |
| 21 | 304 | 1.11 | -44 | -74 | -6 | Left Inferior Occipital Gyrus |
| 22 | 256 | 0.99 | 32 | -74 | -8 | Right Lingual Gyrus |
| 23 | 248 | 1.00 | -36 | 6 | 50 | Left Middle Frontal Gyrus |
| 24 | 200 | 0.99 | -4 | -44 | -8 | Left Cerebellum |
| 25 | 200 | 1.04 | -24 | -70 | 34 | Left Precuneus |

*Note*: All clusters reported survived a voxel-level uncorrected threshold of *p* < .005, with a minimum cluster size of 200 mm^3^. Coordinates are reported in the Montreal Neurological Institute (MNI) convention. ALE = Activation Likelihood Estimate.

**Figure 2.3**

*Activation map of Empathy Processing task, OG>IG*

*
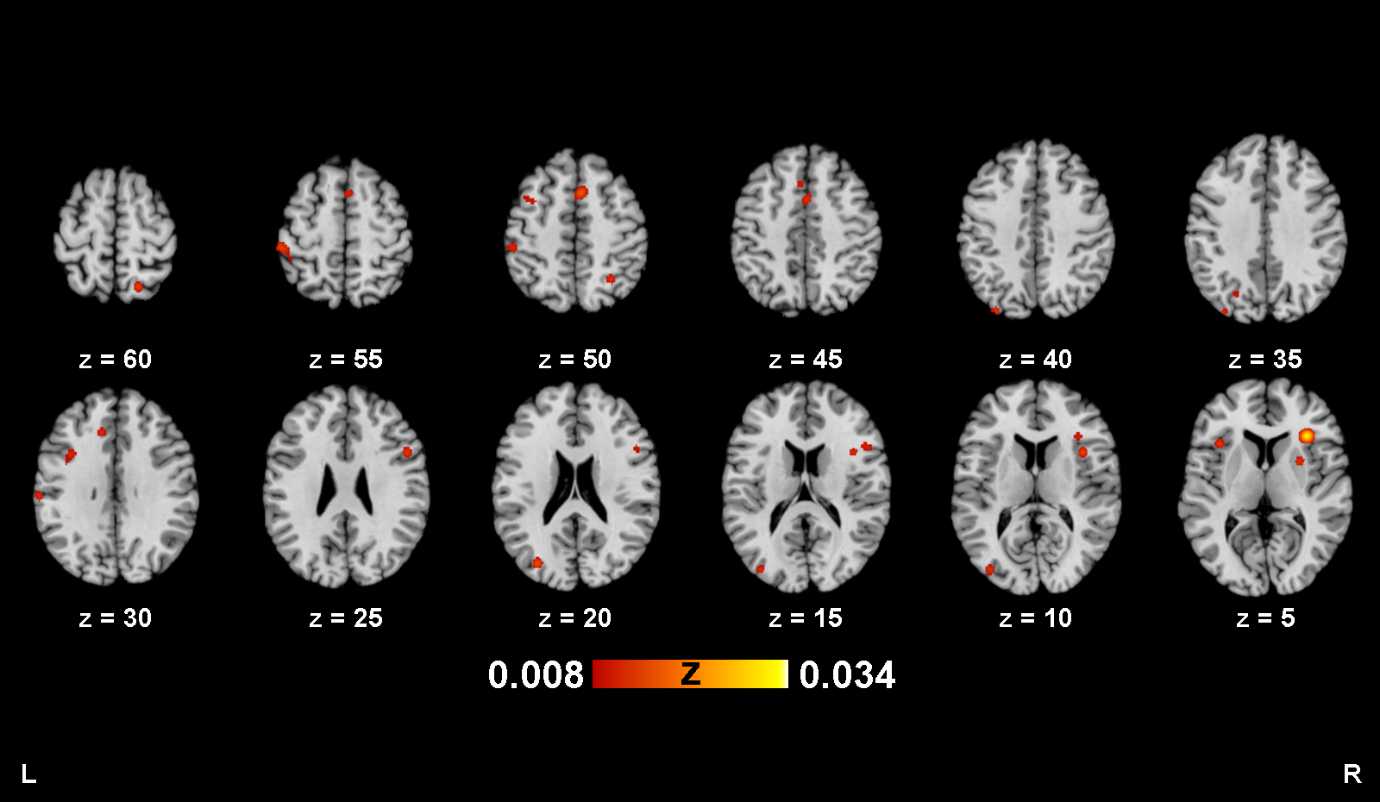
*

*Note.* Activation maps were thresholded at *p* < .005 (uncorrected), with a minimum cluster size of 200 mm^3^. Images are in neurological convention.

***Face Processing Task***

Though underpowered, across all studies (n = 15), there were 19 experiments (521 subjects, 93 foci, 3 out-of-mask foci) for bias during face processing tasks toward in-group (IG>OG) analysis. Analysis for IG>OG revealed 7 significant clusters (Table 2.4a; Figure 2.4a), with the largest (640 mm^3^) located in the right inferior parietal lobule. Other clusters included the bilateral inferior occipital gyrus, right medial frontal gyrus, right middle temporal gyrus, right middle frontal gyrus, and right middle occipital gyrus.

Across all studies (n = 12), there were 15 experiments (274 subjects, 80 foci, 5 out-of-mask foci) for bias during face processing tasks against out-group (OG>IG) analysis. Analysis for OG>IG revealed 11 significant clusters (Table 2.4b; Figure 2.4b), with the largest (1136 mm^3^) located in the left cingulate gyrus, and right anterior cingulate gyrus. Other clusters included the bilateral insula, left superior frontal gyrus, right medial frontal gyrus, left middle temporal gyrus, right inferior occipital gyrus, and right parahippocampal gyrus.

**Table S2.4**

*ALE results of Face Processing task, (a) IG>OG, (b) OG>IG*

| (a) Face Processing, IG>OG | | |  |  |  |  |
| --- | --- | --- | --- | --- | --- | --- |
| Cluster | Volume (mm^3^) | Max ALE (x10^-2^) | MNI Coordinates | | | Label |
|  |  |  | X | y | z |  |
| 1 | 640 | 1.80 | 38 | -32 | 38 | Right Inferior Parietal Lobule |
| 2 | 616 | 1.50 | 38 | -72 | 0 | Right Inferior Occipital Gyrus |
| 3 | 560 | 1.35 | -42 | -74 | 0 | Left Inferior Occipital Gyrus |
| 4 | 472 | 1.18 | 6 | 18 | 46 | Right Medial Frontal Gyrus |
| 5 | 352 | 0.97 | 58 | -40 | -10 | Right Middle Temporal Gyrus |
| 6 | 328 | 0.90 | 50 | 38 | 16 | Right Middle Frontal Gyrus |
|  |  | 0.90 | 54 | 38 | 8 | Right Middle Frontal Gyrus |
| 7 | 304 | 0.99 | 30 | -88 | 24 | Right Middle Occipital Gyrus |
|  |  | 0.87 | 24 | -82 | 28 | Right Cuneus |
| (b) Face Processing, OG>IG | | |  |  |  |  |
| 1 | 1136 | 1.14 | 0 | 16 | 32 | Left Cingulate Gyrus |
|  |  | 0.79 | 8 | 24 | 26 | Right Anterior Cingulate Gyrus |
| 2 | 992 | 1.22 | 32 | 26 | -6 | Right Insula |
| 3 | 832 | 1.07 | -2 | 0 | 68 | Left Superior Frontal Gyrus |
|  |  | 0.89 | 6 | -2 | 62 | Right Medial Frontal Gyrus |
| 4 | 704 | 1.50 | 6 | 14 | 48 | Right Medial Frontal Gyrus |
| 5 | 512 | 0.94 | 42 | 8 | 0 | Right Insula |
| 6 | 336 | 0.83 | -36 | -54 | 30 | Left Middle Temporal Gyrus |
|  |  | 0.76 | -34 | -52 | 40 | Left Inferior Parietal Lobule |
| 7 | 288 | 0.87 | -36 | 14 | 2 | Left Insula |
|  |  | 0.76 | -30 | 22 | 6 | Left Insula |
| 8 | 264 | 0.90 | 36 | -96 | -4 | Right Inferior Occipital Gyrus |
| 9 | 256 | 0.90 | 33 | -51 | 9 | Right Parahippocampal Gyrus |
| 10 | 224 | 0.97 | 6 | 60 | 10 | Right Medial Frontal Gyrus |
| 11 | 208 | 0.90 | -60 | 8 | -4 | Left Superior Temporal Gyrus |

*Note*: All clusters reported survived a voxel-level uncorrected threshold of *p* < .005, with a minimum cluster size of 200 mm^3^. Coordinates are reported in the Montreal Neurological Institute (MNI) convention. ALE = Activation Likelihood Estimate.

**Figure S2.4**

*Activation map of Face Processing task, (a) IG>OG, (b), OG>IG*

**
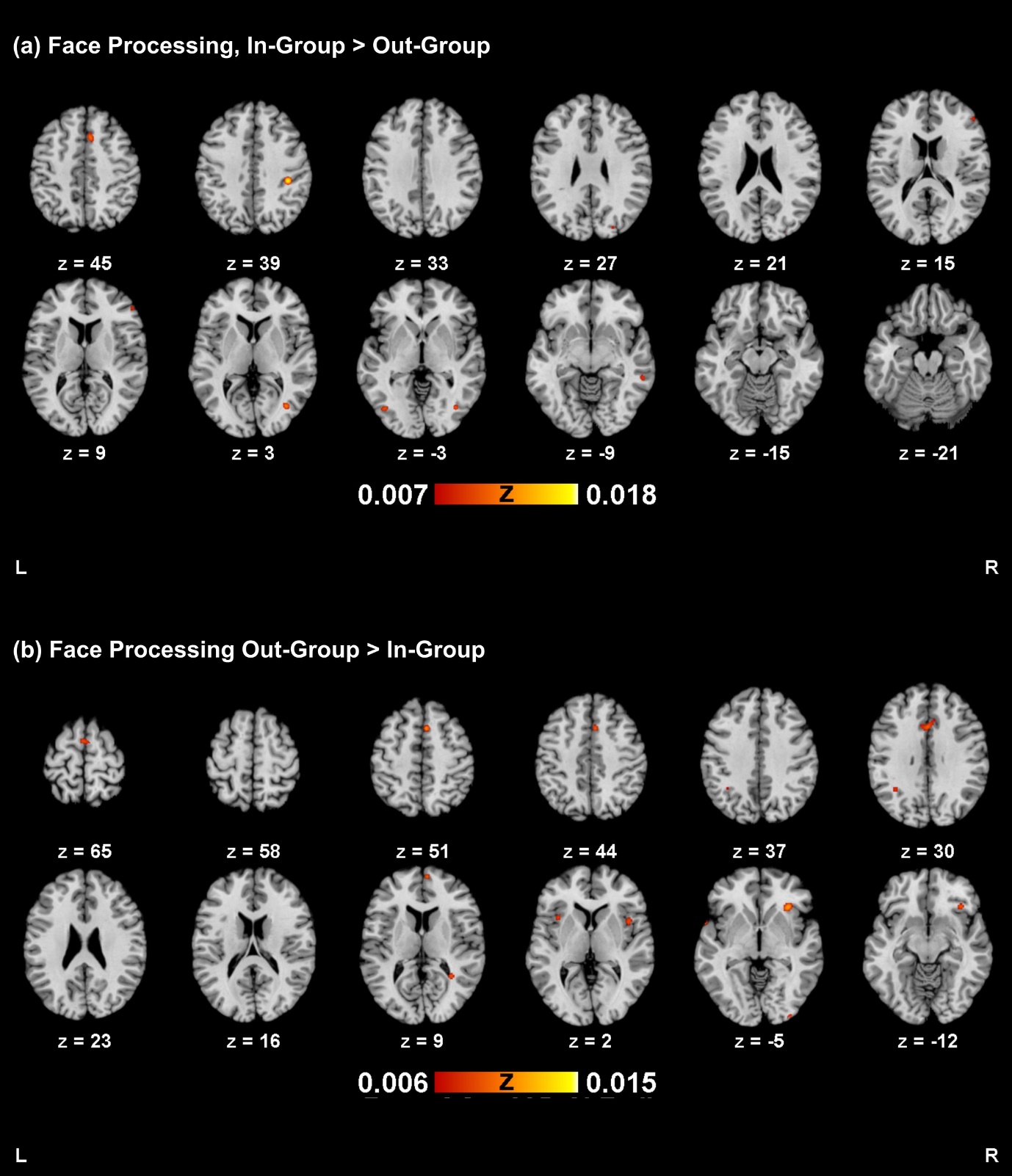
**

*Note.* Activation maps were thresholded at *p* < .005 (uncorrected), with a minimum cluster size of 200 mm^3^. Images are in neurological convention.

**Cluster-level FWE Correction**

We performed cluster-level FWE correction using a cluster-forming threshold of p < .001 (uncorrected), cluster-level threshold of p < .05 (FWE-corrected), with 10,000 permutations for all main analyses: (1) IG>OG (Table S2.5a), (2) OG>IG (Table S2.5b), (3) Ethnicity IG>OG (Table S2.5c), (4) Empathy IG>OG (Table S2.5d).

**Table S2.5**

*Cluster-Level FWE corrected ALE results of (a) In-Group > Out-Group Processing, (b) Out-Group > In-Group Processing, (c) Ethnicity (In-Group > Out-Group), (d) Empathy (In-Group > Out-Group)*

| Cluster | Volume (mm^3^) | Max ALE (x10^-2^) | MNI Coordinates | | | Label |
| --- | --- | --- | --- | --- | --- | --- |
|  |  |  | x | y | z |  |
| (a) In-Group > Out-Group | | | | | | |
| 1 | 1520 | 2.73 | -32 | 18 | -4 | Left Insula |
|  |  | 1.94 | -32 | 20 | -14 | Left Inferior Frontal Gyrus |
|  |  | 1.73 | -24 | 8 | -20 | Left Uncus |
| (b) Out-Group > In-Group | | | | | | |
| 1 | 1120 | 3.48 | 34 | 26 | 4 | Right Insula |
| 2 | 1096 | 3.11 | 6 | 14 | 50 | Right Presupplementary Motor Area |
| (c) Ethnicity (In-Group > Out-Group) | | | | | | |
| 1 | 864 | 2.32 | 26 | -80 | 28 | Right Superior Occipital Gyrus |
|  |  | 1.42 | 34 | -84 | 30 | Right Superior Occipital Gyrus |
| (d) Empathy (In-Group > Out-Group) | | | | | | |
| 1 | 896 | 1.79 | 58 | -26 | 44 | Right Postcentral Gyrus |
|  |  | 1.32 | 48 | -26 | 52 | Right Posterior Cingulate Gyrus |
| 2 | 840 | 1.74 | -54 | -30 | 28 | Left Inferior Parietal Lobule |
|  |  | 1.65 | -56 | -26 | 24 | Left Inferior Parietal Lobule |

*Note*: Coordinates are reported in the Montreal Neurological Institute (MNI) convention. ALE = Activation Likelihood Estimate.
